# New Insights Into the Evolution of Immune Adaptors in Murid Rodents

**DOI:** 10.1002/ece3.72851

**Published:** 2026-01-05

**Authors:** Qianqian Su, Zhenhua She, Yi Chen

**Affiliations:** ^1^ College of Forestry Central South University of Forestry and Technology Changsha China; ^2^ Department of Animal Management Changsha Ecological Zoo Changsha China

**Keywords:** evolutionary characterizations, immune adaptors, murids

## Abstract

The evolutionary dynamics of immune adaptors in wildlife remain poorly understood, despite their critical role in host–pathogen interactions. In this study, we investigated the molecular evolution of five TIR domain‐containing adaptors (MAL, MyD88, SARM, TRAM, and TRIF) in murid rodents across both interspecific and population levels to provide an integrative view of their evolutionary trajectories. Our analyses demonstrate that these adaptors are predominantly under purifying selection in murids, yet contain specific positively selected sites in MAL and MyD88, indicating localized adaptive fine‐tuning. These positively selected amino acid sites exhibited substantial genetic divergence among murid species. Within populations, we observed reduced genetic diversity in the adaptor genes compared with noncoding regions across the whole genome in both *Rattus tanezumi* and *Rattus norvegicus* , and no significant signals of recent positive or negative selection were detected in either species. Notably, we identified identical or nearly identical allelic variants between these two rat species across all five adaptor genes. Collectively, this study delineates the evolution of immune adaptors in murids across both deep and recent timescales, advancing our understanding of adaptive evolution in multilevel immune systems.

## Graphical abstract

TIR domain‐containing adaptors were evolved under purifying selection in murine. Positively selected sites were identified in Mal and Myd88 genes. Reduced genetic diversity of each adaptor gene was observed in two rat populations. Shared polymorphisms were common phenomena in rat adaptors.

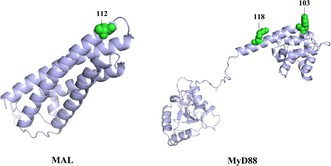

## 1 Introduction

Animals face constant threats from pathogens and associated infectious diseases throughout their lifespans (Siddle and Quintana‐Murci 2014). The immune system functions as a highly complex defense system, in which receptors, adaptors, and effectors serve as core components, enabling it to detect, analyze, and eliminate threats. Receptors, such as Toll‐like receptors (TLRs), provide specificity by effectively identifying pathogenic infections. When TLRs bind to ligands from parasites or viruses, they typically do not directly trigger downstream responses; instead, adaptors act as signaling intermediates. Adaptors are responsible for signal transduction and the activation of immune responses. Effectors, as the ultimate executors of immune function, kill and eliminate threats, ultimately establishing immune memory against the pathogen (O'Neill et al. 2003). Population genetic studies have demonstrated that immune‐related genes are key targets of natural selection, underscoring the critical role of immune mechanisms in host defense and advancing our understanding of how organisms respond to selective pressures (Siddle and Quintana‐Murci 2014). Extensive evolutionary research has focused on receptors genes (Fitzgerald and Kagan 2020; Su et al. 2022, 2024). However, different components of the immune system may exhibit distinct evolutionary patterns. Evidence has shown that adaptors experience stronger selection pressure than TLRs in healthy individuals from diverse geographic regions, suggesting greater evolutionary constraints on maintaining adaptor protein integrity (Fornarino et al. 2011).

To date, five adaptor molecules have been identified in mammals (Table 1). The most well‐characterized adaptor is the myeloid differentiation primary response gene 88 (MyD88). The remaining four adaptors include MyD88‐adaptor‐like (MAL, also known as TIRAP), TIR domain‐containing adaptor inducing interferon‐β (TRIF; also designated TICAM1), TRIF‐related adaptor molecule (TRAM; TICAM2), and sterile α‐ and armadillo‐motif‐containing protein (SARM). MyD88 was the first adaptor discovered to mediate signaling across multiple TLRs. Critical evidence stems from MyD88‐deficient mice, which failed to respond to ligands for TLR2, TLR4, TLR5, TLR7, or TLR9 (Takeuchi et al. 2000; O'Neill and Bowie 2007; Vijay 2018). MAL, the second adaptor identified, is essential for TLR2 and TLR4 signaling, functioning as a bridging molecule to recruit MyD88 (Horng et al. 2001). TRIF plays a pivotal role in TLR3‐ and TLR4‐mediated pathways in mammals (Yamamoto et al. 2003). In TRIF‐deficient mice, both TLR3‐ and TLR4‐dependent IFN‐β expression and IRF3 activation were impaired (Yamamoto et al. 2003). TRAM specifically contributes to the TLR4‐mediated MyD88‐independent pathway, as TRAM‐deficient mice exhibit defective cytokine production in response to TLR4 ligands but not other TLR ligands (Yammoto et al. 2003). In contrast, SARM negatively regulates TRIF‐dependent TLR signaling (Carty et al. 2006). Overexpression of SARM suppresses downstream TRIF‐mediated gene induction but does not affect MyD88‐dependent signaling (Carty et al. 2006).

**TABLE 1:** Description of Toll/IL‐1 receptor (TIR) domain‐containing adaptors in mammals.

Gene	Function	Protein Accession number ( *Rattus norvegicus* )	References
MyD88	Plays a central role in the innate and adaptive immune response and functions as an essential signal transducer in the interleukin‐1 and Toll‐like receptor signaling pathways	Q6Y1S1	Horng et al. (2001)
MAL	Is essential for TLR2 and TLR4 signaling, functioning as a bridging molecule to recruit MyD88	Q64349	Horng et al. (2001)
TRIF	Plays a pivotal role in TLR3‐ and TLR4‐mediated pathways	P27768	Yamamoto et al. (2003)
TRAM1	Contributes to the TLR4‐mediated MyD88‐independent pathway	Q5XI41	Yammoto et al. (2003)
SARM1	Negatively regulates TRIF‐dependent TLR signaling	D3ZUM2	Carty et al. (2006)

Previous work assessed the genetic diversity and evolutionary trajectories of these five TIR‐containing adaptors in human populations. MyD88 and TRIF exhibited signatures of purifying selection, consistent with their essential and nonredundant roles in early signal transduction (Fornarino et al. 2011). Additionally, multiple episodes of positive selection have shaped these adaptors, with distinct spatiotemporal patterns. Selective sweeps were detected in MyD88 and SARM across all human populations, whereas adaptive evolution in the other three adaptors was restricted to specific geographic groups (Fornarino et al. 2011). Nevertheless, the evolutionary history and genetic variation of these adaptors in wild mammals remain poorly understood, leaving significant gaps in our knowledge of their roles in natural populations.

In this study, we investigated genetic variation and molecular evolution of TIR‐containing adaptors in murid rodents (Rodentia: Muridae), the most widespread family of rats and mice. This family originated 20–30 million years ago, underwent rapid radiation into multiple subfamilies, and achieved a near‐global distribution (Musser and Carleton 2005). Their evolutionary success is evidenced by extensive adaptive radiation and global colonization (Puckett et al. 2016). Notably, murid rodents serve as asymptomatic reservoirs for at least 60 zoonotic pathogens (Baker et al. 2022). Commensal species like the Asian house rat ( *Rattus tanezumi* , RT) and brown rat ( *Rattus norvegicus* , RN) exhibit particularly close associations with human populations, facilitating transmission of pathogens including Hantavirus, *Yersinia*, *Leptospira*, *Salmonella*, and *Bartonella* (Wu et al. 2018; Su et al. 2022). This study addresses key knowledge gaps regarding TIR‐containing adaptor evolution in murid rodents. By characterizing evolutionary patterns in these species, we aim to enhance understanding of adaptor protein function in immune systems. Specifically, our objectives are to: (1) Determine evolutionary patterns of TIR‐containing adaptors across murid rodents through comparative genetic analysis; (2) Characterize evolutionary features of five adaptor genes in sympatric RT and RN populations using population genetic approaches.

## 2 Methods

### 2.1 Data Collection

We obtained full‐length sequences of five adaptor proteins from 14 different murid species from NCBI (https://www.ncbi.nlm.nih.gov/; accession numbers provided in Table 2). Attempts to extract DNA sequences from other murid species were partly unsuccessful due to low‐quality or incomplete genome data, which were consequently excluded. We performed sequence alignment using ClustalW (Codons) in MEGA6 (Tamura et al. 2013), with aligned sequences provided in Data S1–S5.

**TABLE 2:** List of TIR‐containing adaptor genes in murid rodents.

Species/Gene	Family	Genus	Myd88	MAL	TRIF	TRAM1	SARM1
*Rattus norvegicus*	Muridae	*Rattus*	NM_198130.2	NM_012798.2	NM_053588.3	NM_001007701.1	NM_001105817.1
*Rattus tanezumi*	Muridae	*Rattus*	GWHAOJD00000000 ^#^	#	#	#	#
*Rattus rattus*	Muridae	*Rattus*	XP_032767000.1	XP_032760060.1	XP_032756639.1	XP_032762685.1	XP_032768662.1
*Apodemus sylvaticus*	Muridae	*Apodemus*	XP_052042961.1	XP_052039523.1	XP_052047942.1	XP_052031901.1	XP_052051670.1
*Arvicanthis niloticus*	Muridae	*Arvicanthis*	XP_034339894.1	XP_034351580.1	XP_034352943.1	XP_034346132.1	XP_034362268.1
*Grammomys surdaster*	Muridae	*Grammomys*	XP_028623185.1	XP_028612765.1	XP_028618280.1	XP_028624996.1	XP_028615161.1
*Mastomys coucha*	Muridae	*Mastomys*	XP_031200586.1	XP_031227692.1	XP_031204546.1	XP_031222760.1	XP_031208423.1
*Mus caroli*	Muridae	*Mus*	XP_021026968.1	XP_021011762.1	XP_021004996.1	XP_021007224.1	XP_021032771.1
*Mus musculus*	Muridae	*Mus*	NP_034981.1	NP_034892.1	NP_778154.1	NP_082449.1	NP_001161993.1
*Mus pahari*	Muridae	*Mus*	XP_021062770	XP_021050000.1	XP_021074167.2	XP_021077761.1	XP_021068068.1
*Acomys russatus*	Muridae	*Acomys*	XM_051140057.1	XP_051000542.1	GCF_903995435.1*	XM_051158922.1	GCF_903995435.1*
*Psammomys obesus*	Muridae	*Psammomys*	XM_055601670.1	XM_055603568.1		XM_055603303.1	GCF_907164565.1*
*Meriones unguiculatus*	Muridae	*Meriones*	XM_021648049.2		GCF_030254825.1*	XM_021663462.2	GCF_030254825.1*
*Mus spicilegus*	Muridae	*Mus*				GCA_003336285.2*	

*Note:* The numbers with * and # mean the genome assembly number used for extracting gene sequences of target species in NCBI (https://www.ncbi.nlm.nih.gov/) and CNCB (https://www.cncb.ac.cn/), respectively.

For population‐level analysis, we extracted all five adaptor sequences from whole‐genome data of RT (33 individuals) and RN (50 individuals) populations from our previous studies (NGDC accessions: CRA001635 and CRA003158, respectively; Chen, Hou, et al. 2021; Chen, Zhao, et al. 2021) (Table S1). First, we performed single‐nucleotide polymorphism (SNP) calling using the genome analysis toolkit (GATK v4.1) HaplotypeCaller protocol (McKenna et al. 2010). Then, we obtained adaptor gene sequences with GATK FastaAlternateReferenceMaker protocol (McKenna et al. 2010) with default parameters.

### 2.2 Interspecific Selection Analysis

Following established methodologies (Su et al. 2024), we employed multiple approaches to assess selection pressures: First, we used the maximum likelihood (ML) framework to evaluate positive and negative selection at each adaptor during Muridae evolution, based on the rate per site of nonsynonymous substitution (dN) to the rate per site of synonymous substitutions (dS). We used the neighbor joining trees as the working topology for the adaptor genes. When performing gene selection pressure analysis using PAML (particularly the codeml program), it is generally recommended to employ gene trees rather than species trees. If the topology of the gene tree conflicts with that of the species tree, imposing the species tree may lead to erroneous branch assignments, thereby affecting the estimation of the *ω* ratio (dN/dS) and potentially generating false‐positive or false‐negative outcomes. Because genes can undergo evolutionary histories distinct from the species as a whole, such as through positive selection acting on specific lineages, using a gene tree enables more accurate detection of such signals. For these reasons, gene trees were used for the PAML analysis in this study. We tested the effect of positive selection on Muridae lineage by estimating global *ω* values under the M7/M8 branch model with CODEML in PAML v4 (Yang 1997; Jeffares et al. 2015; Álvarez‐Carretero et al. 2023). The M7 model restricts codon evolution to either neutral evolution or purifying selection (where dN/dS ≤ 1), while the M8 model incorporates an additional category of sites evolving under positive selection (dN/dS > 1). These two nested models were compared using a likelihood ratio test (LRT) with 2 degrees of freedom (Nielsen and Yang 1998; Yang et al. 2000). Positively selected sites under model M8 were identified using a Bayes empirical Bayes (BEBs) approach (Yang et al. 2005). Sites with a posterior probability exceeding 90% were considered candidate targets of positive selection, as pinpointing individual selected sites is statistically more challenging than detecting the presence of selection among a proportion of sites (Wlasiuk and Nachman 2010; Těšický et al. 2020). The one‐ratio model (model M0, assuming the same *ω* for all branches and sites, model = 0 and Nsites = 0) analysis was also performed to test the overall selection pressure in Muridae species.

Next, we used several alternative selection tests to evaluate the selection pressure occurring in our diverse data set with slightly different assumptions and constraints. To do this, a series of ML methods were implemented in the DATAMONKEY Web server (Weaver et al. 2018). The fixed‐effect likelihood (FEL) model estimates the ratio of nonsynonymous to synonymous substitution on a site‐by‐site basis without assuming an a priori distribution of rates across sites (Kosakovsky Pond and Frost 2005). The single likelihood ancestor counting (SLAC) model is based on the reconstruction of ancestral sequences and the counts of synonymous and nonsynonymous changes at each codon position in a phylogeny (Kosakovsky Pond and Frost 2005). The fast, unconstrained Bayesian approximation (FUBAR) model is based on random effect likelihood methods (Murrell et al. 2013). The mixed effects model of evolution (MEME) employs a mixed‐effects ML approach to test the hypothesis that individual sites have been subjected to episodic positive or diversifying selection (Murrell et al. 2012). We performed all these analyses with the significance level of posterior probability established by default to 0.9. Codons were considered robust candidates for positive selection when they were identified to be under positive selection by at least two ML methods.

### 2.3 Population Genetic Analysis

We used DnaSP v5 to estimate the nucleotide polymorphism, haplotype number, nucleotide diversity, haplotype diversity (Hd), and average number of nucleotide differences in the gene sequences of these five adaptors with default parameters (Librado and Rozas 2009). We use “haplotype” to denote unique coding‐sequence alleles for each adaptor gene. We calculated the average nucleotide diversity values of noncoding regions across the whole genome using vcftools (Danecek et al. 2011). We built phylogenetic trees using MEGA 6 via the neighbor‐joining (NJ) method (Tamura et al. 2013). Additionally, we constructed phylogenetic trees based on all variant sites of the genome using VCF2Dis v1.42 (https://github.com/BGI‐shenzhen/VCF2Dis) with default arguments. We used Recombination Detection Program 4 (RDP4, Martin et al. 2015) and GENECONV v1.81 (Sawyer 1989) under default parameters to determine whether gene conversion occurred among rat populations for these adaptor genes.

## 3 Results

### 3.1 Genetic Characteristics at the Interspecific Level

**FIGURE 1:**
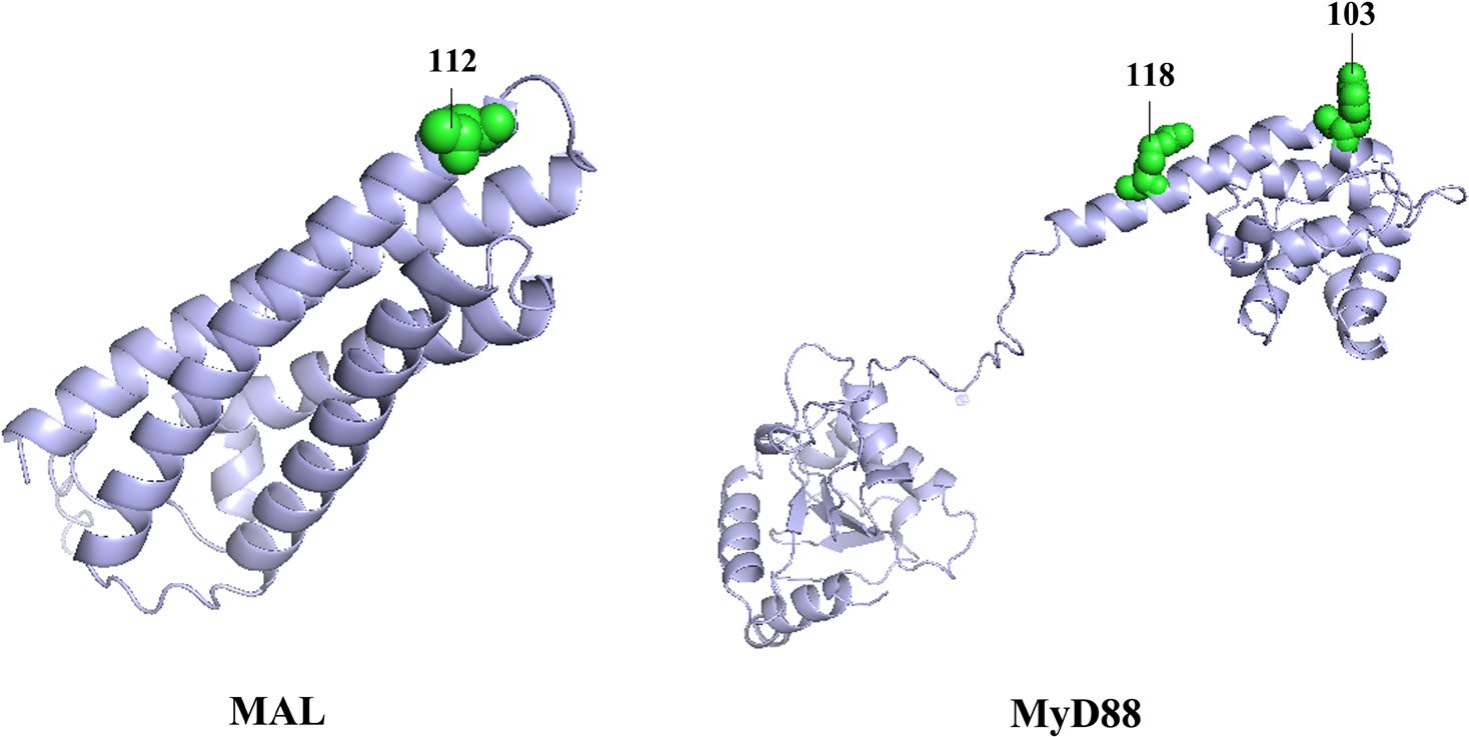
Positively selected sites visualized on the three‐dimensional structures of MAL and MyD88. The structure and amino acids refer to *Rattus norvegicus* .

**FIGURE 2:**
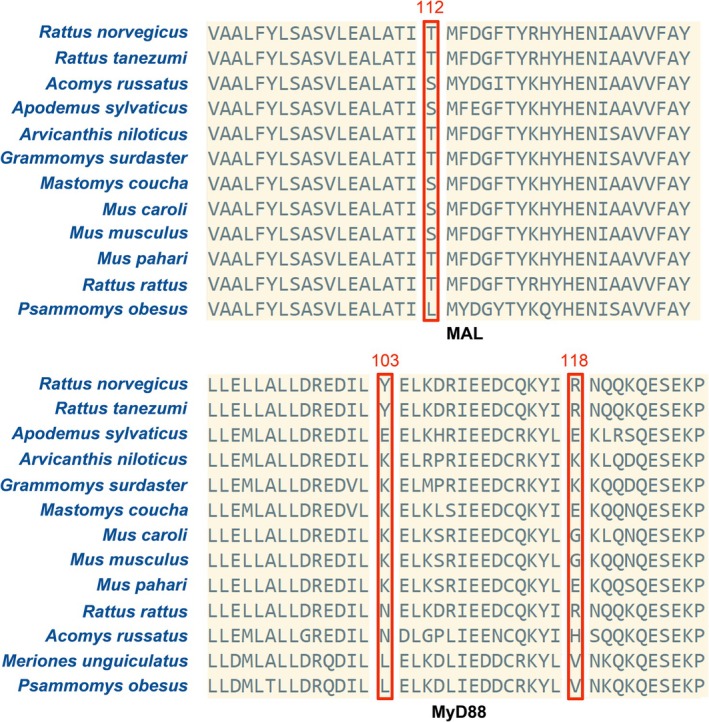
Details of mutations at positively selected amino acid sites (PSAs) among murid species. One PSA (codon 112) in MAL and two PSAs (codon 103 and 118) in MyD88.

Selection pressure analyses revealed that all five adaptor genes evolved under purifying selection (*ω* < 1; Table 3). Notably, we identified one (codon 112) and two (codon 103 and 118) positively selected amino acid sites in MAL and MyD88, respectively, which were consistently supported by multiple detection methods (Table 3; Figure 1). These three positively selected amino acid sites show high genetic diversity in murids (Figure 2).

**TABLE 3:** Selection analyses with codon‐based methods for five adaptors in murid rodents.

Gene	Number of species	Test of selection^a^					Sites under selection identified by different methods^b^				
		lnL M7	lnL M8	2lnΔL^c^	Significance	*ω* ^d^	PAML M8^e^	FEL^f^	SLAC^g^	FUBAR^h^	MEME
MAL	12	−1299.0858	−1294.02013	10.1313	**	0.1752	112			112	112
Myd88	13	−3054.5642	−3049.66584	9.7968	**	0.15148	39,103,107,118	49		103,120	12,68,94,118
TRAM1	14	−3067.8741	−3066.23595	3.276386	ns	0.04957				74	340
TRIF	12	−1233.0372	−1233.03861	−0.002732	ns	0.14962					
SARM1	13	−5762.1405	−2762.14184	−0.0027	ns	0.02577					388

Abbreviation: ns, not significant.

^a^ Selection analyses with codon‐based methods under the M7/M8 branch model.

^b^ Sites with underline were positively selected sites that were identified to be under positive selection by at least two ML methods. Site positions are relative to the protein sequence of *Rattus norvegicus* .

^c^ 2lnΔL is distributed approximately as *χ* ^2^ with 2 degrees of freedom.

^d^ Estimated dN/dS of the sites under selection in M0.

^e^ Codons with posterior probablilities > 90% in the BEB analyses.

^f^ Codons with *p* values < 0.1.

^g^ Codons with *p* values < 0.1.

^h^ Codons with *p* values < 0.1.

** *p* < 0.01.

### 3.2 Intraspecific Genetic Characteristics

Haplotype distributions differed significantly between RT and RN populations. The numbers of haplotypes of these five genes (MAL, MyD88, SARM1, TRAM1, and TRIF) in the RT population were 4, 7, 19, 5, and 2, respectively. For RN, the numbers of haplotypes of these five genes (MAL, MyD88, SARM1, TRAM1, and TRIF) were 3, 2, 7, 1, and 1, respectively. Both Hd and nucleotide diversity (π) were consistently higher in RT than RN for all genes (Table 4). Notably, the average nucleotide diversity values of the noncoding regions across the whole genome in the RT and RN populations were 0.00528 and 0.00088, respectively, both of which were greater than those of these five adaptor genes. The TRAM1 and TRIF genes showed complete conservation in RNs, and no mutation sites were detected. Moreover, we did not detect any significant signals of positive or negative selection in adaptor genes in either rat population (Table 5).

**TABLE 4:** Genetic characteristics for five adaptors in *Rattus tanezumi* and *R. norvegicus* populations.

Gene	Size (bp)	*N*		*S*		*H*		Hd		π		θW	
		RT	RN	RT	RN	RT	RN	RT	RN	RT	RN	RT	RN
MAL	462	33	50	2	2	4	3	0.72	0.536	0.00212	0.00085	0.00107	0.00097
MyD88	891	33	50	8	1	7	2	0.686	0.429	0.00329	0.00048	0.00221	0.00025
SARM1	2175	33	50	17	4	19	7	0.955	0.811	0.00291	0.00084	0.00193	0.00041
TRAM1	1125	33	50	3	0	5	1	0.708	0	0.00083	0	0.00066	0
TRIF	630	33	50	1	0	2	1	0.117	0	0.00019	0	0.00039	0

Abbreviations: *H*, number of haplotypes; Hd, haplotype diversity; *N*, number of sequences analyzed; RN, *R. norvegicus* ; RT, *Rattus tanezumi* ; *S*, single nucleotide polymorphism; θ_W_, Theta (per site) from *S*; π, nucleotide diversity (per site).

**TABLE 5:** Netrality tests for five adaptors in *Rattus tanezumi* and *R. norvegicus* populations.

Gene	Tajima's *D* (average value of *D*)		Fu‐Li D* (average value of D*)		Fu‐Li F* (average value of F*)		MK test (NI value)		HKA test (*X*‐squared value)	
	RT	RN	RT	RN	RT	RN	RT	RN	RT	RN
MAL	0.0036	−0.0491	0.0305	−0.0734	0.0065	−0.0721	1.2	0.001	1.731	0.001
MyD88	−0.1099	−0.0218	−0.0767	−0.0338	−0.0913	0.0174	0.883	0.266	0.749	1.23
SARM1	−0.1069	−0.0723	−0.0353	−0.0383	−0.0156	−0.0436	0.5	2.9	0.523	0.892
TRAM1	−0.0892	NA	−0.0273	NA	−0.0176	NA	0.001	NA	1.79	NA
TRIF	−0.0789	NA	−0.0973	NA	−0.0393	NA	0.001	NA	1.161	NA

*Note:* NA means could not calculate because there are no polymorphisms in the data. No significant signals of positive or negative selection were detected in any adaptor gene in either rat population.

**FIGURE 3:**
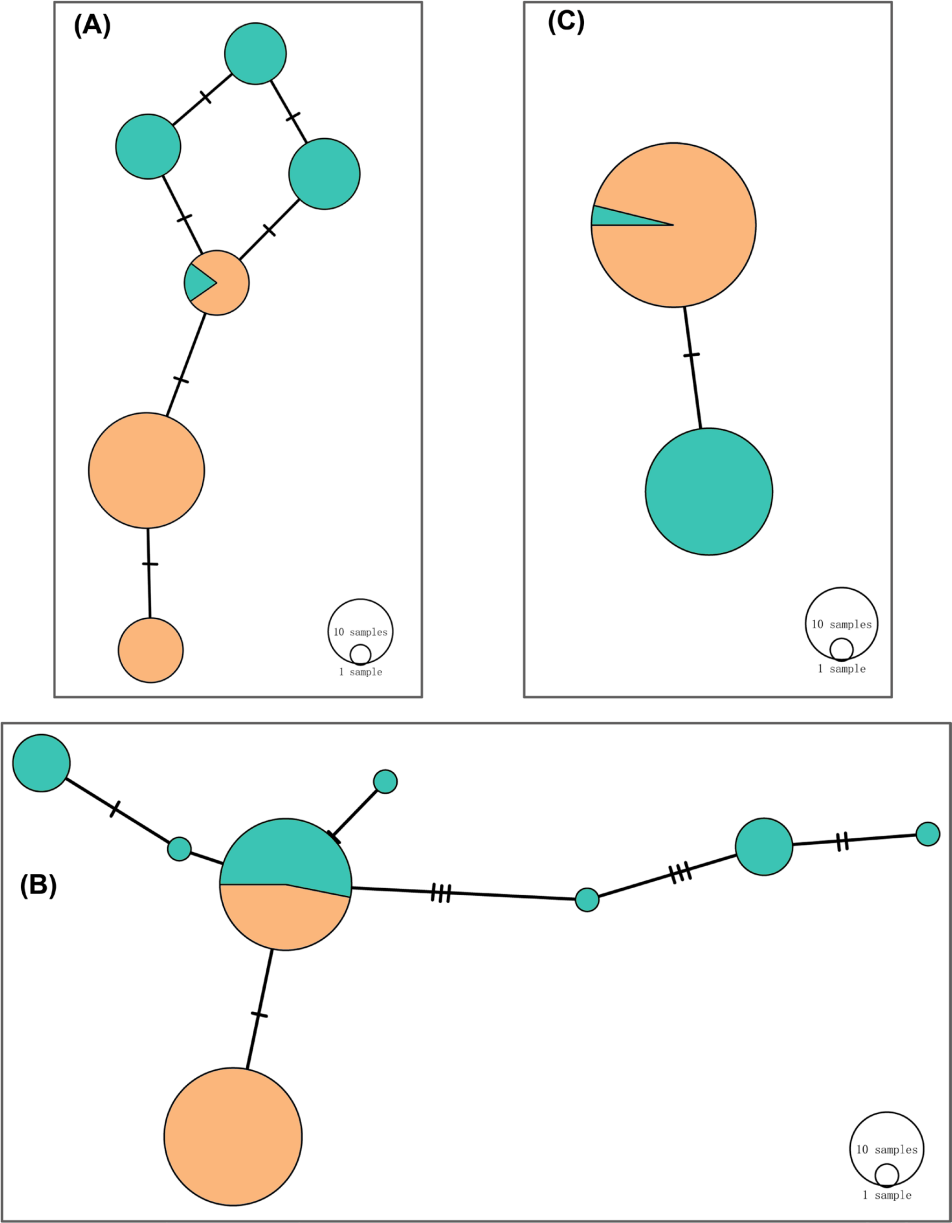
Haplotype networks of MAL (A), MyD88 (B), and TRIF (C). The haplotype network was constructed in 83 rats including RT and RN. Circle size reflected the number of rats with the respective haplotype. A dash on the joined line indicated one nucleotide variation. Yellow color represent RN and green color represent RT.

**FIGURE 4:**
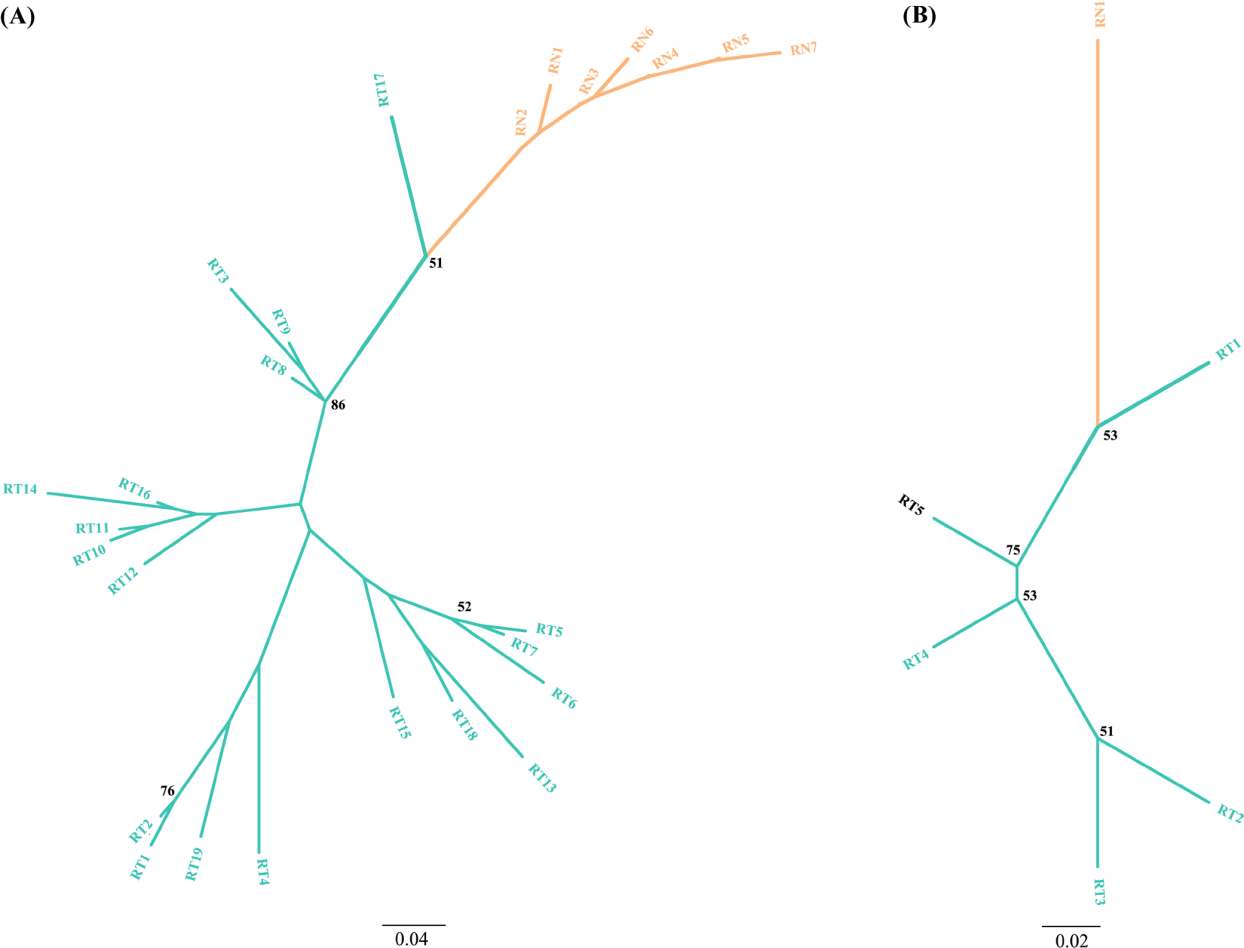
Neighbor‐joining tree of SARM1 (A) and TRAM1 (B) genes from RT and RN populations. Branch lengths were computed based on p‐distance method. Each tip corresponds to a unique haplotype. Clades with yellow color represent RN haplotypes, and clades with green color represent RT haplotypes. Bootstrap percentages (from 1000 replications) for major clusters are shown on internal branches. Bootstraps values > 50 are indicated at their respective nodes. For SARM1, Haplotype RT17 clustered together with RN alleles (poor bootstrap support between them: 51%). For TRAM1, Haplotype RT1 clustered together with the RN allele (poor bootstrap support between them: 53%).

**FIGURE 5:**
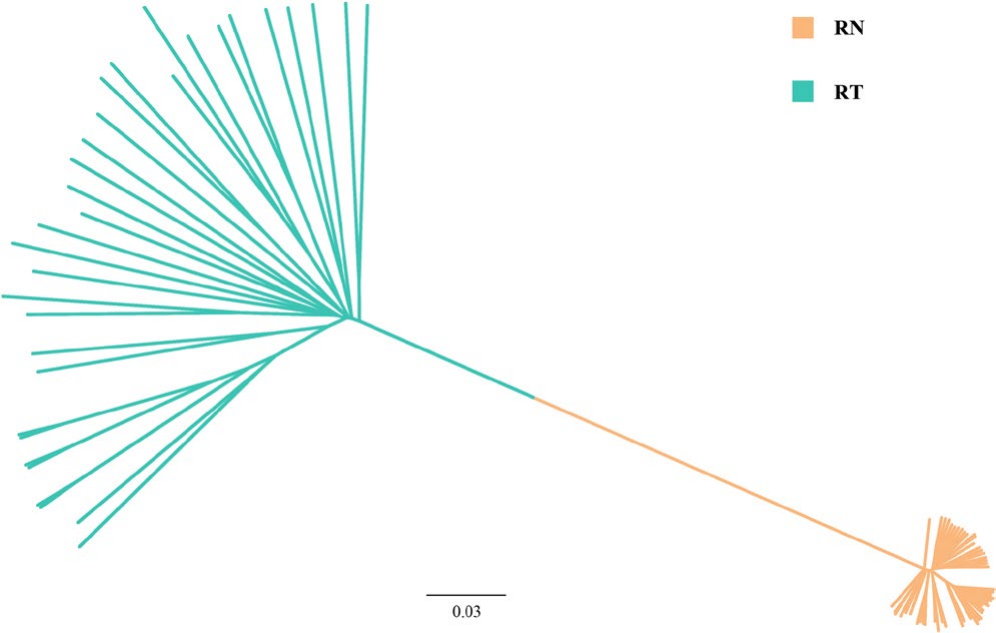
Neighbor‐joining tree based on all variant sites across the genome of RT and RN populations. Branch lengths were computed based on p‐distance method. Each tip corresponds to a unique haplotype. Clades with yellow color represent RN haplotypes, and clades with green color represent RT haplotypes.

Surprisingly, identical or highly similar genetic variants (> 99.8% sequence identity) were observed between these two rat populations for all five of these adaptors. For the MAL, MyD88, and TRIF genes, one haplotype shared between RT and RN was observed (Figure 3). For the SARM1 and TRAM1 genes, at least one type of RT haplotype clustered together with the RN haplotypes (Figure 4). Conversely, phylogenetic trees based on genome‐scale data separated individuals of RTs and RNs completely (Figure 5). Moreover, we did not detect any recombination event between these two rat populations in adaptor genes.

## 4 Discussion

TIR‐domain‐containing adaptor proteins play pivotal roles in vertebrate immunity, making the study of their evolutionary patterns essential for ecological and evolutionary research. Our study provided novel insights into the evolutionary dynamics of TIR‐domain‐containing adaptor proteins in murid rodents, a highly successful mammalian family with global distribution. The predominant purifying selection observed across all five adaptors (*ω* < 1) underscored their essential and conserved roles in TLR‐mediated immune responses (O'Neill and Bowie 2007). This evolutionary pattern aligned with previous findings in humans (Fornarino et al. 2011), suggesting strong functional constraints on these critical signaling molecules throughout mammalian evolution.

The identification of positively selected sites in MyD88 (positions 103 and 118) and MAL (position 112) Present particularly intriguing findings. The MyD88 variants occur in functionally critical domains: position 103 within the death domain (DD) that mediates myddosome assembly through IRAK interactions (Ve et al. 2017), and position 118 in the intermediary domain essential for proper protein conformation (Avbelj et al. 2011). Their location in key functional domains suggests potential adaptive significance, which needs to be validated experimentally in future studies. Previous studies have demonstrated that analogous mutations in these domains can significantly alter signaling outcomes (Loiarro et al. 2009; Ve et al. 2017). The positively selected site in MAL's TIR domain (position 112) may represent another adaptive hotspot, particularly given established evidence that TIR domain variants can dramatically impact TLR2/4 signaling (Nagpal et al. 2009). The known association between MAL polymorphisms and infectious disease resistance in humans (Khor et al. 2007) further supports the potential functional importance of this variation. These findings collectively suggest that while adaptor proteins are generally under strong purifying selection, certain residues may undergo positive selection to fine‐tune immune responses in specific ecological contexts. The remarkable conservation of these adaptors in murid rodents, particularly when compared to their neutral region diversity, highlights their fundamental importance in maintaining immune system integrity. This conservation is especially noteworthy given the exceptional adaptability of murid rodents to diverse environments and their role as reservoirs for numerous zoonotic pathogens (Wu et al. 2018; Su et al. 2022). The observed evolutionary patterns may reflect balancing selection pressures to maintain robust pathogen recognition while avoiding excessive immune activation. Specially, our findings raise several important questions for future research. First, experimental studies are needed to characterize the functional consequences of the identified variants. Second, comparative analyses across additional mammalian taxa could reveal whether these patterns represent rodent‐specific adaptations or more general evolutionary trends.

Our study revealed striking genetic similarity in five adaptor proteins between sympatric RT and RN populations. Such shared polymorphisms in closely related species typically arise through four evolutionary mechanisms: (1) recombination, (2) trans‐species polymorphism (TSP), (3) convergent evolution, or (4) incomplete lineage sorting (ILS) (Cho et al. 2006; Těšický and Vinkler 2015). Recombination was not detected between these two rat populations in adaptor genes. TSP is generally observed in highly polymorphic immune genes like MHC and TRIM5α, where balancing selection maintains ancestral variants across species (Cagliani et al. 2010; Azevedo et al. 2015; Million and Lively 2022). The adaptor genes showed low polymorphism in both RT and RN populations. However, TSPs may not require high diversity when they involve a small number of long‐lived alleles. Future study should be conducted with broader outgroup sampling to reconstruct the ancestral state of these rat species. Convergent evolution refers to the independent evolution of similar phenotypic features or functions among different taxa with distant genetic relationships, due to adaptation to similar environments or ecological niches, under the influence of natural selection. These features do not arise from a common direct ancestor, but from independent origins (McGhee 2011; Stayton 2015). ILS refers to the phenomenon of “gene tree and species tree inconsistency” that occurs at the genetic level in rapidly differentiated species in recent times. The reason is that during species differentiation, certain genes in the ancestral population exhibit polymorphism (multiple alleles), and subsequently different alleles are randomly fixed in different offspring lineages, resulting in a mismatch between the evolutionary relationship of certain genes and the true differentiation relationship of the species (Degnan and Rosenberg 2009). RT and RN arose from the same lineage (*Rattus*), and the divergence time between these two species is less than 3 million years (Robins et al. 2008). Moreover, no significant signals of recent positive or negative selection were detected in either rat population, indicating the weak natural selection acting on adaptor genes of these two rat populations. Therefore, compared to convergent evolution, TSP/ILS is more likely to be the cause of the similarity and shared haplotypes in adaptor genes between these two rat populations.

## 5 Conclusions

This study significantly advances our understanding of immune gene evolution in ecologically important species and provides a foundation for future investigations into the molecular mechanisms underlying pathogen–host interactions in natural populations. The combination of evolutionary conservation and targeted diversification at key residues suggests a sophisticated balance between maintaining core immune functions and allowing adaptive fine‐tuning in these critical signaling molecules.

## Author Contributions

**Qianqian Su:** data curation (equal), formal analysis (equal), funding acquisition (equal), investigation (equal), methodology (equal), project administration (equal), visualization (equal), writing – original draft (equal). **Zhenhua She:** methodology (equal). **Yi Chen:** conceptualization (equal), formal analysis (equal), methodology (equal), resources (equal), validation (equal), writing – review and editing (equal).

## Funding

This work was supported by Natural Science Foundation of Hunan, 2023JJ41038. Excellent Youth Project of Hunan Education Department, 22B0257. National Natural Science Foundation of China, 32400413.

## Conflicts of Interest

The authors declare no conflicts of interest.

## Supporting information


**Data S1:** ece372851‐sup‐0001‐DataS1.docx.

## Data Availability

The dataset of this study is provided as additional Supporting Information for the manuscript.

## References

[ece372851-bib-0001] Álvarez‐Carretero, S. , P. Kapli , and Z. Yang . 2023. “Beginner's Guide on the Use of PAML to Detect Positive Selection.” Molecular Biology and Evolution 40, no. 4: msad041. 37096789 10.1093/molbev/msad041 PMC10127084

[ece372851-bib-0002] Avbelj, M. , S. Horvat , and R. Jerala . 2011. “The Role of Intermediary Domain of MyD88 in Cell Activation and Therapeutic Inhibition of TLRs.” Journal of Immunology 187, no. 5: 2394–2404. 10.4049/jimmunol.1100515 21804016

[ece372851-bib-0003] Azevedo, L. , C. Serrano , A. Amorim , et al. 2015. “Trans‐Species Polymorphism in Humans and the Great Apes Is Generally Maintained by Balancing Selection That Modulates the Host Immune Response.” Human Genomics 9: 1–6. 26337052 10.1186/s40246-015-0043-1 PMC4559023

[ece372851-bib-0004] Baker, R. E. , A. S. Mahmud , I. F. Miller , et al. 2022. “Infectious Disease in an Era of Global Change.” Nature Reviews Microbiology 20, no. 4: 193–205. 34646006 10.1038/s41579-021-00639-z PMC8513385

[ece372851-bib-0005] Cagliani, R. , M. Fumagalli , M. Biasin , et al. 2010. “Long‐Term Balancing Selection Maintains Trans‐Specific Polymorphisms in the Human TRIM5 Gene.” Human Genetics 128: 577–588. 20811909 10.1007/s00439-010-0884-6

[ece372851-bib-0006] Carty, M. , R. Goodbody , M. Schröder , J. Stack , P. N. Moynagh , and A. G. Bowie . 2006. “The Human Adaptor SARM Negatively Regulates Adaptor Protein TRIF‐Dependent Toll‐Like Receptor Signaling.” Nature Immunology 7, no. 10: 1074–1081. 16964262 10.1038/ni1382

[ece372851-bib-0007] Chen, Y. , G. Hou , M. Jing , et al. 2021. “Genomic Analysis Unveils Mechanisms of Northward Invasion and Signatures of Plateau Adaptation in the Asian House Rat.” Molecular Ecology 30, no. 24: 6596–6610. 34564921 10.1111/mec.16194

[ece372851-bib-0008] Chen, Y. , L. Zhao , H. Teng , et al. 2021. “Population Genomics Reveal Rapid Genetic Differentiation in a Recently Invasive Population of *Rattus norvegicus* .” Frontiers in Zoology 18: 1–10. 33499890 10.1186/s12983-021-00387-z PMC7836188

[ece372851-bib-0009] Cho, S. , Z. Y. Huang , D. R. Green , D. R. Smith , and J. Zhang . 2006. “Evolution of the Complementary Sex‐Determination Gene of Honey Bees: Balancing Selection and Trans‐Species Polymorphisms.” Genome Research 16, no. 11: 1366–1375. 17065615 10.1101/gr.4695306 PMC1626638

[ece372851-bib-0010] Danecek, P. , A. Auton , G. Abecasis , et al. 2011. “The Variant Call Format and VCFtools.” Bioinformatics 27, no. 15: 2156–2158. 21653522 10.1093/bioinformatics/btr330 PMC3137218

[ece372851-bib-0011] Degnan, J. H. , and N. A. Rosenberg . 2009. “Gene Tree Discordance, Phylogenetic Inference and the Multispecies Coalescent.” Trends in Ecology & Evolution 24, no. 6: 332–340. 19307040 10.1016/j.tree.2009.01.009

[ece372851-bib-0012] Fitzgerald, K. A. , and J. C. Kagan . 2020. “Toll‐Like Receptors and the Control of Immunity.” Cell 180: 1044–1066. 32164908 10.1016/j.cell.2020.02.041 PMC9358771

[ece372851-bib-0013] Fornarino, S. , G. Laval , L. B. Barreiro , J. Manry , E. Vasseur , and L. Quintana‐Murci . 2011. “Evolution of the TIR Domain‐Containing Adaptors in Humans: Swinging Between Constraint and Adaptation.” Molecular Biology and Evolution 28, no. 11: 3087–3097. 21659570 10.1093/molbev/msr137

[ece372851-bib-0014] Horng, T. , G. M. Barton , and R. Medzhitov . 2001. “TIRAP: An Adapter Molecule in the Toll Signaling Pathway.” Nature Immunology 2, no. 9: 835–841. 11526399 10.1038/ni0901-835

[ece372851-bib-0015] Jeffares, D. C. , B. Tomiczek , V. Sojo , et al. 2015. “A Beginners Guide to Estimating the Non‐Synonymous to Synonymous Rate Ratio of All Protein‐Coding Genes in a Genome.” In Parasite Genomics Protocols, edited by C. Peacock , vol. 1201. Humana Press. Methods in Molecular Biology. 10.1007/978-1-4939-1438-8_4 25388108

[ece372851-bib-0016] Khor, C. C. , S. J. Chapman , F. O. Vannberg , et al. 2007. “A Mal Functional Variant Is Associated With Protection Against Invasive Pneumococcal Disease, Bacteremia, Malaria and Tuberculosis.” Nature Genetics 39, no. 4: 523–528. 17322885 10.1038/ng1976 PMC2660299

[ece372851-bib-0017] Kosakovsky Pond, S. L. , and S. D. W. Frost . 2005. “Not So Different After All: A Comparison of Methods for Detecting Amino Acid Sites Under Selection.” Molecular Biology and Evolution 22, no. 5: 1208–1222. 15703242 10.1093/molbev/msi105

[ece372851-bib-0018] Librado, P. , and J. Rozas . 2009. “DnaSP v5: A Software for Comprehensive Analysis of DNA Polymorphism Data.” Bioinformatics 25, no. 11: 1451–1452. 19346325 10.1093/bioinformatics/btp187

[ece372851-bib-0053] Loiarro, M. , G. Gallo , N. Fantò , et al. 2009. “Identification of critical residues of the MyD88 death domain involved in the recruitment of downstream kinases.” Journal of Biological Chemistry 284, no. 41: 28093–28103. 19679662 10.1074/jbc.M109.004465 PMC2788860

[ece372851-bib-0020] Martin, D. P. , B. Murrell , M. Golden , A. Khoosal , and B. Muhire . 2015. “RDP4: Detection and Analysis of Recombination Patterns in Virus Genomes.” Virus Evolution 1: vev003. 27774277 10.1093/ve/vev003 PMC5014473

[ece372851-bib-0021] McGhee, G. R. 2011. Convergent Evolution: Limited Forms Most Beautiful. MIT Press.

[ece372851-bib-0022] McKenna, A. , M. Hanna , E. Banks , et al. 2010. “The Genome Analysis Toolkit: A MapReduce Framework for Analyzing Next‐Generation DNA Sequencing Data.” Genome Research 20, no. 9: 1297–1303. 20644199 10.1101/gr.107524.110 PMC2928508

[ece372851-bib-0023] Million, K. M. , and C. M. Lively . 2022. “Trans‐Specific Polymorphism and the Convergent Evolution of Supertypes in Major Histocompatibility Complex Class II Genes in Darters (Etheostoma).” Ecology and Evolution 12, no. 1: e8485. 36311547 10.1002/ece3.8485 PMC9601779

[ece372851-bib-0024] Murrell, B. , S. Moola , A. Mabona , et al. 2013. “FUBAR: A Fast, Unconstrained Bayesian Approximation for Inferring Selection.” Molecular Biology and Evolution 30, no. 5: 1196–1205. 23420840 10.1093/molbev/mst030 PMC3670733

[ece372851-bib-0025] Murrell, B. , J. O. Wertheim , S. Moola , T. Weighill , K. Scheffler , and S. L. Kosakovsky Pond . 2012. “Detecting Individual Sites Subject to Episodic Diversifying Selection.” PLoS Genetics 8, no. 7: e1002764. 22807683 10.1371/journal.pgen.1002764 PMC3395634

[ece372851-bib-0026] Musser, G. G. , and M. D. Carleton . 2005. “Superfamily Muroidea.” In Mammal Species of the World a Taxonomic and Geographic Reference, edited by D. E. Wilson and D. M. Reeder , 894–1531. Johns Hopkins University Press.

[ece372851-bib-0027] Nagpal, K. , T. S. Plantinga , J. Wong , et al. 2009. “A TIR Domain Variant of MyD88 Adapter‐Like (Mal)/TIRAP Results in Loss of MyD88 Binding and Reduced TLR2/TLR4 Signaling.” Journal of Biological Chemistry 284, no. 38: 25742–25748. 19509286 10.1074/jbc.M109.014886 PMC2757976

[ece372851-bib-0028] Nielsen, R. , and Z. Yang . 1998. “Likelihood Models for Detecting Positively Selected Amino Acid Sites and Applications to the HIV‐1 Envelope Gene.” Genetics 148, no. 3: 929–936. 9539414 10.1093/genetics/148.3.929 PMC1460041

[ece372851-bib-0029] O'Neill, L. A. J. , and A. G. Bowie . 2007. “The Family of Five: TIR‐Domain‐Containing Adaptors in Toll‐Like Receptor Signalling.” Nature Reviews Immunology 7, no. 5: 353–364. 10.1038/nri2079 17457343

[ece372851-bib-0030] O'Neill, L. A. J. , K. A. Fitzgerald , and A. G. Bowie . 2003. “The Toll–IL‐1 Receptor Adaptor Family Grows to Five Members.” Trends in Immunology 24, no. 6: 286–289. 12810098 10.1016/s1471-4906(03)00115-7

[ece372851-bib-0031] Puckett, E. E. , J. Park , M. Combs , et al. 2016. “Global Population Divergence and Admixture of the Brown Rat (*Rattus norvegicus*).” Proceedings of the Royal Society B: Biological Sciences 283, no. 1841: 20161762. 10.1098/rspb.2016.1762 PMC5095384 27798305

[ece372851-bib-0032] Robins, J. H. , P. A. McLenachan , M. J. Phillips , L. Craig , H. A. Ross , and E. Matisoo‐Smith . 2008. “Dating of Divergences Within the *Rattus* Genus Phylogeny Using Whole Mitochondrial Genomes.” Molecular Phylogenetics and Evolution 49: 460–466. 18725306 10.1016/j.ympev.2008.08.001

[ece372851-bib-0033] Sawyer, S. 1989. “Statistical Tests for Detecting Gene Conversion.” Molecular Biology and Evolution 6: 526–538. 2677599 10.1093/oxfordjournals.molbev.a040567

[ece372851-bib-0034] Siddle, K. J. , and L. Quintana‐Murci . 2014. “The Red Queen's Long Race: Human Adaptation to Pathogen Pressure.” Current Opinion in Genetics & Development 29: 31–38. 25170983 10.1016/j.gde.2014.07.004

[ece372851-bib-0035] Stayton, C. T. 2015. “What Does Convergent Evolution Mean? The Interpretation of Convergence and Its Implications in the Search for Limits to Evolution.” Interface Focus 5, no. 6: 20150039. 26640646 10.1098/rsfs.2015.0039 PMC4633856

[ece372851-bib-0036] Su, Q. , Y. Chen , and H. He . 2024. “Molecular Evolution of Toll‐Like Receptors in Rodents.” Integrative Zoology 19, no. 3: 371–386. 37403417 10.1111/1749-4877.12746

[ece372851-bib-0037] Su, Q. , Y. Chen , B. Wang , Q. Zhang , and H. He . 2022. “Genetic Characterizations of Toll‐Like Receptors in the Brown Rat and Their Associations With Pathogen Infections.” Integrative Zoology 17, no. 5: 879–889. 34003606 10.1111/1749-4877.12555

[ece372851-bib-0038] Takeuchi, O. , K. Takeda , K. Hoshino , O. Adachi , T. Ogawa , and S. Akira . 2000. “Cellular Responses to Bacterial Cell Wall Components Are Mediated Through MyD88‐Dependent Signaling Cascades.” International Immunology 12, no. 1: 113–117. 10607756 10.1093/intimm/12.1.113

[ece372851-bib-0039] Tamura, K. , G. Stecher , D. Peterson , A. Filipski , and S. Kumar . 2013. “MEGA6: Molecular Evolutionary Genetics Analysis Version 6.0.” Molecular Biology and Evolution 30, no. 12: 2725–2729. 24132122 10.1093/molbev/mst197 PMC3840312

[ece372851-bib-0040] Těšický, M. , H. Velová , M. Novotný , J. Kreisinger , V. Beneš , and M. Vinkler . 2020. “Positive Selection and Convergent Evolution Shape Molecular Phenotypic Traits of Innate Immunity Receptors in Tits (Paridae).” Molecular Ecology 29, no. 16: 3056–3070. 32652716 10.1111/mec.15547

[ece372851-bib-0041] Těšický, M. , and M. Vinkler . 2015. “Trans‐Species Polymorphism in Immune Genes: General Pattern or MHC‐Restricted Phenomenon?” Journal of Immunology Research 2015, no. 1: 838035. 26090501 10.1155/2015/838035 PMC4458282

[ece372851-bib-0042] Ve, T. , P. R. Vajjhala , A. Hedger , et al. 2017. “Structural Basis of TIR‐Domain‐Assembly Formation in MAL‐and MyD88‐Dependent TLR4 Signaling.” Nature Structural & Molecular Biology 24, no. 9: 743–751. 10.1038/nsmb.3444 PMC8059215 28759049

[ece372851-bib-0043] Vijay, K. 2018. “Toll‐Like Receptors in Immunity and Inflammatory Diseases: Past, Present, and Future.” International Immunopharmacology 59: 391–412. 29730580 10.1016/j.intimp.2018.03.002 PMC7106078

[ece372851-bib-0044] Weaver, S. , S. D. Shank , S. J. Spielman , M. Li , S. V. Muse , and S. L. Kosakovsky Pond . 2018. “Datamonkey 2.0: A Modern Web Application for Characterizing Selective and Other Evolutionary Processes.” Molecular Biology and Evolution 35, no. 3: 773–777. 29301006 10.1093/molbev/msx335 PMC5850112

[ece372851-bib-0045] Wlasiuk, G. , and M. W. Nachman . 2010. “Adaptation and Constraint at Toll‐Like Receptors in Primates.” Molecular Biology and Evolution 27: 2172–2176. 20410160 10.1093/molbev/msq104 PMC3107592

[ece372851-bib-0046] Wu, Z. , L. Lu , J. Du , et al. 2018. “Comparative Analysis of Rodent and Small Mammal Viromes to Better Understand the Wildlife Origin of Emerging Infectious Diseases.” Microbiome 6: 1–14. 30285857 10.1186/s40168-018-0554-9 PMC6171170

[ece372851-bib-0047] Yamamoto, M. , S. Sato , H. Hemmi , et al. 2003. “TRAM Is Specifically Involved in the Toll‐Like Receptor 4‐Mediated MyD88‐Independent Signaling Pathway.” Nature Immunology 4, no. 11: 1144–1150. 14556004 10.1038/ni986

[ece372851-bib-0048] Yammoto, M. , S. Sato , H. Hemmi , et al. 2003. “Role of Adaptor TRIF in the MyD88‐Independent Toll‐Like Receptor Signaling Pathway.” Science 301, no. 5633: 640–643. 12855817 10.1126/science.1087262

[ece372851-bib-0049] Yang, Z. 1997. “PAML: A Program Package for Phylogenetic Analysis by Maximum Likelihood.” Computer Applications in the Biosciences 13, no. 5: 555–556. 9367129 10.1093/bioinformatics/13.5.555

[ece372851-bib-0051] Yang, Z. , R. Nielsen , N. Goldman , and A. M. K. Pedersen . 2000. “Codon‐Substitution Models for Heterogeneous Selection Pressure at Amino Acid Sites.” Genetics 155, no. 1: 431–449. 10790415 10.1093/genetics/155.1.431 PMC1461088

[ece372851-bib-0052] Yang, Z. , W. S. W. Wong , and R. Nielsen . 2005. “Bayes Empirical Bayes Inference of Amino Acid Sites Under Positive Selection.” Molecular Biology and Evolution 22, no. 4: 1107–1118. 15689528 10.1093/molbev/msi097

