# Supplementary material for: New Insights Into the Evolution of Immune Adaptors in Murid Rodents
Source: Ecol Evol. 2026 Jan 5;16(1):e72851. doi: 10.1002/ece3.72851 (PMC12771607; doi:10.1002/ece3.72851)
Supplement: Supplementary file 1 — Data S1: ece372851‐sup‐0001‐DataS1.docx. [file ECE3-16-e72851-s001.docx]

Supplementary Data 1. Aligned sequences of MAL

>Rattus-norvegicus

ATGGCCCCGGCAGCAGCTTCGGGTGGCAGCACCCTGCCCAGTGGCTTCTCCGTCTTCGTCACCTTCCCTGACTTGCTCTTCATCTTTGAGTTTATCTTTGGAGGCCTGGTGTGGATCCTGATCGCCTCCTCCCTGGTACCCATGCCCCTGGTCCAGGGCTGGGTGATGTTTGTGTCTGTGTTCTGCTTCCTGGCCACCACTTCCCTGATGGTCATGTACATAATTGGTACTCATGGTGGGGAGACTTCCTGGATCACACTGGATGCAGCCTACCACTGTGTGGCTGCCCTGTTTTACCTCAGTGCCTCAGTCCTGGAAGCCCTGGCCACCATCACAATGTTCGATGGCTTTACCTACAGGCATTACCATGAGAACATCGCTGCAGTGGTGTTCGCCTATGTGGCTACTCTGCTCTACGTGATCCATGCTGTGTTTTCCTTAATCAGGTGGAAGTCTTCATAG

>Rattus-tanezumi

ATGGCCCCGGCAGCAGCTTCGGGTGGCAGCACCCTGCCCAGTGGCTTCTCCGTCTTCGTCACCTTCCCTGACTTGCTCTTCATCTTTGAGTTTATCTTTGGAGGCCTGGTGTGGATCCTGATCGCCTCCTCCCTGGTACCCATGCCCCTGGTCCAGGGTTGGGTGATGTTTGTGTCTGTGTTCTGCTTCCTGGCCACCACTTCCCTGATGGTCATGTACATAATTGGTACTCATGGTGGGGAGACTTCCTGGATCACACTGGATGCAGCCTACCACTGTGTGGCTGCCCTGTTTTACCTCAGTGCCTCAGTCCTGGAAGCCCTGGCCACCATCACAATGTTCGATGGCTTTACCTACAGGCATTACCATGAGAACATCGCTGCAGTGGTGTTCGCCTATGTGGCTACTCTGCTCTACGTGATCCATGCTGTGTTTTCCTTAATCAGGTGGAAGTCTTCATAG

>Acomys-russatus

ATGGCCCCCGCAGCGGCTTCGGGTGGCAGCACCCTGCCCAGTGGCTTCTCCGTCTTCGTCACCTTCCCTGACTTGCTCTTCGTCTTTGAGTTTATCTTTGGGGGCCTGGTATGGATCCTGATCGCCTCCTCCCTGGTGCCCTTTCCCCTGATCCAGGGCTGGGTGATGTTCGTGTCTGTGTTCTGCTTCGTGGCCACCACGTCCCTGATGATCATGTATGTCATTGGTACTCACGGTGGTGAGACTTCCTGGATCACACTGGATGCAGCCTACCACTGTGTGGCTGCCCTATTTTACCTCAGTGCCTCAGTTCTGGAAGCCCTGGCCACCATCTCAATGTATGATGGCATCACCTACAAGCATTACCATGAAAACATCGCTGCAGTGGTGTTTGCCTACGTGGCCACGGTGCTCTATGTGGTCCACGCCGTGTTCTCCTTAATCAGATGGAAGTCTTCATAG

>Apodemus-sylvaticus

ATGGCCCCGGCAGCGGCTTCGGGTGGCAGCACCCTGCCCAGTGGCTTGTCGGTCTTCGCCACCTTCCCTGACTTGCTCTTCGTTTGTGAGTTTATCTTCGGAGGCCTGGTATGGATCCTGATTGCCTCCTCCCTAGTACCCCTGCCCCTGATCCAGGGCTGGGTGATGTTTGTGTCCGTGTTCTGCTTCGTGGCCACCACTTCCCTGATGGTCTTGTACATAATTGGTTCTCATGGCGGTGAGACTTCCTGGATCACACTGGATGCAGCCTACCACTGTGTGGCTGCCCTATTTTACCTCAGTGCCTCAGTTCTGGAAGCCCTGGCCACCATCTCAATGTTTGAAGGCTTTACCTACAAGCATTACCATGAAAACATCGCCGCAGTGGTGTTTGCCTATGTGGTCACTCTGCTCTACGTGGTGCATGCTGTGTTTTCCTTAATCAGATGGAAGTCTTCATAG

>Arvicanthis-niloticus

ATGGCCCCGGCAGCGGCTTCGGGTGGCAGCACCCTGCCCAGTGGCTTCTCGGTCTTCGTCACCTTTCCTGACTTGCTCTTCGTCTTGGAGTTTATCTTTGGAGGCCTGGTATGGATCCTGATTGCCTCCTCCCTGGTACCCTTGCCCCTGATCCAGGGCTGGGTGATGTTCGTGTCTGTGTTCTGCTTCGTGGCCACCACTTCCCTGATGGTCATGTACATAATTGGTACTCATGGAGGGGAGGCTTCCTGGATCACACTGGATGCAGCCTACCACTGTGTGGCTGCCCTATTTTACCTCAGTGCCTCAGTTCTGGAAGCCCTGGCCACCATCACGATGTTCGATGGCTTTACCTACAAGCATTACCATGAAAACATCTCTGCAGTGGTGTTTGCCTATGTGGCTACTCTGCTCTATGTGGTGCATGCTGTGTTTTCCTTAATCAGATGGAAGTCTTCATAG

>Grammomys-surdaster

ATGGCCCCAGCAGCGGCTTCGGGTGGCAGTACCCTGCCCAGTGGTTTCTCGGTCTTCGTCACCTTCCCTGACTTGCTCTTCGTCTTAGAGTTCATCTTTGGAGGCCTGGTGTGGATCCTGATCGCCTCCTCCCTGGTGCCCTTGCCCCTGATCCAGGGCTGGGTGATGTTCGTGTCGGTGTTCTGCTTTGTGGCCACCACTTCCCTGATGGTCATGTACATCATTGGTACTCATGGCGGGGAGGCTTCCTGGATCACACTGGATGCAGCCTACCACTGTGTGGCTGCCCTGTTTTACCTCAGTGCCTCAGTTCTGGAAGCCCTGGCCACCATCACAATGTTCGATGGCTTTACCTACAAGCATTACCATGAGAACATCTCCGCAGTGGTGTTTGCCTATGTGGCTACTCTGCTCTACGTGGTGCACGCTGTGTTTTCCTTAATCAGATGGAAGTCTTCATAG

>Mastomys-coucha

ATGGCCCCGGCAGCGGCTTCGGGTGGCAGCACCCTGCCCAGTGGCTTCTCGGTCTTCACCACCTTCCCTGACTTGCTCTTCGTTTGTGAGTTTATCTTTGGAGGCCTGGTATGGATCCTGATTGCCTCTTCCCTGGTACCCTTACCCCTGGTCCAGGGCTGGGTGATGTTCGTGTCCGTGTTCTGTTTTGTGGCCACCACTTCCCTGATGGTCTTGTACATAATTGGTACTCATGGCGGTGAGACTTCCTGGATCACATTGGATGCAGCCTACCACTGTGTGGCTGCCCTATTTTACCTCAGTGCCTCAGTTCTGGAAGCCCTGGCCACCATCAGCATGTTCGATGGCTTTACCTACAAGCATTACCATGAAAACATTGCGGCAGTGGTGTTTGCCTACGTGGTCACTCTGTTCTACGTGGTCCATGCTGTGTTTTCCTTAATCAGATGGAAGTCTTCATAG

>Mus-caroli

ATGGCTCCGGCAGCGGCTTCGGGTGGCAGCACCCTGCCCAGTGGCTTCTCGGTCTTCACCACCTTCCCTGACTTGCTCTTCGTTTGTGAGTTTGTCTTTGGAGGCCTGGTGTGGATCCTGATTGCCTCCTCCCTGGTACCCTTGCCCCTGGCCCAGGGCTGGGTGATGTTTGTGTCTGTGTTCTGCTTTGTGGCCACCACTACCCTGATGATCTTGTACATAATTGGTACTCATGGCGGTGAGACTTCCTGGATCACACTGGATGCAGCCTACCACTGTGTGGCTGCCCTATTTTACCTCAGTGCCTCAGTTCTGGAAGCCCTGGCCACCATCTCAATGTTTGATGGCTTTACCTACAAGCATTACCATGAAAACATCGCCGCAGTGGTGTTTGCCTACGTGGTCACTCTGATCTACGTGGTCCATGCTGTGTATTCCTTAATCAGATGGAAGTCTTCATAG

>Mus-musculus

ATGGCTCCGGCAGCGGCTTCGGGTGGCAGCACCCTGCCCAGTGGCTTCTCGGTCTTCACCACCTTCCCTGACTTGCTCTTCGTTTGTGAGTTTGTCTTTGGAGGCCTGGTGTGGATCCTGATTGCCTCCTCCCTGGTACCCTTGCCCCTGGCCCAGGGCTGGGTGATGTTTGTGTCTGTGTTCTGCTTTGTGGCCACCACTTCCCTGATGATCTTGTACATAATTGGTACTCATGGCGGTGAGACTTCCTGGATCACACTGGATGCAGCCTACCACTGTGTGGCTGCCCTATTTTACCTCAGTGCCTCAGTTCTGGAAGCCCTGGCCACCATCTCAATGTTTGATGGCTTTACTTACAAGCATTACCATGAAAACATCGCCGCAGTGGTGTTTGCCTACGTGGTCACTCTGATCTACGTGGTCCATGCTGTGTTTTCCTTAATCAGATGGAAGTCTTCATAG

>Mus-pahari

ATGGCTCCAGCAGCGGCTTCGGGTGGCAGCACCCTGCCCAGTGGCTTCTCGGTCTTCACCACCTTCCCTGACTTGCTCTTCGTTTGTGAGTTTATCTTTGGAGGCCTGGTGTGGATCCTGATTGCCTCCTCTCTGGTACCCTTGCCCCTGGCCCAGGGCTGGGTGATGTTTGTGTCTGTGTTCTGCTTTGTGGCCACCACTTCCCTGATGGTCTTGTACATAATTGGTACTCACGGCGGTGAGACTTCCTGGATCACACTGGATGCAGCCTACCACTGTGTGGCTGCCCTATTTTACCTCAGTGCCTCAGTTCTGGAAGCCCTGGCCACCATCACAATGTTCGATGGCTTTACCTACAAGCATTACCATGAAAACATCGCCGCAGTGGTGTTTGCCTATGTGGTCACTCTGATCTACGTGGTCCACGCTGTGTTTTCCTTAATCAGATGGAAGTCTTCATAG

>Rattus-rattus

ATGGCCCCGGCAGCAGCTTCGGGTGGCAGCACCCTGCCCAGTGGCTTCTCCGTCTTCGTCACCTTCCCTGACTTGCTCTTCATCTTTGAGTTTATCTTTGGAGGCCTGGTGTGGATCCTGATCGCCTCCTCCCTGGTACCCATGCCCCTGGTCCAGGGCTGGGTGATGTTTGTGTCTGTGTTCTGCTTCCTGGCCACCACTTCCCTGATGGTCATGTACATAATTGGTACTCATGGTGGGGAGACTTCCTGGATCACACTGGATGCAGCCTACCACTGTGTGGCCGCCCTGTTTTACCTCAGTGCCTCAGTCCTGGAAGCCCTGGCCACCATCACAATGTTCGATGGCTTTACCTACAGGCATTACCATGAGAACATCGCTGCAGTGGTGTTTGCCTATGTGGCTACTCTGCTGTACGTGATCCATGCTGTGTTTTCCTTAATCAGATGGAAGTCTTCATAG

>Psammomys_obesus

ATGGCCCCGGCAGCGGCTTCGGGTGGCAGCACCCTGCCCAGTGGCTTCTCGGTCTTCGTCACCTTCCCCGACTTGCTCTTCATCTTTGAGTTCATCTTTGGAGGCCTGGTGTGGATCCTGATCGCCTCCTCCCTAGTGCCCATGCCCCTGATCCAGGGCTGGGTGATGTTCGTGTCTGTGTTCTGCTTCGTGGTCACCACTGCCCTGATGGTCATGTACATAATTGGTACTCACGGCGGTGAGACTTCCTGGATCACACTGGATGCAGCCTATCACTGCGTGGCTGCCCTGTTTTACCTCAGTGCCTCAGTTCTGGAAGCCCTGGCCACCATCTTAATGTACGATGGCTATACCTACAAGCAGTACCATGAAAACATCTCCGCAGTGGTGTTTGCCTACGTGGCCACTCTGCTCTACGTGGTCCATGCCGTGTTCTCCTTAATCAGGTGGAAGTCTTCATAG

Supplementary Data 2. Aligned sequences of MyD88

>Rattus norvegicus

ATGTCTGCGGGAGGCCCCCGCGTGGGATCCGTGTCCGTGGACTCCTACTTGTTCTCTCTACCGTTGGTCGCGCTCAACGTGGGAGTGAGGCGCCGCCTCTCGCTGTTCTTGAACCCGCGGACAACCGCGGCGGCCGACTGGACCTCGCTGGCGGAGGAGATGGGTTTCGAGTACTTGGAGATCCGCGAGTTTGAGACGCGCCCCGATCCCACTCGCAGTTTGTTGGATGCCTGGCAGGGGCGCTCTGGCTCGTCGGTCGGCAGGCTGCTAGAGTTGCTAGCCTTGTTAGACCGTGAGGATATACTGTATGAACTGAAGGACCGCATCGAGGAGGACTGCCAGAAATACATACGCAACCAGCAGAAACAGGAGTCTGAGAAGCCCTTGCAGGTGGCCAGAGTGGAGAGCAGTGTCCCACAGACAAAGGAACTGGGAGGCATCACCACCCTCGATGACCCCCTGGGGCAAACGCCGGAGCTTTTCGACGCCTTCATCTGCTACTGCCCCAGTGATATTGAGTTTGTGCAGGAGATGATCCGGCAACTAGAACAGACAGACTATCGGCTTAAATTGTGTGTGTCCGACCGCGATGTCCTGCCTGGCACCTGCGTGTGGTCCATCGCCAGCGAGCTCATTGAGAAAAGGTGTCGTCGCATGGTGGTGGTTGTTTCTGACGATTACCTGCAAAGTAAGGAATGTGACTTCCAGACCAAGTTTGCTCTCAGCCTGTCTCCAGGTGTCCAACAGAAGCGACTGATCCCTATCAAGTACAAAGCAATGAAGAAGGACTTTCCTAGTATCCTACGGTTCATCACTATCTGCGACTATACCAACCCTTGCACCAAGTCCTGGTTCTGGACCCGTCTTGCCAAGGCTCTGTCCCTGCCCTGA

>Rattus tanezumi

ATGTCTGCGGGAGGCCCCCGCGTGGGATCCGTGTCCGTGGACTCCTACTTGTTCTCTCTACCGTTGGTCGCGCTCAACGTGGGAGTGAGGCGCCGCCTCTCGCTGTTCTTGAACCCGCGGACAACCGCGGCGGCCGACTGGACCTCGCTGGCGGAGGAGATGGGTTTCGAGTACTTGGAGATCCGCGAGTTTGAGTCGCGCCGCGATCCCACTCGCAGTTTGTTGGATGCCTGGCAGGGGCGCTCTGGCTCGTCGGTCGGCAGGCTGCTAGAGTTGCTAGCCTTGTTAGACCGTGAGGATATACTGTATGAACTGAAGGACCGCATCGAGGAGGACTGCCAGAAATACATACGCAACCAGCAGAAACAGGAGTCTGAGAAGCCCTTGCAGGTGGCCAGAGTGGAGAGCAGTGTCCCACAGACAAAGGAACTGGGAGGCATCACCACCCTCGATGACCCCCTGGGGCAAACGCCGGAGCTTTTCGATGCCTTCATCTGCTACTGCCCCAGTGATATTGAGTTTGTGCAGGAAATGATCCGGCAACTAGAACAGACAGACTATCGGCTTAAATTGTGTGTGTCCGACCGCGATGTCCTACCTGGCACCTGCGTGTGGTCCATCGCCAGCGAGCTCATTGAGAAAAGGTGTCGTCGCATGGTGGTGGTTGTTTCTGACGATTACCTGCAAAGTAAGGAATGTGACTTCCAGACCAAGTTTGCTCTCAGCCTGTCTCCAGGTGTCCAACAGAAGCGACTGATCCCTATCAAGTACAAAGCAATGAAGAAGGACTTTCCTAGTATCCTACGGTTCATCACTATCTGTGACTATACCAACCCTTGCACCAAGTCCTGGTTCTGGACCCGTCTTGCCAAGGCTCTGTCCCTGCCCTGA

>Apodemus sylvaticus

ATGTCTGCGGGAGGCCCCCGCGTGGGATCCGTGTCCGTGGACTCCTCCATGTTCTCCATACCCTTGGTCGCGCTCAACGTGGGAGTAAGGCGCCGCCTCTCACTGTTCTTGAACGTGAGGACAACCGTGGCAGCCGACTGGACTGTGCTGGCTGAGGAGATGGGCTTCGAGTACTTGGAGATCCGAGAGCTAGAGACGCGCCCCGACCCCACTCGCAGCTTGTTGGATGCCTGGCAGGGGCGCTCTGGCGCGTCGGTCGGCAGGCTGCTAGAGATGCTGGCCTTGTTAGACCGTGAGGATATACTGGAGGAGCTGAAGCATCGCATCGAGGAGGACTGCCGGAAGTACTTAGAGAAGCTGCGGAGCCAGGAGTCGGAGAAGCCCTTACAGGTGGCCAGAGTGGAAAGCAGTTTCCCGCAAACGGAGGAGCTGGGAGGCATCACCACCCTTGATGACCCTCTGGGACAAAGGCCGGAGCTTTTCGACGCCTTCATCTGCTACTGCCCCAACGATATTGAGTTTGTGAAGGAGATGATCCAGCAGCTAGAACAGACAGACTATCGGCTCAAGTTGTGTGTGTCCGACCGTGACGTCCTGCCGGGCACCTGTGTCTGGTCCATTGCCAGCGAGCTGATTGAGAAAAGGTGTCGCCGCATGGTGGTGGTTGTTTCTGATGATTATCTACAGAGCAAGGAATGTGACTTCCAGACCAAGTTTGCTCTCAGCCTGTCTCCAGGTGTCCAACAGAAGCGACTGATTCCTATCAAATACAAGTCGATGAAGAAGGACTTTCCCAGCATCCTGCGGTTCATCACTATATGCGACTATACCAACCCTTGCACCAAGTCCTGGTTCTGGACCCGCCTTGCCAAGGCTTTGTCCCTGCCCTGA

>Arvicanthis niloticus

ATGTCTGCGGGAAGCCCCGGCGTGGGATCCGTGTCCCTGGACTCCTCCATGTTCTCCATACCCTTGGTCGCACTCAACGTGGGAGTGAGACGCCGCCTCTCGCTATTCTTGAACGTTCGGACGCCCGTGGCAGCCGACTGGACCGTGCTGGCAGAGGAGATGGGCTTCGAGTACTTGGAGATCCGAGAGCTGGAGACACGCCCCGATCCCACTCGCAGTTTGTTGGATGCCTGGCAGGGACGCTCTGGCGCGTCGGTCGGCAGGCTGCTAGAGATGCTGGCCTTGTTAGACCGTGAGGATATACTGAAGGAGCTGAGGCCTCGCATCGAGGAGGACTGCCGGAAATACATAAAGAAGCTGCAGGACCAGGAGTCTGAGAAGCCCTTACAGGTGGCCAGAGTGGAAAGCAGTGTCCCACAAACAAAGGAGCTGGGAGGCATCACCACCCTTGATGACCCCCTGGGACAAATGCCAGAGCTTTTTGATGCCTTCATCTGCTACTGCCCCAGTGATATTGGGTTTGTGCAGGAAATGATCCGGCAACTAGAACAGACAGACTATCGGCTTAAGTTGTGCGTGTCCGATCGTGATGTCCTGCCCGGCACCTGTGTCTGGTCCATTGCCAGCGAGCTCATTGAGAAAAGGTGTCGCCGCATGGTGGTGGTTGTTTCTGACGATTATCTACAGAGCAAGGAATGTGACTTCCAGACCAAGTTTGCTCTCAGCCTGTCTCCAGGTGTCCAGCAGAAGCGACTGATCCCTATCAAATACAAAGCTATGAAGAAGGACTTTCCCAGTATCCTGCGGTTCATCACTATCTGCGACTATACCAACCCTTGCACCAAGTCTTGGTTCTGGACCCGCCTTGCCAAGGCTTTGTCCCTGCCCTGA

>Grammomys surdaster

ATGTCTGCGAGAAGTCCCCGCGTGGGATCCGTGTCCCTGGACTCTTCCATGTTCTCCATACCCTTGGTCGCACTCAATGTGGGAGTGAGACGCCGCCTCTCGCTGTTCTTGAACGTTCGGACGCCGGTGGCAGCCGACTGGACCTTGCTGGCAGAGGAGATGGGCTTCGAGTACTTGGAGATCCGAGACCTGGAGACACGCCCCGATCCCACTCGCAGTTTGTTGGATGCCTGGCAGGGACGCTCTGGCGCGTCGGTCGGCAGGCTACTAGAGATGCTGGCCTTGTTAGACCGTGAGGATGTACTGAAGGAGCTGATGCCCCGCATCGAGGAGGACTGCCGGAAATACATAAAGAAGCAGCAGGACCAGGAGTCCGAGAAGCCCTTACAGGTGGCTAGAGTGGAAAGCAGTGTGCCACAAACAAAGGAACTAGGAGGCATCACCACCCTTGACGACCCCCTGGGACAAACGCCGGAGCTTTTCGATGCCTTCATCTGCTACTGCCCCAGTGATATTGAGTTTGTGCAGGAGATGATCCGGCAACTAGAACAGACAGACTATCGGCTTAAGTTGTGCGTGTCTGACCGTGACGTCCTGCCAGGCACCTGTGTCTGGTCCATTGCCAGCGAGCTCATTGAGAAAAGGTGTCGCCGCATGGTGGTGGTGATTTCTGATGATTATCTACAGAGCAAGGAATGTGACTTCCAGACCAAGTTTGCTCTCAGCCTGTCTCCAGGTGTCCAACAGAAGCGACTGATCCCTATCAAATACAAGGCGATGAAGAAGGACTTTCCCAGTATTCTGCGGTTCATCACTATCTGCGACTATACCAACCCTTGCACCAAGTCCTGGTTCTGGACCCGCCTCGCCAAGGCTTTGTCCCTGCCCTGA

>Mastomys coucha

ATGTCTGCGGGAGGCCCCCGCGTGGAATCCGCGTCCCTGGACTCTTTCATGTACTCCATACCCTTGGTCGCGCTTAACGTGAGAGTGAGGCGCCGTCTCTCGCTGTTCTTGAACGCGCGGACGCCCGTGGCGGCCGACTGGACTTTGCTGGCGGAGGAGATGGGCTTCGAGTACTTGGAGATCCGAGAGCTGGAGACGCGCCCCGACCCCACTCGCAGTTTGTTGGATGCCTGGCAGGGGCGCTCTGGCGCGTCGGTGGGCAAGCTGCTAGAGATGCTGGCCTTGTTAGACCGTGAGGATGTACTGAAGGAACTGAAGCTCAGCATCGAGGAGGATTGCCAGAAATACATAGAGAAGCAGCAGAACCAGGAGTCCGAGAAGCCCTTACAGGTGGCCAGAGTGGAAAGTAGCATCCCCCAAACAAAGGAGCTAGGAGGCATCACCACCCTGGACGACCCCCTGGGACAAACGCCGGAGCTTTTCGATGCCTTCATCTGCTACTGCTCCAGTGATATCGAGTTTGTGCAGGAGATGATCCGGCAACTAGAACAGACAGACTATCGGCTTAAGTTGTGTGTGTCCGACCGCGATGTCTTGCCTGGCACCTGTGTCTGGTCCATCGCCAGTGAGCTGATTGAGAAAAGGTGTCGCCGCATGGTGGTAGTTGTTTCTGACGATTATCTACAGAGCAAGGAATGTGACTTCCAGACCAAGTTTGCTCTCAGCCTGTCTCCAGGCGTCCAGCAGAAGCGATTGATTCCTATCAAATACAAGGCGATGAAGAAAGACTTTCCCAGTATCCTGCGGTTCATCACTATATGCGACTATACAAACCCTTGCACCAAGTCCTGGTTCTGGACCCGCCTTGCCAAGGCTTTATCCTTGCCCTGA

>Mus caroli

ATGTCTGCGGGAGACCCCCGCGTGGGATCCGGTTCCCTGGACTCCTTCATGTTTTCCATACCCTTGGTCGCGCTTAACGTGGGAGTGAGGCGCCGCCTATCGCTGTTCTTGAACCCTCGGACGCCCGTGGCGGCCGACTGGACCTTGCTGGCGGAGGAGATGGGCTTCGAGTACTTGGAGATCCGAGAGCTGGAGACGCGCCCCGACCCCACTCGCAGTTTGTTGGATGCCTGGCAGGGGCGCTCTGGCGCGTCGGTCGGCAGGCTGCTAGAGCTGCTGGCCTTGTTAGACCGTGAGGATATACTGAAGGAGCTGAAGTCCCGCATCGAGGAGGACTGCCAGAAATACTTAGGTAAGCTGCAGAACCAGGAGTCTGAGAAGCCCTTACAGGTGGCCAGAGTGGAAAGCAGTGTCCCACAAACAAAGGAACTGGGAGGCATCACCACCCTTGATGACCCCCTAGGACAAACGCCGGAACTTTTCGATGCCTTTATCTGCTACTGCCCCAATGATATCGAGTTTGTGCAGGAGATGATCCGGCAACTGGAACAGACAGACTATCGGCTTAAGTTGTGTGTGTCCGACCGGGACGTCCTGCCGGGCACCTGTGTCTGGTCCATTGCCAGCGAGCTAATTGAGAAAAGGTGTCGCCGCATGGTGGTGGTTGTTTCTGACGATTATCTACAGAGCAAGGAATGTGACTTCCAGACCAAGTTTGCACTCAGCCTGTCTCCAGGTGTCCAACAGAAGCGACTGATTCCTATTAAATACAAGGCGATGAAGAAGGACTTTCCCAGTATCCTACGGTTCATCACTATATGCGACTATACCAACCCTTGCACCAAGTCCTGGTTCTGGACCCGCCTTGCCAAGGCTTTGTCCCTGCCCTGA

>Mus musculus

ATGTCTGCGGGAGACCCCCGCGTGGGATCCGGGTCCCTGGACTCCTTCATGTTCTCCATACCCTTGGTCGCGCTTAACGTGGGAGTGAGGCGCCGCCTATCGCTGTTCTTGAACCCTCGGACGCCCGTGGCGGCCGACTGGACCTTGCTGGCGGAGGAGATGGGCTTCGAGTACTTGGAGATCCGAGAGCTGGAAACGCGCCCTGACCCCACTCGCAGTTTGTTGGATGCCTGGCAGGGGCGCTCTGGCGCGTCTGTCGGCAGGCTGCTAGAGCTGCTGGCCTTGTTAGACCGTGAGGATATACTGAAGGAGCTGAAGTCGCGCATCGAGGAGGACTGCCAGAAATACTTAGGTAAGCAGCAGAACCAGGAGTCCGAGAAGCCTTTACAGGTGGCCAGAGTGGAAAGCAGTGTCCCACAAACAAAGGAACTGGGAGGCATCACCACCCTTGATGACCCCCTAGGACAAACGCCGGAACTTTTCGATGCCTTTATCTGCTACTGCCCCAACGATATCGAGTTTGTGCAGGAGATGATCCGGCAACTAGAACAGACAGACTATCGGCTTAAGTTGTGTGTGTCCGACCGTGACGTCCTGCCGGGCACCTGTGTCTGGTCCATTGCCAGCGAGCTAATTGAGAAAAGGTGTCGCCGCATGGTGGTGGTTGTTTCTGACGATTATCTACAGAGCAAGGAATGTGACTTCCAGACCAAGTTTGCACTCAGCCTGTCTCCAGGTGTCCAACAGAAGCGACTGATTCCTATTAAATACAAGGCGATGAAGAAGGACTTTCCCAGTATCCTGCGGTTCATCACTATATGCGACTATACCAACCCTTGCACCAAGTCCTGGTTCTGGACCCGCCTTGCCAAGGCTTTGTCCCTGCCCTGA

>Mus pahari

ATGTCTGCGGGAGGCCCCCGCGTGGGATCCGTGTCCCTGGACTCCCTCATGTTCTCCATACCCTTGGTCGCGCTTAACGTGGGAGTGAGGCGCCGCCTATCGCTGTTCTTGAACCCTCGGACTCCCGTGGCGGCCGACTGGACCTTGCTGGCGGAGGAGATGGGCTTCGAGTACTTGGAGATCCGAGAGCTGGAGACGCACCCCGACCCCACTCGCAGTTTGTTGGATGCCTGGCAGGGGCGCTCTAGCGCGTCGGTCGGCAGGCTGCTAGAGTTGCTGGCCTTGTTAGACCGTGAGGATATACTGAAGGAGCTGAAGTCCCGCATCGAGGAGGACTGCCAGAAATACTTAGAGAAGCAGCAGAGCCAGGAGTCCGAGAAGCCCTTACAGGTGGCCAGAGTGGAAAGCAGTATCCCACAAACAAAGGAACTGGGAGGCATCACCACCCTTGATGACCCCCTGGGACAAACACCAGAGCTTTTCGATGCCTTTATCTGCTACTGCCCCAATGATATCGAGTTTGTGCAGGAGATGATCCGGCAACTAGAACAGACAGACTATCGGCTTAAGTTGTGCGTGTCCGATCGTGACGTCCTGCCGGGCACCTGTGTCTGGTCCATTGCCAGTGAGCTAATTGAGAAAAGGTGTCGCCGCATGGTAGTGGTTGTTTCTGATGATTATCTACAAAGCAAGGAATGTGACTTCCAGACCAAGTTTGCACTCAGCCTGTCTCCAGGCGTCCAACAGAAGCGACTGATTCCTATTAAATACAAGGCGATGAAGAAGGACTTTCCCAGTATCCTGCGGTTCATCACTATATGTGACTATACCAACCCTTGCACCAAGTCCTGGTTCTGGACCCGCCTTGCCAAGGCTTTGTCCCTGCCCTGA

>Rattus rattus

ATGTCTGCGGGAGGCCCCCGCGTGGGATCCGTGTCCGTGGACTCCTACTTGTTCTCCCTACCGTTGGTCGCGCTCAACGTGGGAGTGAGGCGCCGCCTCTCGCTGTTCTTGAACCCGCGGACAACCGCGGCGGCCGACTGGACCTCGCTGGCGGAGGAGATGGGTTTCGAGTACTTGGAGATCCGCGAGTTTGAGTCGCGCTCCGATCCCACTCGCAGTTTGTTGGATGCCTGGCAGGGGCGCTCTGGCTCGTCGGTCGGCAGGCTGCTAGAGTTGCTAGCCTTGTTAGACCGTGAGGATATACTGAATGAACTGAAGGACCGCATCGAGGAGGACTGCCAGAAATACATACGCAACCAGCAGAAACAGGAGTCTGAGAAGCCCTTGCAGGTGGCCAGAGTGGAGAGCAGTGTCCCACAGACAAAGGAACTGGGAGGCATCACCACCCTCGATGACCCCCTGGGGCAAACGCCGGAGCTTTTCGACGCCTTCATCTGCTACTGCCCCAATGATATTGAGTTTGTGCAGGAGATGATCCGGCAACTAGAACAGACAGACTATCGGCTTAAATTGTGTGTGTCCGACCGCGATGTCCTGCCTGGCACCTGCGTGTGGTCCATTGCCAGCGAGCTCATTGAGAAAAGGTGTCGTCGCATGGTGGTGGTTGTTTCTGATGATTACCTGCAGAGTAAGGAATGTGACTTCCAGACCAAGTTTGCTCTCAGCCTGTCTCCAGGTGTCCAACAGAAGCGACTGATCCCTATCAAGTACAAAGCAATGAAGAAGGACTTTCCTAGTATCCTACGGTTCATCACTATCTGCGACTATACCAACCCTTGCACCAAGTCCTGGTTCTGGACCCGTCTTGCCAAGGCTCTGTCCCTGCCCTGA

>Acomys russatus

ATGTCTGCGGGAAGCCCTCGCGCGGGACCCGTGTCCCTGGACTCCTCCAAATCCTCCCTACCCTTGGCAGCGCTCAACGTGGGAGTGCGGCGCCGCCTCTCGCTGTTCTTAAACGTACGCACGCTGGTGGCGGCCGACTGGACCTCGCTGGCGGAGGAGATGGGCTTCGAATACTTGGAGATCCGAGAGCTGGAGACTCGCCCTGACCCCACCCGCAGTTTGTTGGATGCCTGGCAGGGGCGCCCTGGCGCGACGGTCGGCCGGCTGCTAGAGATGCTGGCCTTATTGGGACGAGAGGATATACTGAACGACCTGGGGCCCCTCATCGAGGAGAACTGCCAGAAATACATCCATAGTCAGCAGAAACAAGAGTCTGAGAAGCCCGTACAGGTGGCCAGAGTGGAAAGCAGTGTCCCACAAACAAAGGAGCTGGAAGGCATCACCACTCTTGATGACCCTTTGGGACAAACGCCAGAGCTTTTTGATGCTTTCATCTGCTACTGCCCCAGTGATATTGAGTTTGTGCAGGAGATCATCCGGCAACTAGAACAGACAGACTACCGGCTGAAGTTGTGCGTGTCTGACCGTGACGTCCTGCCAGGCACTTGTGTCTGGTCCATTGCCAGTGAGCTCATTGAGAAAAGGTGTCGCCGGATGGTGGTGGTTGTTTCTGATGATTACCTACAGAGCAAGGAATGTGACTTCCAGACCAAGTTTGCTCTCAGCCTCTCTCCAGGTGTCCAGCAGAAGCGACTGATTCCCATCAAATACAAGGCGATGAAGAAGGACTTCCCCAGTATCCTGCGGTTCATCACTATCTGTGACTATACCAACCCTTGCACCAAGTCCTGGTTCTGGACCCGCCTTGCCAAGGCTTTGTCCCTGCCCTGA

>Meriones unguiculatus

ATGTCAGAGGGAAGCCCTCCCGTGGGATCTGTGCGCCTGGACTCCTCCATGCACTCCCTCCCCTTGGCCGCGCTCAACGTGGGAGTGCGGCGCCGCCTCTCGCTGTTCTTGAACCTACGGACGCTGGTGGCGGCCGACTGGACCTCGCTGGCGGAGGAGATGGGCTTCGAGTACTTGGAGATCCGAGAACTGGAGAGTCGCCCCGACCCCACCCGCAGTCTGTTGGATGCCTGGCAGGGGCGCTCTGGCGCGTCGGTCGGCAGGCTGCTAGACATGCTGGCCCTGCTGGACCGGCAAGATATACTGCTGGAACTGAAGGACCTCATCGAGGATGACTGCAGGAAATACTTAGTAAATAAGCAGAAGCAGGAGTCTGAGAAGCCGTTGCAGGTGCCCAGAGTGGAAAGCAGTGTCCCACAAACAAAGGAGCTGGGCGGCATCACGACTCTTGATGACCCTCTGGGACAGACACCGGAGCTTTTCGACGCCTTCATCTGCTACTGCCCCAGTGATATTGAATTCGTGCAGGAGATGATCCGGCAGCTCGAACAGACAGACTATCGGCTGAAGCTGTGTGTGTCCGACCGTGATGTCCTGCCAGGCACCTGCGTCTGGTCTATTGCCAGTGAGCTCATTGAGAAAAGGTGTCGCCGCATGGTGGTGGTTGTTTCTGACGATTACCTACAGAGCAAGGAATGTGACTTCCAGACCAAGTTTGCCCTCAGCCTCTCCCCAGGTGTCCAACAGAAGAGGCTGATTCCCATCAAGTACAAGGTGATGAAGAGGGAGTTCCCCAGTATCCTGAGGTTCATCACCATCTGCGACTATACCAATCCTTGCACCAAGTCCTGGTTCTGGACCCGCCTTGCCAAGGCTTTGTCCCTGCCCTGA

>Psammomys obesus

ATGTCGGAAAGAAGCCCTCGCGTGGGATCCGTGCGCCTGGACTCCTCCATGTACTCCCTCCCCTTGGCCGCGCTCAACGTGGGAGTGCGGCGCCGCCTCTCGCTGTTCTTGAACCTACGGACGCTGGTGGCGGCCGACTGGACCTCGCTGGCGGAGGAGATGGGCTTCGAGTACTTGGAGATCCGAGAACTGGAGAGTCGCCCCGACCCCACCCGCAGTCTGTTGGATGCCTGGCAGGGGCGCTCTGGCGCGTCGGTCGGTAGGCTACTAGACATGCTGACGCTGCTGGACCGTCAAGATATACTGCTGGAACTGAAGGACCTCATCGAGGATGACTGCAGGAAATACTTAGTAAATAAGCAGAAGCAGGAGTCTGAGAAGCCTTTGCAGGTGCCCAGAGTGGAAAGCAGTGTCCCACAAACAAAGGAGCTGGGCGGCATCACGACTCTTGATGACCCCCTGGGACAGACACCGGAGCTTTTCGACGCCTTCATCTGCTACTGCCCCAGTGATATTGAATTTGTGCAGGAGATGATCCGGCAGCTCGAACAGACAGACTATCGGCTGAAGCTGTGTGTGTCCGACCGTGATGTCCTGCCAGGCACCTGCGTCTGGTCCATTGCCAGTGAGCTCATTGAGAAAAGGTGTCGTCGCATGGTGGTGGTTGTTTCTGACGATTACCTACAGAGCAAGGAATGTGACTTCCAGACCAAGTTTGCCCTCAGCCTCTCTCCAGGTGTCCAACAGAAGAGGCTGATTCCCATCAAGTACAAGGTGATGAAGAAGGAGTTCCCCAGTATCCTGAGGTTCATCACCACCTGCGACTATACCAATCCTTGCACCAAGTCCTGGTTCTGGACCCGCCTTGCCAAGGCTTTGTCCCTGCCCTGA

Supplementary Data 3. Aligned sequences of SARM1

>Rattus norvegicus

ATGGTCCTGACGCTGCTCTTCTCCGCCTACAAACTGTGCCGCTTCTTCATCATGTCAGGCCCACGGCCGGGCGCCGATCGGCTGACAGTACCGGGACCGGATCGGAGTGGCGGCACCAGCCCATGGTGGGCTGCGGGCGGTCGCGGGTCTCGCGAAGTGTCGCCCGGGGTGGGCACCGAGGTGCAAGGCGCGCTGGAGCGTTCGCTGCCGGAGCTGCAGCAGGCGCTGTCCGAGCTGAAACAGGCAAGCGCGGCGCAAGCGGTGGGCGCGGGCCTCGCCGAGGTCTTCCAGCTGGTAGAGGAAGCCTGGCTGCTGCCGGCCGTGGGCCGCGAGGTGGCCCAAGGTTTGTGCGATGCTATACGTCTGGACGGTGGCCTCGACTTGCTGTTGCGGCTGCTTCAGGCACCGGAGCTAGAGACCCGCGTGCAGGCCGCGCGCTTGCTGGAGCAGATCCTGGTGGCTGAGAACCGGGACCGCGTAGCGCGCATCGGCCTAGGCGTGATCTTGAACCTGTCGAAGGAACGCGAGCCTGTGGAGCTGGCACGGAGCGTGGCGGGCATCTTGGAGCACATGTTCAAGCACTCGGAGGAGACCTGCCAGCGGCTGGTGGCGGCCGGAGGCCTCGACGCGGTGCTGTATTGGTGCCGCCGCACAGACCCAGCGCTACTGCGCCACTGCGCGCTGGCGCTGGCAAACTGCGCGCTGCACGGGGGCCAGACTGTGCAGCGGTGCATGGTGGAGAAGCGCGCCGCCGAGTGGCTCTTCCCGCTCGCTTTCTCCAAAGAGGACGAGTTGCTGCGGCTGCATGCTTGCCTGGCGGTGGCCGTGTTGGCTACCAACAAGGAGGTAGAACGCGAGGTCGAGCACTCAGGCACGTTGGCGCTCGTCGAGCCGCTGGTGGCATCGCTGGATCCTGGCCGCTTCGCTCGTTGCCTGGTGGATGCCAGTGACACAAGCCAGGGTCGTGGACCAGACGACCTGCAGAGCCTGGTGCTGTTGCTCGATTCGTCGCGTTTGGAGGCTCAGTGCATAGGGGCTTTCTACCTGTGCGCCGAGGCTGCCATCAAAAGCCTACAGGGAAAGACCAAGGTATTCAGCGACATCGGCGCCATCCAGAGCCTGAAACGCCTGGTTTCTTACTCCACAAATGGCACCACGTCGACGCTGGCCAAGCGCGCGCTGCGCCTATTGGGCGAGGAGGTGCCAAGGCGCATCCTACCCTGCGTGGCCAGCTGGAAGGAGGCTGAGGTCCAGACCTGGCTGCAGCAGATCGGCTTCTCCCAGTACTGCGAGAACTTTCGGGATCAGCAGGTAGATGGTGATTTGCTTCTACGACTCACAGATGAAGAACTCCAGACAGACCTAGGCATGAAATCAAGCATCACCCGCAAGAGGTTCTTTAGGGAGCTCACGGAGCTCAAGACCTTCGCCAGCTATGCTACTTGCGACCGCAGCAACCTAGCAGACTGGCTGGGCAGCCTGGATCCTCGCTTCCGCCAGTACACCTATGGCCTGGTCAGCTGCGGCCTGGACCGCTCCCTGCTGCACCGCGTGTCAGAGCAGCAGCTCCTGGAGGACTGTGGCATCCGCCTGGGGGTGCATCGCACGCGCATCCTCTCTGCAGCCAGAGAAATGCTACATTCCCCGCTGCCCTGTACCGGAGGCAAGCCCAGTGGAGACACCCCAGATGTCTTTATCAGCTACCGCAGGAACTCGGGTTCCCAGCTGGCCAGCCTCCTGAAGGTGCACCTGCAGCTGCATGGCTTCAGTGTCTTCATCGATGTGGAGAAGCTGGAGGCCGGCAAATTCGAGGACAAGCTCATCCAGAGTGTCATGGCGGCTCGAAATTTTGTGCTGGTGCTGTCTGCGGGAGCGCTGGATAAGTGCATGCAGGACCATGAATGCAAGGACTGGGTGCACAAGGAGATCGTGACTGCCTTAAGCTGTAGCAAGAACATTGTGCCCATCATTGATGGCTTTGAGTGGCCTGAGCCTCAGGCACTGCCTGAGGATATGCAGGCTGTACTCACCTTCAACGGCATCAAATGGTCTCATGAGTACCAGGAGGCCACCATCGAGAAAATCATCCGCTTCCTACAGGGTCGCCCCTCTCAGGACTCCTCTGCCGGCTCTGACACCAGTTTGGAGGGAGCTACATCAATGGGTCTGCCTTAA

>Rattus tanezumi

ATGGTCCTGACGCTGCTCTTCTCCGCCTACAAACTGTGCCGCTTCTTCATCATGTCAGGCCCACGGCCGGGCGCCGATCGGCTGACAGTACCGGGACCGGATCGGAGTGGCGGCACCAGCCCATGGTGGGCTGCGGGCGGTCGCGGGTCTCGCGAAGTGTCGCCCGGGGTGGGCACCGAGGTGCAAGGCGCGCTGGAGCGTTCGCTGCCGGAGCTGCAGCAGGCGCTGTCCGAGCTGAAACAGGCAAGCGCGGCGCAAGCGGTGGGCGCGGGCCTCGCCGAGGTCTTCCAGCTGGTAGAGGAAGCCTGGCTGCTGCCGGCCGTGGGCCGCGAGGTGGCCCAAGGTTTGTGCGATGCTATACGTCTGGACGGTGGCCTCGACTTGCTGTTGCGGCTGCTTCAGGCACCGGAGCTAGAGACCCGCGTGCAGGCCGCGCGCTTGCTGGAGCAGATCCTGGTGGCTGAGAACCGGGACCGCGTAGCGCGCATCGGCCTAGGCGTGATCTTGAACCTGTCGAAGGAACGCGAGCCTGTGGAGCTGGCACGGAGCGTGGCGGGCATCTTGGAGCACATGTTCAAGCACTCGGAGGAGACCTGCCAGCGGCTGGTGGCGGCCGGAGGCCTCGACGCGGTGCTGTATTGGTGCCGCCGCACAGACCCAGCGCTACTGCGCCACTGCGCGCTGGCGCTGGCAAACTGCGCGCTGCACGGGGGCCAGACTGTGCAGCGGTGCATGGTGGAGAAGCGCGCCGCCGAGTGGCTCTTCCCGCTCGCTTTCTCCAAAGAGGACGAGTTGCTGCGGCTGCATGCTTGCCTGGCGGTGGCCGTGTTGGCTACCAACAAGGAGGTAGAACGCGAGGTCGAGCACTCAGGCACGTTGGCGCTCGTCGAGCCGCTGGTGGCATCGCTGGATCCTGGCCGCTTCGCTCGTTGCCTGGTGGATGCCAGTGACACAAGCCAGGGTCGTGGACCAGACGACCTGCAGAGCCTGGTGCTGTTGCTCGATTCGTCGCGTTTGGAGGCTCAGTGCATAGGGGCTTTCTACCTGTGCGCCGAGGCTGCCATCAAAAGCCTACAGGGAAAGACCAAGGTATTCAGCGACATCGGCGCCATCCAGAGCCTGAAACGCCTGGTTTCTTACTCCACAAATGGCACCACGTCGACGCTGGCCAAGCGCGCGCTGCGCCTATTGGGCGAGGAGGTGCCACGGCGCATCCTACCCTGCGTGGCCAGCTGGAAGGAGGCTGAGGTCCAGACCTGGCTGCAGCAGATCGGCTTCTCCCAGTACTGCGAGAACTTTCGGGATCAGCAGGTAGATGGTGATTTGCTTCTACGACTCACAGATGAAGAACTCCAGACAGACCTAGGCATGAAATCAAGCATCACCCGCAAGAGGTTCTTTAGGGAGCTCACTGAGCTCAAGACCTTCGCCAGCTATGCTACTTGCGACCGCAGCAACCTAGCAGACTGGCTGGGCAGCCTGGATCCTCGCTTCCGCCAGTACACCTATGGCCTGGTCAGCTGCGGCCTGGACCGCTCCCTGCTGCACCGCGTGTCAGAGCAGCAGCTCCTGGAGGACTGTGGCATCCGCCTGGGGGTGCATCGCACGCGCATCCTCTCTGCAGCCAGAGAAATGTTACATTCCCCGCTGCCCTGTACCGGAGGCAAGCCCAGTGGAGACACCCCAGATGTCTTTATCAGCTATCGCAGGAACTCGGGTTCCCAGCTGGCCAGCCTCCTGAAGGTGCACCTGCAGCTGCATGGCTTCAGTGTCTTCATCGATGTGGAGAAGCTGGAGGCCGGCAAATTCGAGGACAAGCTCATCCAGAGTGTCATGGCGGCTCGAAATTTTGTGCTGGTGCTGTCTGCGGGAGCGCTGGATAAGTGCATGCAGGACCATGAATGCAAGGACTGGGTGCACAAGGAGATCGTGACTGCCTTAAGCTGTAGCAAGAACATTGTGCCCATCATTGATGGCTTTGAGTGGCCTGAGCCTCAGGCACTGCCTGAGGATATGCAGGCTGTACTCACCTTCAACGGCATCAAATGGTCTCATGAGTACCAGGAGGCCACCATCGAGAAAATCATCCGCTTCCTACAGGGTCGCCCCTCTCAGGACTCCTCTGCCGGCTCTGACACCAGTTTGGAGGGAGCTACATCAATGGGTCTGCCTTAA

>Apodemus-sylvaticus

ATGGTCCTGACGCTGCTCTTCTCCGCCTACAAACTGTGCCGCTTCTTCACCATGTCAGGCCCACGGCCAGGCGCCGATCGGCTGACAGTGCCGGGACCGGATCGGAGTGGCGGCGCCAGTCCATGGTGGGCTGCGGGCGGTCGCGGGTCTCGCGAAGTGTCGCCCGGGGTGGGCACCGAGGTTCAAGGCGCCCTGGAGCGTTCGCTGCCGGAGCTGCAGCAGGCGCTGTCCGAACTGAAACAGGCAAGCGCGGCGCGGGCTGTTGGCGCGGGCCTCGCTGAGGTCTTCCAGCTGGTAGAGGAAGCCTGGCTGCTGCCGGCCGTGGGCCGCGAGGTGGCCCAGGGTCTATGCGATGCTATACGTCTGGACGGTGGCCTCGACTTGCTGCTGCGGCTGCTTCAGGCACCGGAGCTAGAGACCCGTGTGCAGGCCGCGCGCTTGCTGGAGCAGATCCTGGTGGCTGAGAACCGGGACCGGGTAGCGCGCATCGGCTTAGGTGTGATCTTGAACCTGGCGAAGGAGCGCGAGCCTGTGGAGCTGGCACGGAGCGTGGCGGGCATCTTGGAGCATATGTTCAAGCACTCGGAGGAGACATGCCAGCGGCTGGTGGCGGCGGGAGGCCTGGACGCGGTGCTGTACTGGTGCCGCCGCACAGACCCGGCGCTACTGCGCCACTGCGCGCTGGCGCTGGCGAACTGCGCGCTGCACGGGGGCCAGACCGTGCAGCGGTGCATGGTGGAGAAGCGCGCCGCCGAGTGGCTCTTCCCGCTCGCTTTCTCCAAGGAGGATGAGCTGCTGCGGCTGCACGCCTGCCTGGCGGTGGCGGTGTTGGCTACCAACAAGGAGGTGGAACGCGAGGTCGAGCACTCCGGCACGTTGGCACTCGTCGAGCCACTCGTGGCGTCGCTGGATCCCGGCCGCTTCGCCCGCTGCCTGGTGGATGCCAGTGACACAAGCCAGGGTCGTGGACCAGACGACCTGCAGAGCCTGGTGCTGTTGCTCGATTCGTCGCGTTTGGAGGCTCAGTGCATAGGGGCATTCTATCTGTGCACGGAGGCTGCCATCAAGAGCCTACAGGGAAAGACCAAGGTGTTCAGTGACATCGGCGCCATTCAGAGCCTCAAACGCCTGGTTTCTTACTCTACAAATGGCACTACGTCGGCGCTGGCCAAGCGCGCGCTGCGCCTATTGGGCGAGGAGGTGCCAAGGCGCATCCTGCCCTGCGTGGCCAGCTGGAAGGAGGCTGAGGTCCAGACCTGGCTGCAGCAGATCGGCTTCTCCCAGTACTGCGAGAACTTTCGGGATCAGCAGGTAGATGGTGACCTGCTTCTGCGACTCACAGATGAAGAACTCCAGACAGACCTAGGCATGAAATCAAGCATCACCCGCAAGAGGTTCTTCAGGGAGCTCACGGAGCTCAAGACCTTCGCCAGCTATGCTACCTGCGACCGCAGCAATCTAGCGGACTGGCTGGGCAGCCTGGATCCTCGCTTCCGTCAGTACACCTATGGCCTGGTCAGCTGCGGCCTGGACCGCTCCCTGCTGCACCGAGTGTCAGAGCAGCAGCTCCTGGAGGACTGTGGCATCCGCCTGGGGGTGCATCGCACACGCATCCTCTCTGCAGCCAGAGAAATGCTCCATTCTCCGCTGCCCTGTACTGGAGGCAAACCCAGTGGAGACACCCCAGATGTCTTTATCAGCTACCGGAGGAACTCGGGTTCCCAGCTAGCCAGCCTCCTGAAGGTGCACCTGCAGCTGCATGGCTTCAGCGTCTTCATCGACGTGGAGAAGCTGGAAGCCGGCAAATTCGAGGACAAACTCATCCAGAGCGTCATGGCGGCTCGAAATTTTGTGCTGGTGCTGTCTGCCGGGGCGCTGGATAAGTGCATGCAGGACCATGACTGTAAGGACTGGGTGCACAAGGAGATTGTGACTGCTTTAAGCTGTGGCAAGAACATTGTGCCCATCATCGATGGCTTTGAGTGGCCTGAGCCTCAGGCGCTGCCTGAAGATATGCAGGCCGTGCTCACCTTCAACGGCATCCAATGGTCCCATGAGTACCAGGAGGCCACCATTGAGAAGATCATCCGCTTCCTACAGGGTCGCTCCTCTCAGGACTCCTCTGTCGGCTCTGATACCAGTTTGGAGGGAGCTACGCCAATGGGTCTGCCTTAA

>Arvicanthis-niloticus

ATGGTCCTGACGCTGCTCTTCTCCGCCTACAAACTGTGCCGCTTCTTCACCATGTCAGGCCCACGGCCGAGTGCCGATCGGCTGACAGTGCCGGGACCGGATCGGAGTGGCGGCGCCAGCCCATGGTGGGCTGCGGGCGGTCGCGGTTCTCGCGAAGTGTCGCCCGGGGTGGGCACCGAGGTGCAAGGCGCCCTGGAGCGTTCGCTACCAGAGCTGCAGCAGGCGCTGTCCGAGCTGAAACAGGCAAGCGCGGCACGCGCTGTGGGCGCGGGCCTCGCCGAGGTTTTCCAGCTGGTAGAGGAGGCCTGGCTGCTGCCTGCCGTGGGCCGCGAGGTGGCCCAGGGTCTATGCGATGCTATACGTCTGGATGGTGGCCTCGACTTGCTGTTGCGGCTGCTTCAGGCACCGGAGCTAGAGACCCGAGTACAGGCCGCACGCTTGCTGGAGCAGATCCTGGTGGCTGAGAACCGGGACCGCGTGGCACGCATCGGCCTAGGCGTGATCTTGAACCTGGCGAAGGAGCGCGAGCCTGTGGAGCTGGCACGGAGTGTGGCAGGCATCTTGGAGCACATGTTCAAGCACTCGGAGGAGACGTGCCAGAGGCTGGTGGCGGCCGGAGGCCTCGACGCAGTGCTGTACTGGTGCCGCCGCACAGACCCGGCGCTGCTGCGCCATTGCGCGCTGGCACTGGCGAACTGCGCGCTGCACGGGGGCCAGACGGTGCAGCGATGCATGGTGGAGAAGCGCGCCGCCGAGTGGCTCTTCCCGCTCGCTTTCTCCAAGGAGGACGAGCTGCTGAGGCTGCACGCCTGCCTGGCGGTGGCGGTGTTGGCTACCAACAAGGAGGTGGAACGCGAGGTCGAGCACTCCGGCACGTTGGCGCTTGTCGAGCCCCTCGTGGCATCGCTGGATCCCGGCCGCTTCGCCCGCTGCCTGGTGGATGCCAGTGACACAAGCCAAGGTCGTGGACCAGACGACCTGCAGAGCCTGGTGCTGTTGCTCGATTCGTCTCGTTTGGAGGCTCAGTGCATAGGGGCATTCTACCTGTGCGCCGAAGCTGCCATCAAGAGCCTACAGGGAAAGACCAAGGTGTTCAGCGACATCGGCGCCATCCAGAGCCTGAAACGCTTGGTTTCTTACTCAACAAATGGCACCACGTCGGCGCTGGCCAAGCGGGCACTGCGCCTACTGGGCGAGGAGGTGCCAAGGCGCATCCTGCCCTGCGTGGCCAGCTGGAAGGAGGCTGAGGTCCAGACCTGGCTGCAGCAGATCGGCTTCTCCCAGTACTGCGAGAACTTTCGGGATCAGCAGGTAGATGGTGACTTGCTTCTACGACTCACAGATGAAGAACTCCAGACAGACCTAGGCATGAAATCAAGCATCACCCGCAAGAGGTTCTTTAGGGAGCTCACGGAGCTCAAGACCTTCGCCAGCTATGCTACTTGCGACCGCAGCAACCTAGCGGACTGGCTGGGCAGCCTGGATCCTCGCTTCCGCCAGTACACCTATGGCCTGGTCAGCTGCGGCCTGGACCGCTCCCTGCTGCACCGCGTGTCAGAGCAGCAGCTCCTGGAGGACTGTGGCATCCGCCTGGGGGTGCACCGCACGCGCATCCTCTCTGCAGCCAGAGAAATGCTGCATTCGCCGCTGCCCTGTACTGGGGGCAAGCCCAGTGGAGACACCCCAGATGTCTTTATCAGCTACCGGCGGAACTCAGGTTCCCAGCTGGCCAGCCTCCTGAAGGTGCACCTGCAGCTGCATGGCTTCAGTGTCTTCATCGATGTGGAGAAGCTGGAAGCTGGGAAATTCGAGGACAAACTCATCCAGAGTGTCATGGCAGCTCGAAATTTTGTACTGGTGCTGTCTGCTGGAGCGCTGGATAAGTGCATGCAGGACCATGACTGTAAAGACTGGGTGCACAAGGAGATTGTGACTGCTCTAAGCTGTGGCAAGAACATTGTACCCATCATTGATGGCTTTGAGTGGCCTGAGCCTCAGGCACTGCCTGAGGATATGCAAGCTGTACTCACCTTTAATGGCATCCAATGGTCCCATGAGTACCAGGAGGCCACCATTGAGAAGATCATCCGCTTCCTCCAGGGCCGCCCCTCTCAGGACTCCTCTGCTGGCTCCGATGCCAGTTTGGAGGGAGCTACACCAATGGGTCTGCCTTAA

>Grammomys-surdaster

ATGGTCCTGACGCTGCTCTTCTCCGCCTACAAACTGTGCCGCTTCTTCACCATGTCAGGCCCACGGCCGAGCGCCGATCGGCTGACAGTGCCGGGACCGGATCGGAGTGGCGGCGCCAGCCCATGGTGGGCTGCGGGCGGTCGCGGGTCTCGCGAAGTGTCTCCTGGGGTGGGCACTGAGGTGCAGGGCGCCCTGGAGCGTTCGCTGCCGGAGCTGCAGCAGGCGCTGTCCGAGCTGAAACAGGCAAGCGCGGCACGGGCCGTGGGCGCGGGCCTCGCCGAGGTCTTCCAGCTGGTAGAGGAAGCCTGGCTGCTGCCGGCCGTGGGCCGCGAGGTGGCCCAGGGTCTATGCGATGCTATACGTCTGGACGGTGGCCTCGACTTGCTGCTGCGGCTGCTTCAGGCACCGGAACTAGAGACCCGAGTGCAGGCCGCACGCTTGCTGGAGCAGATCCTGGTGGCTGAGAACCGGGACCGCGTGGCGCGCATCGGCCTAGGCGTGATCTTGAACCTGGCGAAGGAACGCGAGCCTGTGGAGCTGGCACGGAGCGTGGCAGGCATCTTGGAGCACATGTTCAAGCACTCGGAGGAGACGTGCCAGAGGCTGGTGGCGGCCGGAGGTCTCGACGCAGTGCTGTACTGGTGCCGCCGCACAGACCCGGCGCTGCTGCGCCACTGCGCGCTGGCGCTGGCGAACTGCGCGCTGCACGGGGGCCAGACGGTGCAGCGGTGCATGGTGGAGAAGCGCGCTGCGGAGTGGCTCTTCCCGCTCGCTTTCTCCAAGGAGGATGAGCTACTGCGGCTGCACGCTTGCCTGGCGGTGGCGGTGTTGGCTACCAACAAGGAGGTGGAACGCGAGGTCGAGCACTCCGGCACGTTGGCGCTCGTCGAGCCCCTCGTGGCATCACTGGATCCCGGCCGCTTCGCCCGCTGCCTGGTGGATGCCAGTGACACAAGCCAGGGTCGGGGACCAGACGACCTGCAGAGCCTGGTGCTGTTGCTCGATTCGTCGCGTTTGGAGGCTCAGTGCATAGGAGCATTCTACCTGTGCGCCGAGGCTGCCATCAAGAGCCTACAGGGAAAGACCAAGGTGTTCAGCGACATCGGCGCCATCCAGAGCCTGAAACGCCTGGTTTCTTACTCAACAAATGGCACCACATCAATGCTGGCCAAGCGCGCACTTCGCCTACTGGGCGAGGAGGTGCCAAGGCGCATCCTGCCCTGCGTGGCCAGCTGGAAGGAGGCTGAGGTCCAGACCTGGCTGCAGCAGATTGGCTTCTCCCAGTACTGCGAGAACTTTCGGGATCAGCAGGTAGATGGTGACTTGCTTCTACGACTCACAGATGAAGAACTCCAGACAGACCTAGCCATGAAATCAAGCATCACCCGCAAGAGGTTCTTTAGGGAGCTCACAGAGCTCAAGACCTTCGCCAGCTATGCTACTTGCGACCGCAGCAACCTAGCAGACTGGCTGGGCAGCCTGGATCCTCGCTTCCGCCAGTACACCTATGGCCTGGTCAGCTGTGGCCTGGACCGCTCCCTGCTGCACCGAGTGTCAGAACAGCAGCTCCTGGAGGACTGTGGCATCCGCCTGGGGGTGCATCGCACGCGCATCCTCTCTGCAGCCAGAGAAATGCTACATTCCCCACTGCCCTGTACTGGAGGCAAGCCCAGTGGAGACACCCCAGATGTCTTTATCAGCTACCGGAGGAACTCAGGTTCCCAGCTAGCCAGCCTCCTGAAGGTGCACCTGCAGCTGCATGGCTTCAGCGTCTTCATCGATGTGGAGAAGCTGGAAGCGGGCAAATTCGAAGACAAGCTCATCCAGAGTGTCATGGCGGCTCGAAATTTTGTACTGGTGCTGTCTGCTGGTGCGCTGGATAAGTGCATGCAGGACCATGACTGTAAAGACTGGGTACACAAGGAAATTGTGACTGCTTTAAGCTGTGGCAAGAACATCGTGCCCATCATTGATGGCTTTGAGTGGCCTGAGCCTCAGGCACTGCCTGAGGATATGCAGGCTGTACTCACCTTCAATGGCATCAAATGGTCCCATGAGTACCAGGAGGCCACCATTGAGAAGATCATCCGCTTCCTCCAGGGCCGCCCCTCTCAGGACTCCTCTGCTGGCTCCGATACCAGTTTGGAAGGAGCTACGCCAATGGGTCTGCCTTAA

>Mastomys-coucha

ATGGTCCTGACGCTGCTCTTCTCTGCCTACAAACTGTGCCGCTTCTTCACCATGTCAGGCCCACGGCCCGGCGCCGATCGGCTGACAGTGCCGGGACCGGATCGGAGTGGCGGTACCAGCCCATGGTGGGCTGCGGGCGGTCGCGGGTCTCGTGAAGTGTCGCCCGGGGTGGGCACCGAGGTGCAAGGCGCCCTGGAGCGTTCGCTGCCGGAGCTGCAGCAGGCGCTCTCCGAGCTGAAACAGGCAAGCGCGGCTCGGGCTGTGGGCGCGGGCCTCGCTGAGGTCTTCCAGCTGGTAGAGGAAGCCTGGCTGCTGCCGGCCGTGGGCCGCGAGGTAGCCCAGGGTCTATGCGATGCTATACGTCTGGATGGTGGCCTCGACTTGCTATTGCGGCTACTTCAGGCACCGGAGCTAGAGACCCGCGTGCAGGCCGCGCGCTTGCTGGAACAGATCCTGGTGGCTGAAAACCGGGACCGTGTGGCCCGCATCGGCCTAGGAGTGATCTTGAATCTGGCGAAGGAGCGCGAGCCTGTGGAGTTGGCACGGAGCGTGGCGGGCATCTTGGAGCACATGTTCAAGCACTCGGAGGAGACATGCCAGCGGTTGGTGGCGGCCGGAGGTCTCGACGCGGTGCTGTACTGGTGCCGCCGCACAGACCCGGCACTACTGCGCCACTGCGCGCTGGCGCTGGCGAACTGCGCGCTGCACGGGGGCCAGACGGTGCAGCGATGCATGGTGGAGAAGCGCGCCGCCGAGTGGCTCTTCCCGCTCGCCTTCTCCAAGGAGGACGAGCTGCTGCGGCTGCACGCCTGCCTGGCGGTGGCGGTGTTGGCTACCAACAAAGAGGTGGAACGCGAGGTCGAGCACTCCGGCACGTTGGCGCTCGTCGAGCCGCTCGTAGCATCGCTGGATCCCGGTCGCTTCGCCCGCTGCCTGGTGGATGCCAGTGACACGAGCCAGGGTCGTGGACCAGACGACCTGCAGAGCCTGGTGCTGTTGCTCGATTCGTCGCGTTTGGAGGCTCAGTGCATAGGGGCATTCTACCTGTGCGCAGAGGCTGCCATCAAGAGCCTACAGGGAAAGACCAAGGTGTTCAGCGACATCGGCGCCATCCAGAGCCTGAAACGCCTTGTTTCTTACTCTACAAATGGCACCACATCGGCGCTGGCCAAGCGCGCGCTGCGCCTATTGGGCGAGGAGGTGCCAAGGCGCATCCTGCCCTGCGTGGCCAGCTGGAAGGAGGCTGAGGTCCAGACCTGGCTGCAGCAGATCGGCTTCTCCCAGTACTGCGAGAACTTTCGGGATCAGCAGGTAGATGGCGACCTGCTTCTACGACTCACAGATGAAGAACTCCAGACAGACCTAGGCATGAAATCAAGCATCACACGGAAGAGGTTCTTTAGAGAGCTCACGGAGCTCAAGACCTTTGCCAGCTATGCTACTTGCGACCGCAGCAACCTAGCGGACTGGCTGGGCAGCCTGGATCCTCGCTTCCGCCAGTACACCTATGGCCTGGTCAGCTGCGGCCTGGACCGCTCCCTGCTGCACCGCGTGTCAGAGCAGCAGCTCCTGGAGGACTGTGGCATCCGCCTGGGGGTGCATCGCACGCGCATCCTCTCTGCAGCCAGAGAAATGCTACATTCCCCGCTGCCCTGTACTGGAGGCAAGCCCAGTGGAGACACCCCAGACGTCTTTATCAGCTATCGGAGGAACTCGGGTTCCCAGCTGGCCAGCCTCCTGAAGGTGCACCTGCAGCTTCACGGCTTCAGCGTCTTCATCGATGTGGAGAAGCTGGAGGCGGGCAAATTCGAGGACAAGCTCATCCAGAGTGTCATGGCGGCTCGAAACTTTGTACTGGTGCTGTCTGCTGGGGCGCTGGATAAGTGCATGCAGGACCATGACTGCAAGGACTGGGTGCACAAGGAGATTGTGACTGCTTTAAGCTGTGGCAAGAACATTGTGCCCATCATTGATGGCTTCGAGTGGCCTGAGCCTCAGGCACTGCCTGAGGACATGCAGGCAGTACTTACCTTCAACGGCATCAAATGGTCCCATGAGTACCAGGAGGCCACCATCGAGAAGATCATCCGCTTCCTACAGGGCCGCCCCTCTCAGGACTCCTCTGCCGGCTCTGATACCAGTTTGGAGGGAGCCACGCCAATGGGTCTGCCTTAA

>Mus-caroli

ATGGTCCTGACGCTGCTCTTCTCCGCCTACAAACTGTGTCGCTTCTTCACCATGTCAGGCCCACGGCCGGGCGCCGATCGGCTGACAGTGCCCGGACCGGATCGGAGTGGTGGCGCCAGCCCATGGTGGGCTGCGGGCGGTCGTGGGTCTCGCGAAGTGTCGCCCGGGGTGGGCACTGAGGTGCAAGGCGCCCTGGAGCGTTCGCTGCCGGAGCTGCAGCAGGCGCTGTCCGAGCTGAAACAGGCAAGCGCGGCGCGGGCTGTGGGCGCGGGCCTCGCCGAGGTCTTCCAGCTGGTAGAGGAAGCCTGGCTGCTGCCGGCCGTGGGCCGCGAGGTGGCCCAGGGTCTATGCGATGCTATACGTCTGGATGGTGGCCTCGACTTGCTGTTGCGGCTGCTTCAGGCACCGGAGCTAGAGACCCGTGTGCAGGCCGCGCGCTTGCTGGAGCAGATCCTGGTGGCTGAGAACCGGGACCGCGTGGCGCGCATTGGCCTAGGCGTGATCTTGAACTTGGCGAAGGAGCGCGAGCCTGTGGAACTGGCACGAAGCGTGGCGGGCATCTTGGAGCACATGTTCAAGCACTCGGAGGAGACGTGCCAGCGGCTGGTGGCGGCCGGAGGCCTCGACGCGGTGCTGTACTGGTGCCGTCGCACAGACCCGGCGCTACTGCGCCACTGCGCTCTTGCGCTGGCGAACTGCGCGCTGCACGGGGGCCAGACGGTGCAGCGGTGCATGGTGGAGAAGCGCGCCGCCGAGTGGCTCTTCCCGCTCGCTTTCTCCAAGGAGGACGAGCTGCTGCGGCTGCACGCCTGCCTGGCGGTGGCGGTGTTGGCTACCAACAAGGAGGTGGAACGCGAGGTCGAGCACTCCGGCACATTGGCGCTTGTCGAGCCGCTCGTGGCATCGCTGGACCCCGGCCGCTTCGCCCGCTGTCTGGTGGATGCCAGTGACACAAGCCAGGGTCGTGGACCAGACGACCTACAGAGCCTGGTGCTGTTGCTCGATTCGTCGCGTTTGGAGGCTCAGTGCATAGGAGCATTCTACCTGTGCGCAGAGGCTGCCATCAAGAGCCTACAGGGAAAGACCAAGGTGTTCAGCGACATCGGCGCTATCCAGAGCCTGAAACGCCTGGTTTCTTACTCTACGAATGGCACCACGTCGGCGCTGGCCAAGCGCGCGCTGCGCCTATTGGGCGAGGAGGTGCCAAGGCGCATCCTGCCCTGCGTGGCCAGCTGGAAGGAAGCTGAGGTCCAGACCTGGCTACAGCAGATCGGCTTCTCCCAGTACTGCGAGAAATTTCGGGAGCAGCAGGTAGATGGTGACCTGCTTCTAAGACTCACAGATGAAGAACTCCAGGCAGACCTAGGCATGAAGTCAAGCATCACTCGCAAGAGGTTCTTTAGGGAGCTCACGGAGCTCAAGACCTTCGCCAGCTACGCTACTTGCGACCGCAGCAACCTAGCGGACTGGCTGGGCAGCCTGGATCCTCGCTTCCGCCAGTACACCTATGGCCTGGTCAGCTGCGGTCTAGACCGCTCCCTGCTGCACCGCGTGTCAGAGCAGCAGCTCCTGGAGGACTGTGGCATCCGCCTGGGGGTGCACCGCACGCGCATCCTCTCTGCAGCCAGAGAAATGCTACATTCCCCGCTGCCCTGTACTGGAGGCAAGCTCAGTGGAGACACCCCAGATGTCTTTATCAGCTACCGGAGGAACTCAGGGTCCCAGTTGGCCAGCCTCCTGAAGGTGCACCTGCAGCTTCATGGCTTCAGCGTCTTCATCGACGTGGAGAAGCTGGAAGCCGGCAAATTTGAGGACAAGCTTATCCAAAGCGTCATGGCGGCTCGCAATTTTGTCCTGGTGCTGTCTGCTGGGGCGCTGGATAAGTGCATGCAGGACCATGAATGTAAGGACTGGGTGCACAAGGAGATTGTGACTGCTTTAAGCTGTGGCAAGAACATTGTGCCCATCATTGATGGCTTTGAGTGGCCTGAGCCTCAGGCGCTGCCTGAGGATATGCAGGCTGTACTCACCTTCAACGGCATCAAATGGTCCCATGAGTACCAGGAGGCCACCATCGAGAAGATCATCCGCTTCCTGCAGGGCCGTCCCTCTCAGGACTCCTCTGCCGGCTCGGATACCAGTTTGGAGGGAGCTACGCCAATGGGTCTGCCTTAA

>Mus-musculus

ATGGTCCTGACGCTGCTCTTCTCCGCCTACAAACTGTGCCGCTTCTTCACCATGTCAGGCCCACGGCCGGGCGCCGATCGGCTGACAGTGCCCGGACCGGATCGGAGTGGTGGCGCCAGCCCATGGTGGGCTGCGGGCGGTCGCGGGTCTCGCGAAGTGTCGCCCGGAGTGGGCACTGAGGTGCAAGGCGCCCTGGAGCGTTCGCTGCCTGAGCTGCAGCAGGCGCTGTCCGAGCTGAAACAGGCAAGCGCGGCGCGGGCTGTGGGCGCGGGTCTCGCCGAGGTCTTCCAGCTGGTAGAGGAAGCCTGGCTGCTGCCGGCCGTGGGCCGCGAGGTGGCCCAAGGTCTATGCGATGCTATACGTCTGGACGGTGGCCTCGACTTGCTGTTGCGGCTGCTTCAGGCACCGGAGCTAGAGACCCGTGTGCAGGCCGCGCGCTTGCTGGAGCAGATCCTGGTGGCTGAGAACCGGGACCGCGTGGCGCGCATCGGTCTAGGCGTGATCTTGAACCTGGCGAAGGAGCGCGAGCCTGTGGAACTGGCACGAAGCGTGGCGGGCATCTTGGAGCACATGTTCAAGCACTCGGAGGAGACGTGCCAGCGGCTGGTGGCGGCCGGAGGCCTCGACGCGGTGCTGTACTGGTGCCGCCGCACAGACCCGGCGCTGCTGCGCCACTGCGCTCTTGCGCTGGCGAACTGCGCGCTGCACGGGGGCCAGACGGTGCAGCGGTGCATGGTGGAGAAGCGCGCCGCCGAGTGGCTCTTCCCGCTCGCTTTCTCCAAGGAGGACGAGCTGCTGCGGCTGCACGCCTGCCTGGCGGTGGCGGTGTTGGCTACCAACAAGGAGGTGGAACGCGAGGTCGAGCATTCTGGCACATTGGCGCTTGTCGAGCCGCTCGTGGCATCGCTGGACCCCGGCCGCTTCGCCCGCTGCCTGGTGGATGCCAGTGACACAAGCCAGGGTCGTGGACCAGACGACCTGCAGAGCCTGGTGCTGTTGCTCGATTCGTCGCGTTTGGAGGCTCAGTGCATAGGAGCATTCTACCTGTGCGCAGAGGCTGCCATCAAGAGCCTACAGGGAAAGACCAAGGTGTTCAGCGACATCGGCGCTATCCAGAGCCTGAAACGCCTGGTTTCTTACTCTACGAATGGCACCACGTCGGCGCTGGCCAAGCGCGCGCTGCGCCTATTGGGCGAGGAGGTGCCAAGGCGCATCCTGCCCTGCGTGGCCAGCTGGAAGGAAGCTGAGGTCCAGACCTGGCTACAGCAGATCGGCTTCTCCCAGTACTGCGAGAACTTTCGGGAGCAGCAGGTAGATGGTGACCTGCTTCTAAGACTCACAGATGAAGAACTCCAGACAGACCTAGGCATGAAATCAAGCATCACCCGCAAGAGGTTCTTTAGGGAGCTCACAGAGCTCAAGACCTTCGCCAGCTACGCTACTTGCGACCGCAGCAACCTAGCGGACTGGCTGGGCAGCCTGGATCCTCGCTTCCGCCAGTACACCTATGGCCTGGTCAGCTGCGGTCTGGACCGCTCCCTGCTGCACCGCGTGTCAGAGCAGCAGCTCCTGGAGGACTGTGGCATCCGCCTGGGAGTGCACCGCACGCGCATCCTCTCTGCAGCCAGAGAAATGCTACATTCCCCGCTGCCCTGTACTGGAGGCAAGCTCAGTGGGGACACCCCAGATGTCTTTATCAGTTACCGGAGGAACTCAGGGTCCCAGCTGGCCAGCCTCCTGAAGGTGCACCTGCAGCTTCACGGCTTCAGCGTCTTCATCGACGTGGAGAAGCTGGAAGCCGGCAAATTCGAGGACAAGCTTATCCAAAGCGTCATAGCGGCTCGCAATTTTGTCCTGGTGCTGTCTGCTGGGGCGCTGGATAAGTGCATGCAGGACCATGACTGCAAGGACTGGGTGCACAAGGAGATTGTGACTGCTTTAAGCTGTGGCAAGAACATTGTGCCCATCATTGATGGCTTTGAGTGGCCTGAGCCTCAGGCGCTGCCTGAGGATATGCAGGCTGTACTCACCTTCAACGGCATCAAATGGTCCCATGAGTACCAGGAGGCCACCATCGAGAAGATCATCCGCTTCCTACAGGGCCGCCCCTCTCAGGACTCCTCTGCCGGATCGGATACCAGTTTGGAGGGAGCTACGCCAATGGGTCTGCCTTAA

>Mus-pahari

ATGGTCCTGACTCTGCTCTTCTCTGCCTACAAACTGTGCCGCTTCTTCACCATGTCAGGCCCACGACCGGGCGCCGATCGGCTGACAGTGCCCGGATCGGATCGGAGTGGTGGCGCCAGCCCATGGTGGGCTGCGGGCGGTCGCGGGTCTCGCGAAGTGTCGCCCGGGGTGGGCACTGAGGTGCAAGGCGCCCTGGAGCGTTCGCTGCCGGAGCTGCAGCAGGCGCTGTCCCAGCTGAAACAGGCAAGCGCGGCGCGGGCTGTGGGCGCGGGCCTCGCCGAGGTCTTCCAGCTGGTAGAGGAAGCCTGGCTGCTGCCGGCCGTGGGCCGCGAGGTGGCCCAGGGTCTATGCGATGCTATACGTCTGGACGGTGGCCTCGACTTGCTGTTGCGGCTGCTTCAGGCACCGGAGCTAGAGACCCGTGTGCAGGCCGCGCGCTTACTGGAGCAGATCCTGGTGGCTGAGAACCGGGACCGCGTGGCGCGCATCGGCCTAGGTGTGATCTTGAACCTGGCGAAGGAGCGCGAGCCTGTGGAGCTGGCACGGAGCGTGGCGGGCATCTTGGAGCACATGTTCAAGCACTCGGAGGAGACGTGCCAGCGGCTGGTGGCGGCCGGAGGCCTAGACGCGGTGCTGTACTGGTGCCGCCGCACAGACCCGGCGCTGCTGCGCCACTGCGCGCTGGCGCTGGCGAACTGCGCGCTGCACGGGGGGCAGACGGTGCAGCGGTGCATGGTAGAGAAGCGCGCCGCCGAGTGGCTCTTCCCGCTCGCTTTCTCCAAGGAGGACGAGCTGCTGAGGCTGCACGCCTGCCTGGCGGTGGCGGTGTTGGCTACCAACAAGGAAGTGGAACGCGAGGTCGAGCACTCCGGCACGTTGGCGCTTGTCGAGCCGCTCGTGGCATCGCTGGACCCCGGCCGCTTCGCCCGCTGCCTGGTGGATGCCAGTGACACAAGCCAGGGTCGTGGACCAGACGACCTGCAGAGCCTGGTGCTGTTGCTCGATTCGTCGCGTTTGGAGGCTCAGTGCATAGGGGCATTCTACCTGTGCGCTGAGGCTGCCATCAAGAGCCTACAGGGAAAGACCAAGGTGTTCAGCGACATCGGCGCTATCCAGAGCTTGAAACGCCTGGTTTCTTACTCTACAAATGGCACCACGTCGGCGCTGGCCAAGCGCGCGCTGCGCCTATTGGGCGAGGAGGTGCCAAGGCGCATCCTGCCCTGCGTGGCCAGCTGGAAGGAGGCTGAGGTCCAGACCTGGCTACAGCAGATCGGCTTCTCCCAGTACTGCGAGAACTTTCGGGAGCAGCAGGTAGATGGTGACCTGCTTCTAAGACTCACAGATGAAGAACTCCAGACAGACCTAGGCATGAAATCAAGCATCACCCGCAAGAGGTTCTTTAGGGAGCTCACGGAGCTCAAGACCTTCGCCAACTATGCCACTTGCGACCGCAGCAACCTAGCGGACTGGCTGGGCAGCCTGGATCCTCGCTTCCGCCAGTACACCTATGGCCTGGTCAGCTGCGGCCTGGACCGCTCCCTGCTGCACCGCGTATCAGAGCAGCAGCTCCTGGAGGACTGTGGCATCCGCCTGGGGGTGCACCGCACGCGCATCCTCTCTGCAGCCAGAGAAATGTTACATTCCCCGCTGCCCTGTACTGGAGGCAAACCCAGTGGAGACACCCCAGATGTCTTTATCAGTTACCGGAGGAACTCTGGTTCCCAGCTGGCCAGCCTCCTGAAGGTACACCTGCAGCTGCACGGCTTCAGCGTATTCATCGACGTGGAGAAGCTGGAAGCCGGCAAATTCGAGGACAAGCTCATCCAAAGTGTCATGGCGGCTCGCAATTTTGTACTGGTGCTGTCTGCTGGGGCGCTGGATAAGTGCATGCAGGACCATGACTGCAAGGACTGGGTGCACAAGGAGATCGTGACTGCTTTAAGCTGTGGCAAGAACATTGTGCCCATCATTGATGGCTTTGAGTGGCCTGAGCCTCAGGCGCTTCCTGAGGATATGCAGGCTGTACTCACCTTCAATGGCATCAAATGGTCCCATGAGTACCAGGAGGCCACCATCGAGAAGATTATCCGCTTCCTACAGGGCCGCCCCTCTCAGGACTCCTCTGCCGGCTCGGATACCAGTTTGGAGGGAGCTACGCCAATGGGTCTGCCTTAA

>Rattus-rattus

ATGGTCCTGACGCTGCTCTTCTCCGCCTACAAACTGTGCCGCTTCTTCATCATGTCAGGCCCACGGCCGGGCGCCGATCGGCTGACAGTACCGGGACCGGATCGGAGTGGCGGCACCAGCCCATGGTGGGCTGCGGGCGGTCGCGGGTCTCGCGAAGTGTCGCCCGGGGTGGGCACTGAGGTGCAAAGCGCCCTGGAGCGTTCGCTGCCGGAGCTGCAGCAGGCGCTGTCCGAGCTGAAACAGGCAAGCGCGGCGCGGGCGGTGGGCGCGGGCCTCGCCGAGGTCTTCCAGCTGGTAGAGGAAGCCTGGCTGCTGCCGGCCGTGGGCCGCGAGGTGGCCCAAGGTTTGTGCGATGCTATACGTCTGGACGGTGGCCTCGACTTGCTGTTGCGGCTGCTTCAGGCACCGGAGCTAGAGACCCGCGTGCAGGCCGCGCGCTTGTTGGAGCAGATCCTGGTGGCTGAGAACCGGGACCGCGTAGCGCGCATCGGCCTAGGCGTGATCTTGAACCTGGCGAAGGAACGCGAGCCTGTGGAGCTGGCACGGAGTGTGGCGGGCATCTTGGAGCACATGTTCAAGCACTCGGAGGAGACCTGCCAGCGGCTGGTGGCGGCCGGAGGCCTCGACGCGGTGCTGTATTGGTGCCGCCGCACAGACCCAGCGCTACTGCGCCACTGCGCGCTGGCGCTGGCGAACTGCGCGCTGCACGGGGGCCAGACTGTGCAGCGGTGCATGGTGGAGAAGCGCGCCGCCGAGTGGCTCTTCCCGCTCGCTTTCTCCAAAGAGGACGAGTTGCTGCGGCTGCATGCTTGCCTGGCGGTGGCCGTGTTGGCTACCAACAAGGAGGTAGAACGCGAGGTCGAGCACTCAGGCACGTTGGCGCTCGTCGAGCCGCTGGTGGCATCGCTGGATCCTGGCCGCTTCGCTCGTTGCCTGGTGGATGCCAGTGACACAAGCCAGGGTCGTGGACCAGACGACCTGCAGAGCCTGGTGCTGTTGCTCGATTCGTCGCGTTTGGAGGCTCAGTGCATAGGGGCTTTCTACCTGTGCGCCGAGGCTGCCATCAAAAGCCTACAGGGAAAGACCAAGGTGTTCAGCGACATTGGCGCCATTCAGAGCCTGAAACGCCTGGTTTCTTACTCCACAAATGGCACCACGTCGGCGCTGGCCAAGCGCGCGCTGCGCCTATTGGGCGAGGAGGTACCAAGGCGCATCCTACCCTGCGTGGCCAGCTGGAAGGAGGCTGAGGTCCAGACCTGGCTGCAGCAGATCGGCTACTCCCAGTACTGCGAGAACTTTCGGGATCAGCAGGTAGATGGTGATTTGCTTCTACGACTCACAGATGAAGAACTCCAGACAGACCTAGGCATGAAATCAAGCATCACTCGCAAGAGGTTCTTTAGGGAGCTCACTGAGCTCAAGACCTTCGCCAGCTATGCTACTTGCGACCGCAGCAACCTAGCAGACTGGCTGGGAAGCCTGGATCCTCGCTTCCGCCAGTACACCTATGGCCTGGTCAGCTGCGGCCTGGACCGCTCCCTGCTGCACCGCGTGTCAGAGCAGCAGCTCCTGGAGGACTGTGGCATCCGCCTGGGGGTGCATCGCACGCGCATCCTCTCTGCAGCCAGAGAAATGCTACATTCCCCGCTGCCCTGTACCGGAGGCAAGCCCAGTGGAGACACCCCAGATGTCTTTATCAGCTACCGCAGGAACTCTGGTTCCCAGCTGGCCAGCCTCCTGAAGGTGCACCTGCAGCTGCATGGCTTCAGTGTCTTCATCGATGTGGAGAAGCTGGAGGCTGGCAAATTCGAGGACAAGCTCATCCAGAGTGTTATGGCGGCTCGAAATTTTGTGCTGGTGCTGTCTGCGGGAGCGCTGGATAAGTGCATGCAGGACCATGACTGCAAGGACTGGGTGCACAAGGAGATCGTGACTGCCTTAAGCTGTAGCAAGAACATTGTGCCCATCATTGATGGCTTTGAGTGGCCTGAGCCTCAGGCACTGCCTGAGGATATGCAGGCTGTACTCACCTTCAACGGCATCAAATGGTCTCATGAGTACCAGGAGGCCACCATCGAGAAAATCATCCGCTTCCTACAGGGTCGCCCCTCTCAGGACTCCTCTGCCGGCTCTGACACCAGTTTGGAGGGAGCTACATCAATGGGTCTGCCTTAA

>Acomys_russatus

ATGGTCCTGACGCTGCTCTTCTCCGCCTACAAACTGTGCCGCTTCTTCACCATGTCAGGCCCACGGCCCGGCGCCGATCGGCTGACAGTGCCAGGACAGGATCGCACTGGCGGCGCCAGCCCATGGTGGGCTGCGGGCGGCCGCGGGTCCCGCGAAGTGTCTCCCGGGGTGGGCACCGAAGTGCAAGGAGCCCTGGAGCGTTCGCTACCGGAGCTGCAGCAGGTGCTTTCCGAGTTGAAGCAGGCAAGCGCGGCGCGGGCGGTGGGCGCGGGCCTCGCTGAGGTCTTCCAGCTGGTAGAGGAGGCCTGGCTGCTGCCGGCTGTGGGCCGCGAGGTGGCCCAGGGTCTGTGCGATGCAATACGCTTGGACGGTGGCCTGGACTTGCTGTTGCGGCTGCTGCAGGCACCAGAGCTAGAGACCCGTGTGCAGGCCGCGCGCTTGCTGGAGCAGATCCTGGTGGCTGAGAACCGGGACCGCGTGGCGCGCATCGGCCTAGGCGTGATCTTGAATCTGGCGAAGGAGCGCGAGCCGGTGGAGCTGGCACGGAGCGTGGCGGGCATCTTGGAGCACATGTTCAAGCACTCGGAGGAGACGTGCCAGCGGTTGGTGGCAGCCGGTGGCCTGGACGCGGTGCTGTACTGGTGCCGACGAACAGACCCGGCGCTGCTGCGCCACTGCGCTCTGGCGTTAGCAAACTGCGCGCTGCATGGGGGCCAGGCGGTGCAGCGGTGCATGGTGGAGAAGCGCGCCGCCGAGTGGCTCTTCCCGCTCGCCTTCTCCAAGGAGGACGAGCTGCTGCGGCTGCACGCCTGCCTGGCGGTGGCGGTGTTGGCTACTAACAAGGAGGTGGAGCGCGAAGTCGAACACTCGGGCACGTTGGCGCTCGTCGAGCCGCTCGTGGCATCGCTGGACCCCGGCCGCTTCGCCCGCTGCCTGGTGGATGCCAGTGACACAAGCCAGGGTCGCGGGCCGGAGGACCTGCAGAGCCTGGTGCTGCTGCTGGATTCGTCGCGTTTGGAGGCTCAGTGCATCGGGGCTTTCTACCTGTGTGCCGAGGCTGCTATCAAGAGCCTACAGGGAAAGACTAAGGTGTTCAGCGACATCGGCGCCATCCAGAGCCTGAAACGCCTGGTTTCTTACTCCACTAATGGCACCACGTCGGCGCTGGCGAAGCGCGCGCTGCGCCTGTTGGGCGAGGAGGTGCCGAGGCGCATCTTGCCCTGCGTGGCCAGCTGGAAGGAGGCAGAGGTCCAGACCTGGCTGCAGCAGATCGGCTTCTCCCAGTACTGCGAGAACTTTCGGGATCAGCAGGTAGATGGTGACCTGCTTCTACGACTCACAGAGGAAGAACTCAGGACAGACCTGGGCATGAAATCAAGCATCACACGCAAGAGGTTCTTTAGGGAGCTCACGGAGCTCAAGACCTTTGCCAGCTATGCTACTTGCGACCGCAGCAACCTAGCAGACTGGCTGGGCAGCCTGGACCCTCGCTTCCGTCAGTACACCTACGGCCTGGTGAGCTGCGGCCTGGACCGCTCCCTGCTGCACCGCGTGTCGGAGCAGCAGCTGCTGGAGGACTGTGGTATCCGCCTAGGGGTGCATCGCACGCGCATCCTCTCGGCAGCCAGAGAAATGCTCCACTCCCCACTGCCCTGTAGCGGTGGCAAACCCAGCGGAGACACCCCAGACGTCTTCATCAGTTACCGGAGGAACTCGGGTTCCCAGCTGGCCAGCCTCCTGAAGGTGCATCTGCAGCTCCATGGCTTCAGCGTCTTCATCGACGTGGAGAAGCTGGAGGCAGGCAAGTTTGAGGACAAGCTCATCCAGAGTGTCATGGCGGCTCGAAACTTCGTACTGGTGCTGTCCGCTGGAGCACTGGACAAGTGCATGCAGGACCATGACTGCAAGGACTGGGTGCACAAGGAGATCGTGACTGCTTTAAGCTGTGCCAAGAACATTGTGCCCATCATTGATGGCTTCGAGTGGCCCGAGCCTCAGGCCCTGCCTGAGGACATGCAGGCTGTACTCACCTTCAATGGCATCAAATGGTCCCACGAGTACCAGGAGGCCACCATTGAGAAGATCATCCGTTTCCTGCAGGGCCGCCCCTCTCAAGACTCCTCTGCCGGCTCTGACACCAGTTTGGAGGGAGCTGCACCAATGGGTCTGCCTTAA

>Meriones-unguiculatus

ATGGTCCTGACGCTGCTCTTCTCCGCCTACAAACTGTGCCGCTTCTTCACCATGTCGGGCCCACGGCCGGGCGCCGATCGGCTGACAGTGCCGGGCCCAGATCGGAGTGGCGGCGCCAGCCCCTGGTGGGCTGCGGGCGGCCGCGGGTCCCGCGAGGTGTCGCCAGGGGTGGGGACCGAGGTACTAGGAGCCCTGGAGCGTTCGCTGCCGGAGCTGCAGCAGGCGCTGTCCCAGTTAAAGCAGGCGAGCGCGGCGCGGGCGGTGGGCGCGGGCCTCGCCGAGGTCTTCCAGCTGGTGGAGGAGGCCTGGCTGCTGCCCGCTGTGGGCCGCGAGGTGGCCCAGGGTCTGTGCGATGCGATCCGCCTGGACGGGGGCCTGGACTTGCTGCTGCGGCTGCTGCAGGCACCGGAGCTGGAGACCCGCGTGCAGGCCGCGCGCTTGCTGGAGCAGATCCTGGTGGCTGAGAACCGGGACCGCGTGGCGCGCATCGGCCTAGGCGTGATCTTGAACCTGGCGAAGGAGCGCGAGCCGGTGGAGCTGGCGCGGAGCGTGGCGGGCATCTTGGAGCATATGTTCAAGCATTCGGAGGAGACGTGCCAGCGGCTGGTGGCGGCCGGCGGCCTGGACGCGGTGCTGTACTGGTGCCGCCGCACAGACCCGGCGCTCCTGCGCCACTGCGCTCTGGCGCTGGCCAACTGCGCGCTGCACGGGGGCCAGACGGTGCAGCGGTGCATGGTGGAGAAGCGCGCTGCCGAGTGGCTCTTCCCGCTCGCCTTCTCCAAGGAGGACGAGCTGCTGCGGCTGCACGCCTGCCTGGCGGTGGCCGTGTTGGCTACCAACAAGGAGGTGGAGCGCGAGGTCGAGCACTCCGGCACTCTGGCGCTCGTGGAGCCGCTCGTGGCATCGCTTGATCCCGGTCGCTTCGCTCGCTGCCTCGTGGACGCCAGCGACACAAGCCAGGGTCGCGGCCCGGAGGACCTGCAGAGCCTGGTGCTGCTGCTCGATTCGTCGCGTTTGGAGGCTCAGTGCATAGGGGCCTTTTACCTGTGCGCCGAGGCTGCCATCAAGAGCCTACAGGGGAAGACCAAGGTGTTCAGCGACATCGGCGCCATCCAGAGCCTGAAGCGCCTGGTTTCTTACTCCACCAACGGCACCACTTCGGCGCTGGCGAAGCGCGCGCTGCGCTTGCTGGGCGAGGAGGTGCCGAGGCGCATCCTGCCCTGCGTGGCCAGCTGGAAGGAGGCCGAGGTCCAGACGTGGCTGCAGCAGATCGGCTTCTCCCAGTACTGCGAGAACTTCCGGGAACAGCAAGTGGACGGTGACCTGCTTCTGCGGCTCACAGAGGAAGAACTGCAGACAGACCTGGGCATGAAGTCAAGCATCACCCGCAAGAGGTTCTTTAGGGAGCTCACGGAGCTCAAGACCTTCGCCAGCTACGCCACCTGCGACCGCAGCAACCTCGCAGACTGGCTGGGCAGCCTGGACCCTCGCTTCCGCCAGTACACCTACGGGCTGGTCAGCTGTGGCCTGGACCGCTCCCTGCTGCACCGCGTGTCCGAGCAGCAGCTCCTGGAGGACTGCGGCATCCGCCTGGGGGTGCATCGCACGCGAATCCTCTCTGCAGCCAGAGAAACGCTCCACTCCCCGGTGCCCTGTACCAGTGGCAAGTCCAGCGGGGACACCCCGGATGTCTTCATCAGCTACCGGAGGAGCTCGGGTTCCCAGCTGGCCAGCCTCCTGAAGGTGCACCTGCAGCTGCACGGCTTCAGCGTCTTCATCGACGTGGAGAAGCTGGAAGCTGGCAAGTTTGAGGACAAGCTCATCCAGAGCGTCATGGCCGCTCGGAACTTTGTCTTGGTGCTCTCCGCCGGGGCGCTGGACAAGTGCATGCAGGACCATGAGTGCAAGGACTGGGTACACAAGGAGATTGTGACTGCTTTAAGCTGTGCCAAGAACATTGTCCCCATCATTGATGGCTTCGAGTGGCCGGAGCCTCAGTCACTGCCTGAGGACATGCAGGCTGTACTCACCTTCAATGGCATCAAATGGTCTCACGAGTACCAGGAGGCCACCATCGAGAAGATCATCCGTTTCCTGCAGGGCCGCCCCTCTCAGGACTCCTCTGCAGGCTCCGATACCAGCTTGGAGGGAGCTGCACCAATGGGTCTGCCTTAA

>Psammomys_obesus

ATGGTCCTGACGCTGCTCTTCTCCGCCTACAAACTGTGCCGCTTCTTCACCATGTCGGGCCCACGGCCGGGCGCCGATCGGCTGACGGTGCCGGGGCCGGATCGGAGTGGCAGCGCCAGCCCCTGGTGGGCTGCGGGCGGCCGCGGGTCCCGCGAGGTGTCGCCAGGGGTGGGGACCGAGGTGCAAGGAGCCCTGGAGCGTTCGCTGCCGGAGCTGCAGCAGGCGCTGTCCCAGTTAAAGCAGGCGAGCGCGGCGCGGGCGGTGGGCGCGGGCCTCGCCGAGGTCTTCCAGCTGGTAGAGGAGGCCTGGCTGCTGCCCGCTGTGGGCCGCGAGGTGGCCCAGGGTCTGTGCGATGCGATACGCCTGGACGGGGGCCTGGACTTGCTGCTGCGGCTGCTGCAGGCGCCGGAGCTGGAGACCCGCGTGCAGGCCGCGCGCTTGCTGGAGCAGATCCTGGTGGCTGAGAACCGGGACCGCGTGGCGCGCATCGGCCTAGGCGTGATCTTGAACCTGGCGAAGGAGCGCGAGCCGGTGGAGCTGGCGCGGAGCGTGGCGGGCATCTTGGAGCACATGTTCAAGCATTCGGAGGAGACGTGCCAGCGGCTGGTGGCGGCCGGCGGCCTGGACGCGGTGCTGTACTGGTGCCGCCGCACAGACCCGGCGCTCCTGCGCCACTGCGCTCTGGCGCTGGCCAACTGCGCGCTGCACGGGGGCCAGACGGTGCAGCGGTGCATGGTGGAGAAGCGTGCTGCCGAGTGGCTCTTCCCGCTCGCCTTCTCCAAGGAGGACGAGCTGCTGCGGCTGCACGCCTGCCTGGCGGTGGCCGTGTTGGCTACCAACAAGGAGGTGGAGCGCGAGGTCGAGCACTCCGGCACTCTGGCGCTCGTGGAGCCGCTCGTGGCATCCCTGGATCCCGGTCGCTTCGCTCGCTGCCTCGTGGACGCCAGCGACACAAGCCAGGGTCGCGGCCCGGAGGACCTGCAGAGCCTGGTGCTGCTGCTCGATTCGTCGCGTTTGGAGGCTCAGTGCATAGGGGCTTTTTACCTGTGCGCCGAGGCTGCCATCAAGAGCCTACAGGGGAAGACCAAGGTGTTCAGCGACATCGGCGCCATCCAGAGCCTGAAGCGCCTGGTTTCTTACTCCACCAACGGCACCACGTCGGCGCTGGCGAAGCGCGCGCTGCGGCTGCTGGGCGAGGAGGTGCCGAGGCGCATCCTGCCCTGCGTGGCCAGCTGGAAGGAGGCCGAGGTCCAGACGTGGCTGCAGCAGATCGGCTTCTCCCAGTACTGCGAGAACTTCCGGGAACAGCAGGTAGACGGTGACCTGCTTCTGCGACTCACAGAGGAGGAACTGCAGACAGACCTGGGCATGAAGTCAAGCATCACCCGCAAGAGGTTCTTTAGGGAGCTCACGGAGCTCAAGACCTTCGCCAGCTACGCCACCTGCGACCGCAGCAACCTCGCAGACTGGCTGGGCAGCCTGGACCCTCGCTTCCGCCAGTACACCTACGGGCTGGTCAGCTGTGGCCTGGACCGCTCCCTGCTGCACCGCGTGTCCGAGCAGCAGCTCCTGGAGGACTGCGGCATCCGCCTGGGGGTGCATCGCACGCGAATCCTCTCTGCAGCCAGAGAAACGCTCCACTCCCCGGTGCCCTGTACCAGTGGCAAGTCCAGCGGGGACACCCCAGATGTCTTCATCAGCTACCGGAGGAGCTCGGGTTCCCAGCTGGCCAGCCTCCTGAAGGTGCACCTGCAGCTGCATGGCTTCAGCGTCTTCATCGACGTGGAGAAGCTGGAAGCTGGCAAGTTCGAGGACAAGCTCATCCAGAGTGTCATGGCCGCTCGGAACTTTGTCCTGGTGCTCTCCGCCGGGGCGCTGGACAAGTGCATGCAGGACCATGAGTGCAAGGACTGGGTACACAAGGAGATTGTGACTGCTTTAAGCTGTGCCAAGAACATTGTCCCCATCATTGATGGCTTTGAGTGGCCGGAGCCTCAGTCACTGCCTGAGGACATGCAGGCTGTACTCACCTTCAATGGCATCAAATGGTCTCACGAGTACCAGGAGGCCACCATCGAGAAGATCATCCGTTTCCTGCAGGGCCGCCCCTCTCAGGACTCCTCTGCGGGCTCTGATACCAGCTTGGAGGGAGCTGCACCAATGGGTCTGCCTTAA

Supplementary Data 4. Aligned sequences of TRAM1

>Rattus norvegicus

ATGGCGATTCGCAAGAAGAGCAACAAGAACCCGCCGGTGTTGAGCCATGAATTCGTGCTGCAGAATCACGCGGACATCGTCTCCTGTCTGGCGATGCTCTTCCTGCTGGGGCTTATGTTCGAGATAACAGCAAAAGGAGCCATCATCTTTGTCGCCCTTCAGTACAACGTTACCCGCCCTGCAACAGAAGAACAAGCTGCTGAATCAGCATCCCTTTATTACTATGGTATCAAAGATTTGGCTACAGTGTTCTTCTACATGCTAGTGGCGATAATTGTTCATGCCATAATTCAGGAGTATGTGTTGGATAAAATTAACCGACGGATGCACTTCTCCAAAACGAAGCACAGCAAGTTTAATGAGTCCGGGCAGCTCAGTGCGTTCTACCTTTTTGCCTGTGTGTGGGGCACATTCATTCTCGTCTCCGAAAACTATATCTCAGACCCGACGATCCTGTGGAGAGCGTATCCCCATAACCTGATGACATTTCAAATGAAGTTTTTCTACATATCCCAGTTGGCGTATTGGCTCCATGCATTCCCTGAACTCTACTTCCAGAAAACCAAAAAAGAAGATATTCCTCGCCAGCTTGTCTACATCGGTCTTTACCTCTTCCACATTGCTGGGGCTTACCTTCTGAACTTGAACCATCTGGGCCTTGTTCTTCTGGTGCTGCACTATTTCGTTGAATTTCTTTTCCACATTTCTCGTCTATTTTATTTTAGTGACGAGAAGTACCAGAAAGGGTTTTCTCTTTGGGCGGTTCTTTTTGTTTTGGGAAGACTTCTGACCCTAATTCTTTCAGTCCTCACTGTTGGCTTTGGCCTGGCAAGAGCAGAGAACCAGAAGCTGGACTTCAGTACTGGGAACTTCAATGTGTTGGCGGTTAGGATCGCGGTCCTAGCGTCCATTTGCATCACTCAAGCCTTCATGATGTGGAAGTTCATTAACTTCCAGCTTCGGAGGTGGAGGGAACATTCTGCCTTCCAGGCCCCTCCCGTGAAAAGGAAACCGGCTGTGACCAAAGGCAGGTCTTCCAGAAAAGGAACAGAGAATGGTGTGAACGGCACGGTCACGTCGAATGGAGCCGACTCTCCTCGGAGCAGGAAAGAGAAGTCTTCCTAA

>Rattus tanezumi

ATGGCGATTCGCAAGAAGAGCAACAAGAACCCGCCGGTGTTGAGCCATGAATTCGTGCTGCAGAATCACGCGGACATCGTCTCCTGTCTGGCGATGCTCTTCCTGCTGGGGCTTATGTTCGAGATAACAGCAAAAGGAGCCATCATCTTTGTCGCCCTTCAGTACAACGTTACCCGCCCTGCAACAGAAGAACAAGCTGCTGAATCAGCATCCCTTTATTACTATGGTATCAAAGATTTGGCTACAGTGTTCTTCTACATGCTAGTGGCGATAATTGTTCATGCCATAATTCAGGAGTATGTGTTGGATAAAATTAACCGACGGATGCACTTCTCCAAAACGAAGCACAGCAAGTTTAATGAGTCCGGGCAGCTCAGTGCGTTCTACCTTTTTGCCTGTGTGTGGGGCACATTCATTCTCGTCTCCGAAAACTATATCTCAGACCCGACGATCCTGTGGAGAGCGTATCCCCATAACCTGATGACATTTCAAATGAAGTTTTTCTACATATCCCAGTTGGCGTATTGGCTCCATGCATTCCCTGAACTCTACTTCCAGAAAACCAAAAAAGAAGATATTCCTCGCCAGCTTGTCTACATCGGTCTTTACCTCTTCCACATTGCTGGGGCTTACCTTCTGAACTTGAACCATCTGGGCCTTGTTCTTCTGGTGCTGCACTATTTCGTTGAATTTCTTTTCCACATTTCTCGTCTATTTTATTTTAGTGACGAGAAGTACCAGAAAGGGTTTTCTCTTTGGGCGGTTCTTTTTGTTTTGGGAAGACTTCTGACCCTAATTCTTTCAGTCCTCACTGTTGGCTTTGGCCTGGCAAGAGCAGAGAACCAGAAGCTGGACTTCAGTACCGGGAACTTCAATGTGTTGGCGGTTAGGATCGCGGTCCTGGCGTCCATTTGCATCACTCAAGCCTTCATGATGTGGAAGTTCATTAACTTCCAGCTTCGGAGGTGGAGGGAACACTCTGCCTTCCAGGCCCCTCCCGTGAAAAGGAAACCGGCTGTGACCAAAGGCAGGTCTTCCAGAAAAGGAACAGAGAATGGTGTGAACGGCACGGTCACGTCGAATGGAGCCGACTCTCCTCGGAGCAGGAAAGAGAAGTCTTCCTAA

>Acomys_russatus

ATGGCGATTCGCAAGAAGAGCAACAAGAACCCGCCGCTGCTGAGCCACGAATTCGTGCTGCAGAATCACGCGGACATCGTCTCCTGCCTGGCGATGCTCTTCCTGCTGGGGCTCATGTTCGAGGTAACAGCAAAAGCCGCCATCATCTTTGTCGCTCTTCAGTACAATGTTACCCGCCCTGTGACAGAAGAACAAGCTACTGAATCAGCATCCCTTTATTACTATGGTATCAAAGATTTGGCTACCGTTTTCTTCTACATGCTCGTGGCGATAATTATTCATGCCATAATTCAGGAGTACGTGTTGGATAAAATAAACAGACGAATGCACTTCTCCAAAACAAAGCACAGCAAGTTTAATGAGTCTGGTCAACTTAGTGCATTCTACCTTTTGGCTTGTGTGTGGGGCACATTCATTCTCATCTCTGAAAACTACATCTCAGATCCAACTATCCTGTGGAGAGCTTATCCCCATAACCTGATGACATTTCAAATGAAGTTTTTCTACATATCGCAGTTGGCGTACTGGCTCCATGCGTTCCCTGAACTCTACTTCCAGAAAACCAAAAAAGAAGATATTCCTCGTCAGCTTGTCTACATTGGTCTTTACCTCTTCCACATCGCTGGAGCGTATCTTTTGAACTTGAACCACCTGGGCCTTGTTCTTCTGGTGCTGCACTATTTCGTTGAGTTTCTGTTCCACATTTCTCGTCTGTTTTACTTTAGTGATGAGAAGTACCAGAAGGGGTTTTCTCTCTGGGCGGTTCTTTTTGTTTTGGGAAGACTTCTGACCTTAATTCTTTCAGTCCTTACCGTTGGTTTCGGCTTGGCAAGAGCGGAGAATCAGAAGCTGGATTTTAGTACTGGAAACTTCAATGTGTTGGCGGTTAGGGTTGCTGTTCTGGCGTCCATTTGCGTCACGCAGGCCTTCATGATGTGGAAGTTCATTAACTTCCAGCTTCGGAGGTGGAGGGAGCACTCTGCCTTCCAGGCGCCGCCTGTGAAGAGGAAACCAGCCGTGACCAAAGGCAGGTCTTCCCGGAAAGGAACAGAGAACGGTGTGAATGGAACAGTCACCTCTAACGGAGCTGACTCTCCCCGGAACAGGAAAGAGAAGTCTTCATAA

>Apodemus-sylvaticus

ATGGCGATTCGCAAGAAGAGCAACAAGAACCCGCCGCTGATGAGCCACGAATTCGTGCTGCAGAATCACGCGGACATCGTCTCCTGCCTGGCGATGCTCTTCCTGCTGGGGCTTATGTTCGAGATAACAGCGAAAGGAGCCATCATCTTTGTTGCTCTTCAGTACAATGTCACCCACCCTGCAACAGAAGAACAAGCTACTGAATCAGCATCCCTATATTACTATGGTATCAAAGATTTGGCTACAGTTTTCTTCTACATGCTAGTGGCGATAATTATTCATGCCATAATTCAGGAGTACGTGTTGGACAAAATTAACCGACGGATGCACTTCTCTAAAACGAAGCACAGCAAGTTTAACGAGTCTGGTCAGCTCAGTGCATTCTACCTTTTTGCCTGTGTGTGGGGCACGTTCATTCTCATCTCCGAAAACTATATCTCAGACCCAACTATCCTGTGGAGAGCATATCCCCATAACCTGATGACATTCCAGATGAAGTTTTTCTACATCTCCCAGCTGGCGTACTGGCTCCACGCGTTCCCTGAACTCTACTTCCAGAAAACCAAAAAGGAAGATATCCCTCGTCAGCTTGTCTACATCGGCCTTTACCTCTTCCATATTGCTGGAGCTTATCTTCTGAACTTGAATCACCTGGGCCTTGTTCTTCTGGTGCTGCACTATTTCGTTGAATTTCTTTTCCACATTTCTCGTCTATTTTATTTTAGTGACGAGAAGTATCAGAAAGGGTTTTCTCTCTGGGCGGTTCTTTTTGTTTTGGGAAGACTTCTTACCCTAATTCTTTCCGTTCTTACTGTTGGTTTTGGCCTGGCGAGAGCAGAGAATCAGAAGCTGGACTTTAGTAGTGGAAACTTCAACGTGCTGGCCGTTAGGATCGCTGTTCTCGCGTCCATTTGCATCACACAAGCCTTCATGATGTGGAAGTTCATTAACTTCCAGCTTCGGAGGTGGAGGGAACATTCTGCCTTCCAGGCCCCACCTGTGAAAAGGAAACCGGCTGTGACCAAAGGCAGGTCCTCCAGAAAAGGAACAGAGAACGGTGTGAACGGCACAGTCACATCAAATGGAGCGGATTCCCCGCGGAACAGGAAAGAGAAGTCTTCGTAA

>Arvicanthis-niloticus

ATGGCGATTCGCAAGAAGAGCAACAAGAACCCGCCACTGCTGAGCCACGAATTCGTGCTGCAGAATCACGCGGACATCGTCTCCTGCCTGGCGATGCTCTTCCTGCTGGGACTTATGTTCGAGATAACAGCAAAAGCAGCCATCATCTTTGTTGCGCTTCAGTATAATGTCACCCGCCCTGCAACAGAAGAACAAGCTACTGAATCAGCATCCCTTTATTACTATGGTATCAAAGATTTGGCTACAGTTTTCTTCTACATGCTAGTGGCGATAATTATTCATGCCATAATTCAGGAGTATGTGTTGGATAAAATTAACCGACGGATGCACTTCTCCAAAACGAAGCACAGCAAGTTTAACGAATCTGGTCAGCTCAGTGCATTCTACCTTTTTGCCTGTGTGTGGGGCACATTCATTCTCATCTCCGAAAACTATATCTCAGACCCGACTATTCTGTGGAGAGCGTATCCCCATAACCTGATGACATTTCAAATGAAGTTTTTCTACATCTCTCAGCTGGCGTACTGGCTCCATGCATTCCCTGAACTCTACTTCCAGAAAACCAAAAAAGAAGATATTCCTCGTCAGCTCGTCTACATTGGTCTTTACCTCTTTCACATTGCTGGAGCTTATCTTTTGAACTTGAACCATCTGGGCCTTGTTCTTCTGGTGCTGCACTATTTCGTTGAATTTCTTTTCCACATTTCTCGTCTATTTTATTTTAGTGACGAGAAGTATCAGAAAGGGTTTTCACTCTGGGCAGTTCTTTTTGTTTTGGGAAGACTTCTGACCCTAATTCTTTCAGTCCTTACTGTTGGTTTTGGCCTGGCAAGAGCGGAGAATCAGAAGCTGGATTTTAGTACTGGAAACTTCAATGTGTTGGCGGTTAGGATTGCTGTTCTAGCGTCCATTTGCATCACACAAGCCTTCATGATGTGGAAGTTCATTAACTTCCAGCTTCGGAGGTGGAGGGAACATTCTGCCTTTCAGGCCCCACCTGTGAAAAGGAAACCGGTCGTGACCAAAGGCAGGTCTTCCAGAAAAGGAACAGAGAACGGTGTGAACGGCACAGTCACTTCAAATGGAGCTGACTCTCCACGGAACAGGAAAGAGAAGTCTTCGTAA

>Grammomys-surdaster

ATGGCGATTCGCAAGAAGAGCAACAAGAACCCGCCACTGCTGAGCCACGAATTCGTGCTGCAGAATCACGCGGACATCGTCTCCTGCCTGGCGATGCTCTTCCTGCTGGGGCTTATGTTCGAGATAACAGCAAAAGCAGCCATCATCTTTGTTGCCCTTCAGTATAACGTTACCCGCCCCGCAACAGAAGAACAAGCTACTGAATCAGCATCCCTTTATTACTATGGTATCAAAGATTTGGCTACAGTTTTCTTCTACATGCTAGTGGCGATAATTATTCATGCCATAATTCAGGAGTATGTGTTGGATAAAATTAACCGACGGATGCACTTCTCCAAAACGAAGCACAGCAAGTTTAACGAATCTGGTCAGCTCAGTGCATTCTACCTTTTTGCCTGTGTGTGGGGCACATTCATTCTCATCTCCGAAAACTATATCTCAGACCCGACTATTCTGTGGAGAGCGTATCCCCATAACCTGATGACATTTCAAATGAAGTTTTTCTACATCTCTCAGCTGGCGTACTGGCTCCATGCATTCCCTGAACTCTACTTCCAGAAAACCAAAAAAGAAGATATCCCTCGTCAGCTCGTGTACATTGGTCTTTACCTCTTTCACATTGCTGGAGCTTATCTTTTGAACTTGAACCACCTGGGCCTTGTTCTTCTGGTGCTGCACTATTTCGTTGAATTTCTTTTCCACATTTCTCGTCTATTTTATTTTAGTGACGAGAAGTATCAGAAAGGGTTTTCTCTCTGGGCAGTTCTTTTTGTTTTGGGAAGACTTCTGACCCTAATTCTTTCAGTCCTTACTGTTGGTTTTGGGCTGGCAAGAGCGGAGAATCAAAAGCTGGATTTTAGTACTGGAAACTTCAACGTGTTGGCGGTTAGGATTGCTGTTCTGGCATCCATTTGCATCACACAAGCCTTCATGATGTGGAAGTTCATTAACTTCCAGCTTCGGAGGTGGAGGGAACATTCTGCCTTCCAGGCCCCACCTGTGAAACGGAAACCAGCCGTGACCAAAGGCAGGTCTTCCAGAAAAGGAACAGAGAACGGTGTGAATGGCACAGTCACTTCAAATGGAGCTGACTCTCCACGGAACAGGAAAGAGAAGTCTTCGTAA

>Mastomys-coucha

ATGGCGATTCGCAAGAAGAGCAACAAGAACCCGCCGTTGCTGAGCCACGAATTCGTGCTGCAGAATCACGCGGACATCGTCTCCTGCCTGGCGATGCTCTTCCTGCTGGGGCTTATGTTTGAGATAACAGCAAAAGGAGCCATCATCTTTGTTGCTCTTCAGTATAATGTTACCCACCCTGCAACAGAAGAACAAGCTACTGAATCAGCATCCCTATATCACTATGGTATCAAAGACTTGGCTACAGTGTTCTTCTACATGCTAGTGGCGATAATTATTCACGCCATAATTCAGGAGTATGTGTTGGATAAAATTAACCGACGGATGCACTTCTCCAAAACGAAGCACAGCAAGTTTAATGAATCTGGTCAGCTCAGTGCATTCTACCTTTTTGCCTGTGTGTGGGGCACATTCATTCTCATCTCTGAAAACTATATCTCAGACCCAACTATCCTGTGGAGAGCATATCCCCATAACCTGATGACCTTTCAAATGAAGTTTTTCTACATCTCCCAGCTGGCATACTGGCTCCATGCGTTCCCTGAACTCTACTTCCAGAAAACCAAAAAAGAAGATATTCCTCGTCAGCTCGTCTACATTGGTCTTTACCTTTTCCATATTGCAGGAGCTTATCTTTTGAACTTGAACCATCTGGGCCTTGTTCTTCTGGTGCTGCACTATTTCGTTGAATTTCTTTTTCACATTTCTCGTCTCTTTTATTTTAGTGACGAGAAGTATCAGAAAGGGTTTTCTCTCTGGGCAGTTCTCTTTGTTTTGGGAAGACTTCTGACCCTAATTCTTTCAGTTCTTACTGTTGGCTTTGGCCTGGCAAGAGCGGAGAGTCAGAAGCTGGATTTGAGTACTGGAAACTTCAACGTGTTGGCTGTTAGGATCGCTGTTTTGGCGTCCATTTGCATCACACAAGCCTTCATGATGTGGAAGTTCATTAACTTCCAGCTTCGGAGATGGAGGGAACATTCTGGCTTCCAGGCCCCACCTGTGAAAAGGAAACTGGCTGTGACCAAAGGCAGGTCTTCCAGAAAAGGAACAGAGAATGGTGTGAACGGCACAGTCACATCCAATGGAGCGGACTCTCCTCGGAACAGGAAAGAGAAGTCTTCGTAA

>Meriones-unguiculatus

ATGGCGATTCGCAAGAAGAGCAACAAGAACCCGCCGCTGCTGAGCCACGAATTCGTGCTGCAGAATCACGCGGACATCGTGTCCTGCCTGGCGATGCTCTTCCTGCTGGGGCTCATGTTCGAGATAACAGCAAAGGCAGCCATCATCTTCGTTGCTCTTCAGTACAATGTTACCCGCCCTGTAACAGAAGAACAAGCTACTGAATCAGCATCCCTTTATTACTATGGTATCAAAGATTTGGCTACAGTTTTCTTCTACATGCTAGTGGCGATAATTATTCATGCCATAATTCAGGAGTATGTGTTGGATAAAATTAACAGACGAATGCACTTCTCCAAAACAAAACACAGCAAGTTCAATGAGTCTGGCCAGCTTAGCGCATTCTACCTTTTTGCTTGCGTGTGGGGCACATTCATCCTCGTCTCGGAAAACTATATCTCAGATCCAACTATCCTGTGGAGAGCTTATCCCCATAACCTGATGACATTTCAAATGAAGTTTTTCTACATATCCCAGTTGGCTTACTGGCTCCATGCGTTCCCTGAACTCTACTTCCAGAAAACCAAAAAAGAAGATATTCCTCGTCAGCTTGTCTACATCGGTCTTTACCTCTTCCACATCGCTGGAGCATATCTTCTGAACTTGAACCATCTGGGCCTTGTTCTTCTGGTGCTGCACTATTTTGTTGAGTTTCTTTTCCACATTTCTCGTCTATTTTATTTTAGTGATGAGAAATACCAGAAAGGATTTTCTCTCTGGGCGGTTCTTTTTGTTTTGGGAAGACTTCTGACCTTAATTCTTTCGGTCCTCACTGTTGGTTTTGGCCTGGCAAGAGCAGAGAATCAAAAGCTGGATTTTAGTACTGGAAACTTCAATGTGTTGGCAGTCAGGATCGCCGTCCTGGCCTCCATTTGCATCACACAAGCCTTCATGATGTGGAAGTTCATTAACTTCCAGCTTCGGAGGTGGAGGGAGCATTCTGCCTTCCAGGCCCCACCTGTGAAGAGGAAACCAGCCGTGACCAAAGGCAGATCCTCCAGAAAAGGAACAGAGAACGGTGTGAACGGAACCGTCACCTCAAATGGAGCTGACTCTCCGCGGAACCGAAAAGAGAAGTCTTCATAA

>Mus-caroli

ATGGCGATTCGCAAGAAGAGCAACAAGAACCCGCCGCTGCTGAGCCACGAATTCCTGCTGCAGAATCACGCGGACATCGTCTCCTGCCTGGCGATGCTTTTCCTGCTGGGGCTTATGTTCGAGGTAACAGCAAAAGTAGCCATCATCTTTGTTGCTCTTCAGTATAATGTTACCCGCCCTGCAACAGAAGAACAAGCTACTGAATCAGCATCCCTATATCACTATGGTTTCAAAGACTTGGCTACAGTCTCCTTCTACATGCTAGTGGCGATAATTATTCATGCCATAATTCAGGAGTATGTGTTGGATAAAATTAACCGACGGATGCACTTCTCCAAAACGAAGCACAGCAAGTTTAACGAGTCTGGTCAGCTCAGTGCATTCTACCTTTTTGCCTGTGTGTGGGGCACGTTCATTCTCATCTCCGAAAACTATATCTCAGACCCAACTATCCTGTGGAGGGCCTATCCCCATAACCTGATGACATTTCAGATGAAGTTTTTCTACATCTCCCAGCTGGCATACTGGCTCCATGCATTCCCTGAACTCTACTTCCAGAAAACCAAAAAAGAAGATATTCCTCGTCAGCTCGTCTACATTGGCCTTTATCTCTTTCATATTGCTGGAGCCTATCTTCTGAACTTGAACCACCTGGGCCTTGTTCTTCTGGTGCTGCACTATTTCGTTGAGTTTCTTTTCCACATTTCTCGTCTATTTTATTTTAGTGACGAGAAGTATCAGAAAGGGTTTTCTCTCTGGGCAGTTCTTTTTGTTTTGGGAAGACTTCTGACTCTAATTCTTTCAGTTCTTACTGTTGGTTTTGGTCTCGCAAGAGCGGAGAATCAGAAGCTGGACTTTAGTACTGGAAACTTCAACGTGTTGGCTGTTAGGATCGCTGTTCTCGCGTCCATTTGCATCACACAAGCCTTCATGATGTGGAAGTTCATTAACTTCCAGCTCCGGAGGTGGAGGGAACATTCTGCCTTCCAGGCCCCGCCTGTGAAAAGGAAACCGGCCGTGACCAAAGGCAGGTCTTCCAGAAAAGGAACAGAGAACGGTGTGAACGGCACAGTCACATCAAATGGAGCAGACTCGCCTCGGAACAGGAAAGAGAAGTCTTCGTAA

>Mus-musculus

ATGGCGATTCGCAAGAAGAGCAACAAGAACCCGCCGCTGCTGAGCCACGAATTCTTGCTGCAGAATCACGCGGACATCGTCTCCTGCCTGGCGATGCTTTTCCTGCTGGGGCTTATGTTCGAGGTAACAGCAAAAGGAGCCATCATCTTTGTTGCTCTTCAGTATAATGTTACCCGCCCTGCAACAGAAGAACAAGCTACTGAATCAGCATCCCTATATCACTATGGTATCAAAGACTTGGCTACAGTTTTGTTCTACATGCTAGTGGCGATAATTATTCATGCCATAATTCAGGAGTATGTGTTGGATAAAATTAACCGACGGATGCACTTCTCCAAAACGAAGCACAGCAAGTTTAACGAGTCTGGTCAGCTCAGTGCATTCTACCTTTTTGCCTGTGTGTGGGGCACGTTCATTCTCATCTCCGAAAACTATATCTCAGACCCAACTATCCTGTGGAGAGCCTATCCCCATAACCTGATGACATTTCAGACGAAGTTTTTCTACATCTCCCAGCTGGCGTACTGGCTCCATGCATTCCCTGAACTCTACTTCCAGAAAACCAAAAAAGAAGATATCCCTCGTCAGCTCGTCTACATTGGCCTTTATCTCTTTCATATTGCTGGAGCCTATCTTCTGAACTTGAACCACCTGGGCCTTGTCCTTCTGGTGCTGCACTATTTCGTTGAATTTCTTTTCCACATTTCTCGTCTATTTTATTTTAGTGACGAGAAGTATCAGAAAGGGTTTTCTCTCTGGGCAGTTCTTTTTGTTTTGGGAAGACTTCTGACTCTAATTCTTTCAGTTCTTACTGTTGGTTTTGGTCTCGCAAGAGCGGAGAATCAGAAGCTGGACTTTAGTACTGGAAACTTCAACGTGTTGGCCGTTAGGATCGCTGTTCTCGCGTCCATTTGCATCACACAAGCCTTCATGATGTGGAAGTTCATTAACTTCCAGCTTCGGAGGTGGAGGGAACATTCTGCCTTCCAGGCCCCACCTGTGAAAAGGAAACCGGCCGTGACCAAAGGCAGGTCTTCCAGAAAAGGAACAGAGAACGGTGTGAACGGCACAGTCACATCAAACGGAGCAGACTCCCCTCGGAACAGGAAAGAGAAGTCTTCGTAA

>Mus-pahari

ATGGCGATTCGCAAGAAGAGCAACAAGAACCCGCCGCTGCTGAGCCACGAATTCGTGCTGCAGAATCACGCGGACATCGTCTCTTGCCTGGCGATGCTCTTCCTGCTGGGGCTTATGTTCGAGATAACAGCAAAAGCAGCCATCATCTTTGTTGCTCTTCAGTATAATGTTACCCGCCCTGCAACAGAAGAACAAGCTACTGAATCAGCGTCCTTATATCAGTATGGTATCAAAGACTTGGCTACAGTTTTCTTCTACATGCTAGTGGCGATAATTATTCACGCCATAATTCAGGAGTATGTGTTGGATAAAATTAACCGACGGATGCACTTCTCCAAAACGAAGCACAGCAAGTTTAATGAGTCTGGCCAGCTCAGTGCATTCTACCTTTTTGCCTGCGTGTGGGGCACGTTCATTCTCATCTCCGAAAACTATATCTCAGACCCAACTATCCTGTGGAGAGCGTATCCCCATAACCTGATGACATTTCAAATGAAGTTTTTCTACATCTCCCAGCTGGCGTACTGGCTCCATGCATTCCCTGAACTCTACTTCCAGAAAACCAAAAAAGAAGATATTCCTCGTCAGCTCGTCTACATTGGTCTTTACCTCTTTCATATTGCTGGAGCCTATCTTCTGAACTTGAACCATCTGGGCCTTGTTCTTCTGGTGCTGCACTATTTCGTTGAATTTCTTTTCCACATTTCTCGTCTCTTTTATTTCAGTGACGAGAAGTATCAGAAAGGGTTTTCTCTCTGGGCAGTTCTTTTTGTTTTGGGAAGACTTCTGACTCTAATTCTTTCAGTTCTTACTGTTGGTTTTGGTCTTGCAAGAGCAGAGAATCAGAAGCTGGACTTTAGTACTGGAAACTTCAACGTGTTGGCCGTCAGGATCGCTGTTCTCGCGTCCATTTGCATCACACAAGCCTTCATGATGTGGAAGTTCATTAACTTCCAGCTCCGGAGGTGGAGGGAACATTCTGCTTTCCAGGCCCCACCTGTGAAGAGGAAACCGGCCGTGACCAAAGGCAGGTCTTCCAGAAAAGGAACAGAGAACGGTGTGAACGGCACGGTCACATCAAATGGAGCAGACTCTCCTCGGAACAGGAAGGAGAAGTCTTCGTAA

>Rattus-rattus

ATGGCGATTCGCAAGAAGAGCAACAAGAACCCGCCGGTGTTGAGCCATGAATTCGTGCTGCAGAATCACGCGGACATCGTCTCCTGTCTGGCGATGCTCTTCCTGCTGGGGCTTATGTTCGAGATAACAGCAAAAGGAGCCATCATCTTTGTCGCCCTTCAGTACAATGTTACCCGCCCTGCAACAGAAGAACAAGCTGCTGAATCAGCATCCCTTTATTACTATGGTATCAAAGATTTGGCTACAGTGTTCTTCTACATGCTAGTGGCGATAATTGTTCATGCCATAATTCAGGAGTATGTGTTGGATAAAATTAACCGTCGGATGCACTTCTCCAAAACGAAGCACAGCAAGTTTAATGAGTCCGGGCAGCTCAGTGCGTTCTACCTTTTTGCCTGTGTGTGGGGCACATTCATTCTCGTCTCCGAAAACTATATCTCAGACCCGACGATCCTGTGGAGAGCGTATCCCCATAACCTGATGACATTTCAAATGAAGTTTTTCTACATATCCCAGTTGGCATATTGGCTCCATGCATTCCCCGAACTCTACTTCCAGAAAACCAAAAAAGAAGATATTCCTCGCCAGCTTGTCTACATCGGTCTTTACCTCTTCCACATTGCTGGGGCTTACCTTCTGAACTTGAACCATCTGGGCCTTGTTCTTCTGGTGCTGCACTATTTTGTTGAATTTCTTTTCCACATTTCTCGTCTATTTTATTTTAGTGACGAGAAGTACCAGAAAGGGTTTTCTCTTTGGGCGGTTCTTTTTGTTTTGGGAAGACTTCTGACCCTAATTCTTTCAGTCCTCACTGTTGGCTTTGGCCTGGCAAGAGCAGAGAACCAGAAGCTGGACTTCAGTACCGGGAACTTCAATGTGTTGGCGGTTAGGATCGCGGTCCTAGCGTCCATTTGCATCACTCAAGCCTTCATGATGTGGAAGTTCATTAACTTCCAGCTTCGGAGGTGGAGGGAACATTCTGCCTTCCAGGCCCCTCCCGTGAGAAGGAAACCGGCTGTGACCAAAGGCAGGTCCTCCCGAAAAGGAACAGAGAATGGTGTGAACGGCACGGTCACGTCGAATGGAGCCGACTCTCCTCGGAGCAGGAAAGAGAAGTCTTCCTAA

>Musspicilegus

ATGGCGATTCGCAAGAAGAGCAACAAGAACCCGCCGCTGCTGAGCCACGAATTCTTGCTGCAGAATCACGCGGACATCGTCTCCTGCCTGGCGATGCTTTTCCTGCTGGGGCTTATGTTCGAGGTAACAGCAAAAGGAGCCATCATCTTTGTTGCTCTTCAGTATAATGTTACCCGCCCTGCAACAGAAGAACAAGCTACTGAATCAGCATCCCTATATCACTATGGTATCAAAGACTTGGCTACAGTTTTGTTCTACATGCTAGTGGCGATAATTATTCATGCCATAATTCAGGAGTATGTGTTGGATAAAATTAACCGACGGATGCACTTCTCCAAAACGAAGCACAGCAAGTTTAACGAGTCTGGTCAGCTCAGTGCATTCTACCTTTTTGCCTGTGTGTGGGGCATGTTCATTCTCATCTCCGAAAACTATATCTCAGACCCAACTATCCTGTGGAGAGCCTATCCCCATAACCTGATGACATTTCAGACGAAGTTTTTCTACATCTCCCAGCTGGCGTACTGGCTCCATGCATTCCCTGAACTCTACTTCCAGAAAACCAAAAAAGAAGATATCCCTCGTCAGCTCGTCTACATTGGCCTTTATCTCTTTCATATTGCTGGAGCCTATCTTCTGAACTTGAACCACCTGGGCCTTGTTCTTCTGGTGCTGCACTATTTCGTTGAATTTCTTTTCCACATTTCTCGTCTATTTTATTTTAGTGACGAGAAGTATCAGAAAGGGTTTTCTCTCTGGGCAGTTCTTTTTGTTTTGGGAAGACTTCTGACTCTAATACTTTCAGTTCTTACTGTTGGTTTTGGTCTTGCAAGAGCGGAGAATCAGAAGCTGGACTTTAGTACTGGAAACTTCAACGTGTTGGCCGTTAGGATCGCTGTTCTCGCGTCCATTTGCATCACACAAGCCTTCATGATGTGGAAGTTCATTAACTTCCAGCTTCGGAGGTGGAGGGAACATTCTGCCTTCCAGGCCCCACCTGTGAAAAGGAAACCGGCCATGACCAAAGGCAGGTCTTCCAGAAAAGGAACAGAGAACGGTGTGAACGGCACAGTCACATCAAACGGAGCAGACTCCCCTCGGAACAGGAAAGAGAAGTCTTCGTAA

>Psammomys_obesus

ATGGCGATTCGCAAGAAGAGCAACAAGAACCCGCCGCTGCTGAGCCACGAATTCGTGCTGCAGAATCACGCGGACATCGTGTCCTGCCTGGCGATGCTCTTCCTGCTGGGGCTCATGTTCGAGATAACAGCAAAGGCAGCCATCATCTTCGTTGCTCTTCAGTACAATGTTACCCGCCCTGTAACAGAAGAACAAGCTACTGAATCAGCATCCCTTTATTACTATGGTATCAAAGATTTGGCTACAGTTTTCTTCTACATGCTAGTGGCGATAATTATTCATGCCATAATTCAGGAGTATGTGTTGGATAAAATTAACAGACGAATGCACTTCTCCAAAACAAAACACAGCAAGTTCAATGAGTCTGGCCAGCTTAGCGCATTCTACCTTTTTGCTTGCGTGTGGGGCACATTCATCCTCGTCTCGGAAAACTATATCTCAGATCCAACTATCCTGTGGAGAGCTTATCCCCATAACCTGATGACATTTCAGATGAAGTTTTTCTACATATCCCAGTTGGCATACTGGCTCCATGCGTTCCCTGAACTCTACTTCCAGAAAACCAAAAAAGAAGATATTCCTCGTCAGCTTGTCTACATCGGTCTTTACCTCTTCCACATCGCTGGAGCATATCTTCTGAACTTGAACCATCTGGGCCTTGTTCTTCTGGTGCTGCACTATTTTGTTGAGTTTCTTTTCCACATTTCTCGTCTATTTTATTTTAGTGATGAGAAATATCAGAAGGGATTTTCTCTCTGGGCGGTTCTTTTTGTTTTGGGAAGACTTCTGACCTTAATTCTTTCGGTCCTCACTGTTGGTTTTGGCCTGGCAAGAGCAGAGAATCAAAAGCTGGATTTTAGTACTGGAAACTTCAATGTGTTGGCAGTCAGGATCGCCGTCCTGGCCTCCATTTGCATCACACAAGCCTTCATGATGTGGAAGTTCATTAACTTCCAGCTTCGGAGGTGGAGGGAGCATTCTGCCTTCCAGGCCCCACCTGTGAAGAGGAAACCAGCCATGACCAAAGGCAGATCCTCCAGAAAAGGAACAGAGAACGGTGTGAATGGAACAGTCACATCAAATGGAGCTGACTCTCCGCGGAATAGAAAAGAGAAGTCTTCATAA

Supplementary Data 5. Aligned sequences of MyD88

>Rattus-norvegicus

ATGTCCGAGGAGCTTTCGGCGGACACGTCCTACACGGAAGATGATTTCTACTGCCCAGTCTGTCAGGAGGTGCTCAAGACGCCGGTGCGGACCGCGGCCTGTCAGCACGTTTTCTGTAGAAAATGTTTCCTGACTGCAATGAGAGAGAGTGGAATACATTGTCCCCTATGTCGCGGAAGTGTGACTAGAAGAGAAAGAGCATGTCCGGAACGGGCCATAGATCTTGAAAATATCATGAGGAGGGTTTCTGGTAGCTGCAGATGCTGTTCAAAAAAGATTAAATTTTATCGCATGAGACATCATTACAAATCTTGTAAGAAGTATCAGGATGAATATGGTGTCTCTTCTGTCATTCCAAATGTTAAGATTTCTCAAGATTCAGTAAGGAGCAGTAATAGGAGTGAAACATCTGCATCTGATAACACAGAAACTTATCAAGAGGATACAAGTTCTTCTGGGCATCCCACCTTTAAGTGTCCCTTATGTCAAGAGTCAAATTTCACCAGACAACGTTTATTGGATCACTGTAATAGTAACCACCTGTTTCAGATAGTTCCTGTGAATCTTCAGCTAGATGAGGAAACCCAATATCAAACTGCTGTGGAAGAGTCTTTCCAAGTTAACATGTGA

>Rattus-tanezumi

ATGTCCGAGGAGCTTTCGGCGGACACGTCCTACACGGAAGATGATTTCTACTGCCCAGTCTGTCAGGAGGTGCTCAAGACGCCGGTGCGGACCGCGGCCTGTCAGCACGTTTTCTGTAGAAAATGTTTCCTGACTGCAATGAGAGAGAGTGGAATACATTGTCCCCTATGTCGCGGAAGTGTGACTAGAAGAGAAAGAGCATGTCCGGAACGGGCCATAGATCTTGAAAATATCATGAGGAGGGTTTCTGGTAGCTGCAGATGCTGTTCAAAAAAGATTAAATTTTATCGCATGAGACATCATTACAAATCTTGTAAGAAGTATCAGGATGAATATGGTGTCTCTTCTGTCATTCCAAATGTTAAGATTTCTCAAGATTCAGTAAGGAGCAGTAATAGGAGTGAAACATCTGCATCTGATAACACAGAAACTTATCAAGAGGATACAAGTTCTTCTGGGCATCCCACCTTTAAGTGTCCCTTATGTCAAGAGTCAAATTTCACCAGACAACGTTTATTGGATCACTGTAATAGTAACCACCTATTTCAGATAGTTCCTGTGAATCTTCAGCTAGATGAGGAAACCCAATATCAAACTGCTGTGGAAGAGTCTTTCCAAGTTAACATGTGA

>Acomys-russatus

ATGTCCGAGGAGCTTTCGGCGGCCACGTCCTACACGGAAGATGACTTCTACTGCCCTGTCTGTCAGGAGGTGCTCAAGACGCCGGTGCGGACCGCGGCCTGCCAGCACGTTTTCTGTAGAAAATGTTTCCTGACTGCAATGAGAGAAAGTGGAATACATTGTCCCCTATGTCGTGGAAGTGTGACCAGAAGAGAAAGAGCATGTCCGGAACGGGCCTTAGATCTTGAAAATATCATGAGGAAATTTTCTGGTAGCTGCAGATGCTGTTCAAAAAAGATTAAATTCTATCGCATGAGACATCATTACAAATCTTGTAAGAAGTATCAGGATGAATATGGTGTTTCTTCTGTCATTCCAAACTTTAAGGTTTCTCAAGATTCAGTAAGGAACAGTAATAGGAGTGAAACATCTACATCTGATAATACAGAAACTTACCAAGAGAATACAAGTTCTTCTGGGCATCCCACCTTTAAGTGTCCCTTATGTCAAGAGTCAAATTTCACCAGACAGCGTTTACTGGATCACTGTAATAGTAACCACCTATTTCAGATAGTTCCTGTGAATCTTCAGCTGGATGAGGAAACCCAATATCAAACTGCGGTGGAAGAGTCTTTTCAAGTGAACATCTGA

>Apodemus-sylvaticus

ATGTCCGAGGAGCTTTCGGCGGCCACGTCCTACACGGAGGACGATTTCTACTGCCCCGTCTGTCAGGAGGTGCTCAAGACGCCGGTGCGGACCGCGGCCTGTCAGCACGTTTTCTGTAGAAAATGTTTCCTGACTGCAATGAGAGAAAGTGGAATACATTGTCCCCTATGTCGTGGAAGCGTGACTAGAAGAGAAAGAGCATGTCCGGAACGGGCCTTAGATCTTGAAAATATCATGAGGAGGTTTTCTGGTAGCTGCAGATGCTGTTCAAAAAAGATTAAATTCTATCGCATGAGACATCATTACAAATCTTGTAAGAAGTATCAGGATGAATATGGTGTTTCTTCTGTCATTCCAAACTTTAAGATTTCTCAAGATTCAGTAAGGAACAGTAATAGGAGTGAAACATCTGCATCTGATAACACAGAAACTTATCAAGAGGATACAAGTTCTTCTGGGCATCCCACCTTTAAGTGTCCCTTATGTCAAGAGTCAAATTTCACCAGACAACGTTTATTGGATCACTGTAATAGTAACCACCTATTTCAGATAGTTCCTGTGAATCTTCAGCTAGATGAGGAAACCCAATATCAAACTGCTGTGGAAGAGTCTTTTCAAGTAAACATGTGA

>Arvicanthis-niloticus

ATGTCCGAGGAGCTGTCGGCGGCCACGTCCTACACGGAAGATGATTTCTACTGCCCTGTCTGTCAGGAGGTGCTCAAGACGCCGGTGCGGACCGCGGCCTGTCAGCACGTTTTCTGTAGAAAATGTTTCCTGACTGCAATGAGAGAAAGTGGAATACATTGTCCCTTATGTCGTGGAAGTGTGACTAGAAGAGAAAGAGCATGTCCAGAACGGGCCTTAGATCTTGAAAATATCATGAGGAGGTTTTCTGGTAGCTGCAGATGCTGTTCAAAAAAGATTAAATTCTATCGCATGAGACATCATTACAAATCTTGTAAGAAGTATCAGGATGAATATGGTGTTTCTTCTGTCATTCCAAACTTTAAGATTTCTCAAGATTCAGTAAGGAGCAGTAATAGGAGTGAAACATCTGCATCTGATAACACAGAAACTTATCAAGAGGATACAAGTTCTTCTGGGCATCCCACCTTTAAGTGTCCCTTATGTCAAGAGTCAAATTTCACCAGACAACGTTTATTGGATCACTGTAATAGTAACCACCTATTTCAGATAGTTCCTGTGAATCTTCAGCTAGATGAGGAAACCCAATATCAAACTGCTGTGGAAGAGTCTTTTCAAGTAAACATGTGA

>Grammomys-surdaster

ATGTCCGAGGATCTGTCGGCGGCCACGTCCTACACGGAAGATGATTTCTACTGCCCTGTCTGTCAGGAGGTGCTCAAGACGCCGGTGCGGACCGCGGCCTGTCAGCACGTTTTCTGTAGAAAATGTTTCCTGACTGCAATGAGAGAAAGTGGAATACACTGTCCCTTATGTCGTGGAAGTGTGACTAGAAGAGAAAGAGCATGTCCAGAACGGGCCTTAGATCTTGAAAATATCATGAGGAGGTTTTCTGGTAGCTGCAGATGCTGTTCAAAAAAGATTAAATTCTATCGCATGAGACATCATTACAAATCTTGTAAGAAGTATCAGGATGAATATGGTGTTTCTTCTGTCATTCCAAACTTTAAGATTTCTCAAGATTCAGTAAGGAGCAGTAATAGGAATGAAACATCTGCATCTGATAACACAGAAACTTATCAAGAGGATACAAGTTCTTCTGGGCATCCCACCTTTAAGTGTCCCTTATGTCAAGAGTCAAATTTCACCAGACAACGTTTATTGGATCACTGTAATAGTAACCACCTATTTCAGATAGTTCCTGTGAATCTTCAGCTAGATGAGGAAACCCAATATCAAACTGCTGTGGAAGAGTCTTTTCAAGTAAACATGTGA

>Mastomys-coucha

ATGTCCGAGGAGCTCTCGGAGGCCACGTCCTACACGGAAGACGATTTCTACTGCCCCGTCTGTCAGGAGGTGCTCAAGACGCCGGTGCGGACCGCGGCCTGTCAGCACGTTTTCTGTAGAAAATGTTTCCTGACTGCAATGAGAGAAAGTGGAATACATTGTCCCCTATGTCGTGGAAGTGTGACTAGAAGAGAAAGAGCATGTCCTGAACGGGCCTTAGATCTTGAAAATATCATGAGGAGGTTTTCTGGTAGCTGCAGATGCTGTTCAAAAAAGATTAAATTCTATCGCATGAGACATCATTACAAATCTTGTAAGAAGTATCAGGATGAATATGGTGTTTCTTCTGTCATTCCAAACTTTAAGATTTCTCAAGATTCAGAAAGGAGCAGTAATAGGAGTGAAACATCTGCATCTGATAACATGGAAACTTATCAAGAGGATACAAGTTCTTCTGGGCATCCCACCTTTAAGTGTCCCTTATGTCAAGAGTCAAATTTCACCAGACAACGTCTATTGGATCACTGTAATAGTAACCACCTATTTCAGATAGTTCCTGTGAATCTTCAGCTAGATGAGGAGACCCAATATCAAACTGCTGTGGAAGAGTCTTTTCAAGTAAACTTGTGA

>Meriones-unguiculatus

ATGTCCGAGGAGCTCTCGGCGGCCACGTCCTACACGGAAGATGACTTTTACTGCCCTGTGTGTCAGGAGGTGCTCAAGACGCCGGTGCGGACCGCGGCCTGCCAGCACGTTTTCTGTAGAAAATGTTTCCTGACTGCAATGAGAGAAAGTGGAATACATTGTCCCCTATGTCGTGGAAGTGTGACTAGAAGAGAAAGAGCATGTCCGGAACGGGCCTTAGATCTTGAAAATATCATGAGGAAGTTTTCTGGTAGCTGCAGATGCTGTTCAAAAAAGATTAAATTCTATCGCATGAGACATCATTACAAATCTTGTAAGAAGTATCAGGATGAATATGGTGTTTCTTCTGGCATTCAGAACTTTAAGATTTCTCAAGACTCAGTAAGGAACAGTAATAGGAGTGAAACATCTACATCTGATAACACAGAAACTTATCAAGAGAATACAAGTTCTTCTGGGCATCCCACCTTTAAGTGTCCCTTATGTCAAGAATCAAATTTCACCAGACAGCGTTTATTGGATCACTGTAATAGTAACCACCTATTTCAGATAGTTCCTGTGAATCTTCAGCTAGATGAAGAAACCCAATATCAAACTGCTGTGGAAGAGTCTTTTCAAGTAAACATCTGA

>Mus-caroli

ATGTCCGAGGAACTTTCGGCGGCCACGTCCTACACGGAAGATGATTTCTACTGCCCTGTCTGTCAGGAGGTGCTCAAGACGCCGGTGCGGACCGCGGCCTGTCAGCACGTTTTCTGTAGAAAATGTTTCCTGACTGCAATGAGAGAAAGTGGAATACATTGTCCCCTATGTCGTGGAAGTGTGACTAGAAGAGAAAGAGCATGTCCGGAACGGGCCTTAGATCTTGAAAATATCATGAGGAGGTTTTCTGGTAGCTGCAGATGCTGTTCAAAAAAGATTAAATTCTATCGCATGAGACATCATTACAAATCTTGTAAGAAGTATCAGGATGAATATGGTGTTTCTTCTGTCATTCCAAACTTTAAGATTTCTCAAGATTCAGTAAGGAGCAGTAATAGGAGTGAAACATCTGCATCTGATAACACAGAAACTTATCAAGAGGATACAAGTTCTTCTGGGCATCCCACCTTTAAGTGTCCCTTATGTCAAGAGTCAAATTTCACCAGACAACGTTTATTGGATCACTGTAATAGTAACCACCTATTTCAGATAGTTCCTGTGAATCTTCAGCTAGATGAGGAAACCCAATATCAAACTGCTGTGGAAGAGTCTTTTCAAGTAAACATGTGA

>Mus-musculus

ATGTCCGAGGAACTTTCGGCGGCCACGTCCTACACGGAAGATGATTTCTACTGCCCTGTCTGTCAGGAGGTGCTCAAGACGCCGGTGCGGACCGCGGCCTGTCAGCACGTTTTCTGTAGAAAATGTTTCCTGACTGCAATGAGAGAAAGTGGAATACATTGTCCCCTATGTCGTGGAAGTGTGACTAGAAGAGAAAGAGCATGTCCGGAACGGGCCTTAGATCTTGAAAATATCATGAGGAGGTTTTCTGGTAGCTGCAGATGCTGTTCAAAAAAGATTAAATTCTATCGCATGAGACATCATTACAAATCTTGTAAGAAGTATCAGGATGAATATGGTGTTTCTTCTGTCATTCCAAACTTTAAGATTTCTCAAGATTCAGTAAGGAGCAGTAATAGGAGTGAAACATCTGCATCTGATAACACAGAAACTTATCAAGAGGATACAAGTTCTTCTGGGCATCCTACCTTTAAGTGTCCCTTATGTCAAGAGTCAAATTTCACCAGACAACGTTTATTGGATCACTGTAATAGTAACCACCTATTTCAGATAGTTCCTGTGAATCTTCAGCTAGATGAGGAAACCCAATATCAAACTGCTGTGGAAGAGTCTTTTCAAGTAAACATGTGA

>Mus-pahari

ATGTCCGAGGACCTTTCGGCGGCCACGTCCTACACGGAAGATGATTTCTACTGCCCTGTCTGTCAGGAGGTGCTCAAGACGCCGGTGCGGACCGCGGCCTGTCAGCACGTTTTCTGTAGAAAATGTTTCCTGACTGCAATGAGAGAAAGTGGAATACATTGTCCCCTATGTCGTGGAAGTGTGACTAGAAGAGAAAGAGCATGTCCGGAACGGGCCTTAGATCTTGAAAATATCATGAGGAGGTTTTCTGGTAGCTGCAGATGCTGTTCAAAAAAGATTAAATTCTATCGCATGAGACATCATTACAAATCTTGTAAGAAGTATCAGGATGAATATGGTGTTTCTTCTGTCATTCCAAACTTTAAGATTTCTCAAGATTCAGTAAGGAGCAGTAATAGGAGTGAAACGTCTGCATCTGATAACACAGAAACTTATCAAGAGGATACAAGTTCTTCTGGGCATCCCACCTTTAAGTGTCCCTTATGTCAAGAGTCAAATTTCACCAGACAACGTTTATTGGATCACTGTAATAGTAACCACCTATTTCAGATAGTTCCTGTGAATCTTCAGCTAGATGAGGAAACCCAATATCAAACTGCTGTGGAAGAGTCTTTTCAAGTAAGCATGTGA

>Rattus-rattus

ATGTCCGAGGAGCTTTCGGCGGACACGTCCTACACGGAAGATGATTTCTACTGCCCAGTCTGTCAGGAGGTGCTCAAGACGCCGGTGCGGACCGCGGCCTGTCAGCACGTTTTCTGTAGAAAATGTTTCCTGACTGCAATGAGAGAGAGTGGAATACATTGTCCTCTATGTCGCGGAAGTGTGACTAGAAGAGAAAGAGCATGTCCGGAACGGGCCATAGATCTTGAAAATATCATGAGGAGGGTTTCTGGTAGCTGCAGATGCTGTTCAAAAAAGATTAAATTTTATCGCATGAGACATCATTACAAATCTTGTAAGAAGTATCAGGATGAATATGGTGTCTCTTCTGTCATTCCAAATGTTAAGATTTCTCAAGATTCAGTAAGGAGCAGTAATAGGAGTGAAACATCTGCATCTGATAACACAGAAACTTATCAAGAGGATACAAGTTCTTCTGGGCATCCCACCTTTAAGTGTCCCTTATGTCAAGAGTCAAATTTCACCAGACAACGTTTATTGGATCACTGTAATAGTAACCACCTATTTCAGATAGTTCCTGTGAATCTTCAGCTAGATGAGGAAACCCAATATCAAACTGCTGTGGAAGAGTCTTTCCAAGTTAACATGTGA

Supplementary Table S1. Sample information and sequencing statistics.

| **Sample** | **Species** | **Locality** | **Sex** | **Clean Reads** | **Mapped Bases** | **Mapping Rate (%)** | **Mean Depth** | **Reference Genome**  **Coverage (>1×) (%)** |
| --- | --- | --- | --- | --- | --- | --- | --- | --- |
| LS1 | *Rattus tanezumi* | Lasa, Tibet (LS) | M | 282,763,712 | 279,949,657 | 99 | 14.37 | 98.18 |
| LS2 | *Rattus tanezumi* | Lasa, Tibet (LS) | F | 297,508,756 | 294,319,665 | 98.93 | 15.04 | 98.18 |
| LS3 | *Rattus tanezumi* | Lasa, Tibet (LS) | M | 326,480,064 | 322,952,503 | 98.92 | 16.71 | 98.23 |
| LS4 | *Rattus tanezumi* | Lasa, Tibet (LS) | F | 317,508,128 | 314,323,066 | 99 | 16.36 | 98.35 |
| LS5 | *Rattus tanezumi* | Lasa, Tibet (LS) | F | 365,155,070 | 361,343,540 | 98.96 | 18.47 | 98.22 |
| LS6 | *Rattus tanezumi* | Lasa, Tibet (LS) | M | 351,827,526 | 347,979,019 | 98.91 | 18.18 | 98.21 |
| LS7 | *Rattus tanezumi* | Lasa, Tibet (LS) | M | 281,685,332 | 278,845,087 | 98.99 | 14.38 | 98.31 |
| LS8 | *Rattus tanezumi* | Lasa, Tibet (LS) | M | 286,761,610 | 283,400,921 | 98.83 | 14.72 | 98.16 |
| MH | *Rattus tanezumi* | Menghai, Yunnan (MH) | F | 307,738,552 | 304,693,728 | 99.01 | 14.26 | 97.35 |
| CD1 | *Rattus tanezumi* | Chengdu, Sichuan (CD) | M | 290,129,108 | 285,673,635 | 98.46 | 14.2 | 98.59 |
| CD2 | *Rattus tanezumi* | Chengdu, Sichuan (CD) | M | 323,986,768 | 319,292,345 | 98.55 | 16.18 | 98.53 |
| GZ1 | *Rattus tanezumi* | Guangzhou, Guangdong (GZ) | M | 288,864,986 | 283,881,984 | 98.28 | 14.65 | 98.69 |
| GZ2 | *Rattus tanezumi* | Guangzhou, Guangdong (GZ) | M | 299,296,366 | 296,138,012 | 98.94 | 14.26 | 98.62 |
| CS1 | *Rattus tanezumi* | Changsha, Hunan (CS) | M | 321,978,726 | 319,282,316 | 99.16 | 15.39 | 98.28 |
| CS2 | *Rattus tanezumi* | Changsha, Hunan (CS) | M | 279,478,362 | 277,327,519 | 99.23 | 13.48 | 98.22 |
| CS3 | *Rattus tanezumi* | Changsha, Hunan (CS) | F | 307,181,142 | 304,959,534 | 99.28 | 14.62 | 98.46 |
| WZ1 | *Rattus tanezumi* | Wenzhou, Zhejiang (WZ) | M | 300,798,294 | 297,266,482 | 98.83 | 14.88 | 98.5 |
| WZ2 | *Rattus tanezumi* | Wenzhou, Zhejiang (WZ) | M | 326,322,128 | 322,246,475 | 98.75 | 15.87 | 98.51 |
| SH | *Rattus tanezumi* | Shanghai (SH) | M | 388,797,162 | 384,357,320 | 98.86 | 18.63 | 98.25 |
| YC | *Rattus tanezumi* | Yichang, Hubei (YC) | F | 285,550,870 | 283,834,539 | 99.4 | 12.72 | 98.18 |
| ZK1 | *Rattus tanezumi* | Zhoukou, Henan (ZK) | F | 445,679,680 | 442,109,954 | 99.2 | 18.61 | 97.9 |
| ZK2 | *Rattus tanezumi* | Zhoukou, Henan (ZK) | F | 329,927,292 | 327,081,593 | 99.14 | 15.94 | 98.38 |
| SJZ | *Rattus tanezumi* | Shijiazhuang, Hebei (SJZ) | M | 306,114,124 | 304,296,314 | 99.41 | 15.28 | 98.45 |
| BD1 | *Rattus tanezumi* | Baoding, Hebei (BD) | M | 293,771,844 | 290,036,786 | 98.73 | 14.78 | 98.39 |
| BD2 | *Rattus tanezumi* | Baoding, Hebei (BD) | M | 262,845,312 | 261,033,872 | 99.31 | 13.28 | 98.17 |
| TJ | *Rattus tanezumi* | Tianjin (TJ) | M | 224,236,478 | 221,751,891 | 98.89 | 11.52 | 98.53 |
| LF | *Rattus tanezumi* | Linfen, Shanxi (HT) | M | 297,136,478 | 294,535,125 | 99.12 | 13.95 | 98.43 |
| TY | *Rattus tanezumi* | Taiyuan, Shanxi (TY) | M | 290,681,610 | 288,909,528 | 99.39 | 12.69 | 99.2 |
| XA1 | *Rattus tanezumi* | Xi’an, Shaanxi (XA) | F | 261,300,702 | 259,534,475 | 99.32 | 13.16 | 98.52 |
| XA2 | *Rattus tanezumi* | Xi’an, Shaanxi (XA) | M | 265,143,014 | 263,252,713 | 99.29 | 13.58 | 98.49 |
| XN1 | *Rattus tanezumi* | Xining, Qinghai (XN) | M | 329,504,192 | 325,160,655 | 98.68 | 16.57 | 97.24 |
| XN2 | *Rattus tanezumi* | Xining, Qinghai (XN) | M | 314,519,146 | 312,207,960 | 99.27 | 14.81 | 98.51 |
| XN3 | *Rattus tanezumi* | Xining, Qinghai (XN) | F | 376526850 | 372569675 | 98.95 | 19.32 | 98.62 |
| KLMYf20 | *Rattus* *norvegicus* | Kelamayi, Xinjiang Uygur Autonomous Region, P.R.China | F | 218,295,056 | 31,844,567,591 | 97.25 | 12.22 | 98.77 |
| KLMYf23 | *Rattus* *norvegicus* | Kelamayi, Xinjiang Uygur Autonomous Region, P.R.China | F | 239,532,950 | 34,973,346,939 | 97.34 | 13.37 | 98.92 |
| SWf16 | *Rattus* *norvegicus* | Shawan, Xinjiang Uygur Autonomous Region, P.R.China | F | 284,874,092 | 41,595,749,758 | 97.34 | 15.93 | 99.07 |
| SWf19 | *Rattus* *norvegicus* | Shawan, Xinjiang Uygur Autonomous Region, P.R.China | F | 260,198,392 | 38,037,791,869 | 97.46 | 14.57 | 99.02 |
| JCf15 | *Rattus* *norvegicus* | Changji, Xinjiang Uygur Autonomous Region, P.R.China | F | 304,283,712 | 44,462,792,009 | 97.42 | 17.00 | 99.13 |
| JCm18 | *Rattus* *norvegicus* | Changji, Xinjiang Uygur Autonomous Region, P.R.China | M | 257,888,888 | 37,575,639,170 | 97.14 | 14.44 | 98.94 |
| JCm26 | *Rattus* *norvegicus* | Changji, Xinjiang Uygur Autonomous Region, P.R.China | M | 259,039,128 | 37,681,989,857 | 96.98 | 14.51 | 99 |
| WSf11 | *Rattus* *norvegicus* | Urumqi, Xinjiang Uygur Autonomous Region, P.R.China | F | 225,021,402 | 32,867,298,900 | 97.38 | 12.60 | 98.85 |
| WSm10 | *Rattus* *norvegicus* | Urumqi, Xinjiang Uygur Autonomous Region, P.R.China | M | 338,243,022 | 49,347,132,674 | 97.26 | 18.90 | 99.23 |
| WSm1 | *Rattus* *norvegicus* | Urumqi, Xinjiang Uygur Autonomous Region, P.R.China | M | 277,130,762 | 40,414,670,744 | 97.22 | 15.40 | 99.05 |
| WSm39 | *Rattus* *norvegicus* | Urumqi, Xinjiang Uygur Autonomous Region, P.R.China | M | 290,631,222 | 42,353,178,334 | 97.15 | 16.25 | 99.11 |
| WSm8 | *Rattus* *norvegicus* | Urumqi, Xinjiang Uygur Autonomous Region, P.R.China | M | 294,012,276 | 42,846,861,525 | 97.15 | 16.44 | 99.07 |
| WJQf14 | *Rattus* *norvegicus* | Wujiaqu, Xinjiang Uygur Autonomous Region, P.R.China | F | 259,318,340 | 37,862,797,304 | 97.34 | 14.50 | 99 |
| WJQm12 | *Rattus* *norvegicus* | Wujiaqu, Xinjiang Uygur Autonomous Region, P.R.China | M | 297,868,198 | 43,438,302,627 | 97.22 | 16.66 | 99.22 |
| TLFf29 | *Rattus* *norvegicus* | Tulufan, Xinjiang Uygur Autonomous Region, P.R.China | F | 297,353,340 | 43,424,875,546 | 97.36 | 16.62 | 99.05 |
| TLFm30 | *Rattus* *norvegicus* | Tulufan, Xinjiang Uygur Autonomous Region, P.R.China | M | 241,902,740 | 35,049,362,660 | 96.59 | 13.37 | 98.99 |
| LZf43 | *Rattus* *norvegicus* | Lanzhou, Gansu Province, P.R.China | F | 228,569,396 | 33,185,220,018 | 96.79 | 12.73 | 99.05 |
| LZm42 | *Rattus* *norvegicus* | Lanzhou, Gansu Province, P.R.China | M | 318,747,356 | 46,203,560,108 | 96.64 | 17.72 | 99.19 |
| BTf1 | *Rattus* *norvegicus* | Baotou, Inner Mongolia Autonomous Region, P.R.China | F | 253,359,024 | 36,731,807,228 | 96.65 | 14.02 | 99.11 |
| HSf1 | *Rattus* *norvegicus* | Huhhot, Inner Mongolia Autonomous Region, P.R.China | F | 231,586,072 | 33,573,544,812 | 96.65 | 12.89 | 98.77 |
| NKf1 | *Rattus* *norvegicus* | Beijing, P.R.China | F | 284,164,786 | 41,533,884,007 | 97.44 | 15.93 | 98.99 |
| XTSm1 | *Rattus* *norvegicus* | Beijing, P.R.China | M | 312,091,200 | 45,429,360,290 | 97.04 | 17.41 | 99.19 |
| BJm10 | *Rattus* *norvegicus* | Beijing, P.R.China | M | 257,879,898 | 37,299,669,050 | 96.43 | 14.35 | 99.2 |
| BJm4 | *Rattus* *norvegicus* | Beijing, P.R.China | M | 231878942 | 33567149244 | 96.51 | 12.98 | 99.04 |
| BJm9 | *Rattus* *norvegicus* | Beijing, P.R.China | M | 219112308 | 31704106860 | 96.46 | 12.23 | 99.1 |
| BJm3 | *Rattus* *norvegicus* | Beijing, P.R.China | M | 389281544 | 47076576717 | 93.85 | 16.18 | 98.82 |
| DXm1 | *Rattus* *norvegicus* | Beijing, P.R.China | M | 340628120 | 49251048063 | 96.39 | 18.90 | 99.34 |
| ZZm1 | *Rattus* *norvegicus* | Baoding, Hebei Province, P.R.China | M | 303624020 | 44205878953 | 97.06 | 16.97 | 99.23 |
| GBDm1 | *Rattus* *norvegicus* | Baoding, Hebei Province, P.R.China | M | 248418132 | 35960435457 | 96.51 | 13.77 | 98.97 |
| TSm1 | *Rattus* *norvegicus* | Tangshan, Hebei Province, P.R.China | M | 247159274 | 36042733850 | 97.22 | 13.68 | 99.02 |
| TJm4 | *Rattus* *norvegicus* | Tianjin, P.R.China | M | 201944142 | 29251167387 | 96.57 | 11.27 | 99.01 |
| ZPf14 | *Rattus* *norvegicus* | Binzhou, Shandong Province, P.R.China | F | 248320530 | 36251864485 | 97.33 | 13.92 | 99.03 |
| ERS215797 | *Rattus* *norvegicus* | Harbin, Heilongjiang Province, P.R.China | M | 717078215 | 70725385817 | 99.84 | 24.31 | 98.92 |
| ERS215799 | *Rattus* *norvegicus* | Harbin, Heilongjiang Province, P.R.China | F | 630470497 | 62174922846 | 99.83 | 21.37 | 98.86 |
| ERS215800 | *Rattus* *norvegicus* | Harbin, Heilongjiang Province, P.R.China | F | 709893323 | 69594922683 | 99.23 | 23.92 | 98.93 |
| HrbPeter | *Rattus* *norvegicus* | Harbin, Heilongjiang Province, P.R.China | F | 1267503343 | 1.20199E+11 | 95.34 | 41.31 | 99.06 |
| HESm1 | *Rattus* *norvegicus* | Jinzhou, Liaoning Province, P.R.China | M | 212658846 | 30672287146 | 96.15 | 11.80 | 99 |
| HESm2 | *Rattus* *norvegicus* | Jinzhou, Liaoning Province, P.R.China | M | 203335760 | 29365607346 | 96.28 | 11.32 | 98.9 |
| TILm1 | *Rattus* *norvegicus* | Tieling, Liaoning Province, P.R.China | M | 224114170 | 32489342539 | 96.65 | 12.54 | 98.94 |
| NYm1 | *Rattus* *norvegicus* | Nanyang, Henan Province, P.R.China | M | 576323865 | 71642774669 | 93.29 | 24.62 | 98.94 |
| NYm2 | *Rattus* *norvegicus* | Nanyang, Henan Province, P.R.China | M | 491309325 | 59955244299 | 93.91 | 20.61 | 98.95 |
| NYm3 | *Rattus* *norvegicus* | Nanyang, Henan Province, P.R.China | M | 581926414 | 72480146793 | 93.67 | 24.91 | 98.99 |
| NYm4 | *Rattus* *norvegicus* | Nanyang, Henan Province, P.R.China | M | 448033201 | 53587335205 | 94.25 | 18.42 | 98.86 |
| WHf41 | *Rattus* *norvegicus* | Wuhan, Hubei Province, P.R.China | F | 265731964 | 38578805736 | 96.79 | 14.82 | 99.18 |
| WHm40 | *Rattus* *norvegicus* | Wuhan, Hubei Province, P.R.China | M | 249640704 | 36162123526 | 96.57 | 13.88 | 99.04 |
| GDm4 | *Rattus* *norvegicus* | Guangzhou, Guangdong Province, P.R.China | M | 527739986 | 65433672685 | 93.50 | 22.49 | 98.98 |
| SHf32 | *Rattus* *norvegicus* | Shanghai, P.R.China | F | 241159198 | 34952172529 | 96.62 | 13.41 | 98.98 |
| SHm29 | *Rattus* *norvegicus* | Shanghai, P.R.China | M | 291476308 | 42235856609 | 96.60 | 16.19 | 99.24 |
| DJYm2 | *Rattus* *norvegicus* | Chengdu, Sichuan Province, P.R.China | M | 257803807 | 28129230864 | 94.08 | 9.67 | 98.58 |
| YZm3 | *Rattus* *norvegicus* | Yangzhou, Jiangsu Province, P.R.China | M | 451243189 | 55953666509 | 93.06 | 19.23 | 98.93 |
